# Counterion‐Mediated Luminophore Dimerization

**DOI:** 10.1002/anie.202505433

**Published:** 2025-06-25

**Authors:** Ash G. Carter, Promeet K. Saha, Antara Sikder, Juan A. Aguilar, Andrew P. Monkman, Alyssa‐Jennifer Avestro, Marc K. Etherington, Paul R. McGonigal

**Affiliations:** ^1^ Department of Chemistry University of York Heslington YO10 5DD UK; ^2^ Department of Chemistry Durham University Durham DH1 3LE UK; ^3^ Department of Physics Durham University Durham DH1 3LE UK; ^4^ Department of Mathematics Physics and Electrical Engineering Northumbria University Newcastle upon Tyne NE1 8ST UK; ^5^ Department of Chemistry University of Oxford Oxford OX1 3TA UK

**Keywords:** Aggregation, Dimers, Excimers, Fluorescence, Noncovalent interactions

## Abstract

Emissive organic salts have long been integral to the discovery of fluorescence phenomena and functional luminescent dyes. Typically, one component of the salt acts as the photoactive unit (luminophore) and its nonemissive counterion is selected to independently tune bulk physical properties, such as solubility. However, the impact of counterion choice on the aggregation and resulting emissive state of organic salts in solution has not been widely investigated. Here, we report that a single cationic luminophore gives rise to either monomer, dimer, excimer, or multichromatic emission under otherwise identical conditions by varying only its counterion. We employ *N*‐methyl quininium (**MeQn** ^+^) as a permanently charged cationic luminophore, which we pair with a series of monovalent anions. At low solution‐state concentrations, all the salts give identical absorption and emission spectra that correlate with the **MeQn** ^+^ monomer. However, at higher concentrations, the emission, excitation, and absorption data differ, revealing the presence of monomer, dimer, excimer, or all three, depending on the structure of the anion. Understanding and modulating the formation of dimeric or other well‐defined aggregated species by specific ion effects could be exploited in the design of molecular probes for biological systems or emissive thin‐film dispersions for optoelectronic devices.

## Graphical abstract

The choice of anion selectively directs the solution‐state aggregation of a cationic luminophore, dictating its photoluminescence. Some anions cause emission from a monomeric state, some induce excimer formation, and others lead to dimers, while the triflate counterion gives multichromatic emission from all three species.

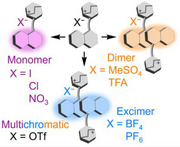

Materials chemists have many well‐established luminophores at their disposal, encompassing organic, organometallic, or inorganic compounds in ionic, zwitterionic, or neutral states. Throughout the long history of synthetic fluorescent dyes, organic salts have played important roles in the discovery of fundamental photophysical phenomena, such as H‐ and J‐aggregation.^[^ ^1^, ^2^, ^3^, ^4^, ^5^ ^]^

Noncovalent dimers are particularly valuable model systems^[^ ^6^, ^7^ ^]^ for aggregated species as they provide tractable information about how through‐space interactions between luminophores influence their photophysics, which can then be applied to the design of molecular probes,^[^ ^8^, ^9^ ^]^ or extrapolated to the higher‐order aggregates and assemblies used in device applications. The diverse photophysical properties of dimers^[^ ^6^, ^7^, ^10^, ^11^ ^]^ (Figure 1) and aggregates^[^ ^12^, ^13^, ^14^, ^15^ ^]^ have been investigated in environments with long‐range order, such as crystalline^[^ ^16^, ^17^, ^18^, ^19^, ^20^, ^21^, ^22^, ^23^, ^24^ ^]^ and liquid crystalline phases.^[^ ^25^, ^26^ ^]^ They have also been engineered in discrete systems by exploiting directional noncovalent bonding interactions (e.g., hydrogen bonding^[^ ^27^, ^28^ ^]^ and halogen bonding^[^ ^29^ ^]^) in the contexts of supramolecular inclusion complexes^[^ ^30^, ^31^, ^32^, ^33^ ^]^ and mechanically interlocked molecules.^[^ ^34^, ^35^, ^36^, ^37^, ^38^ ^]^

**Figure 1:**
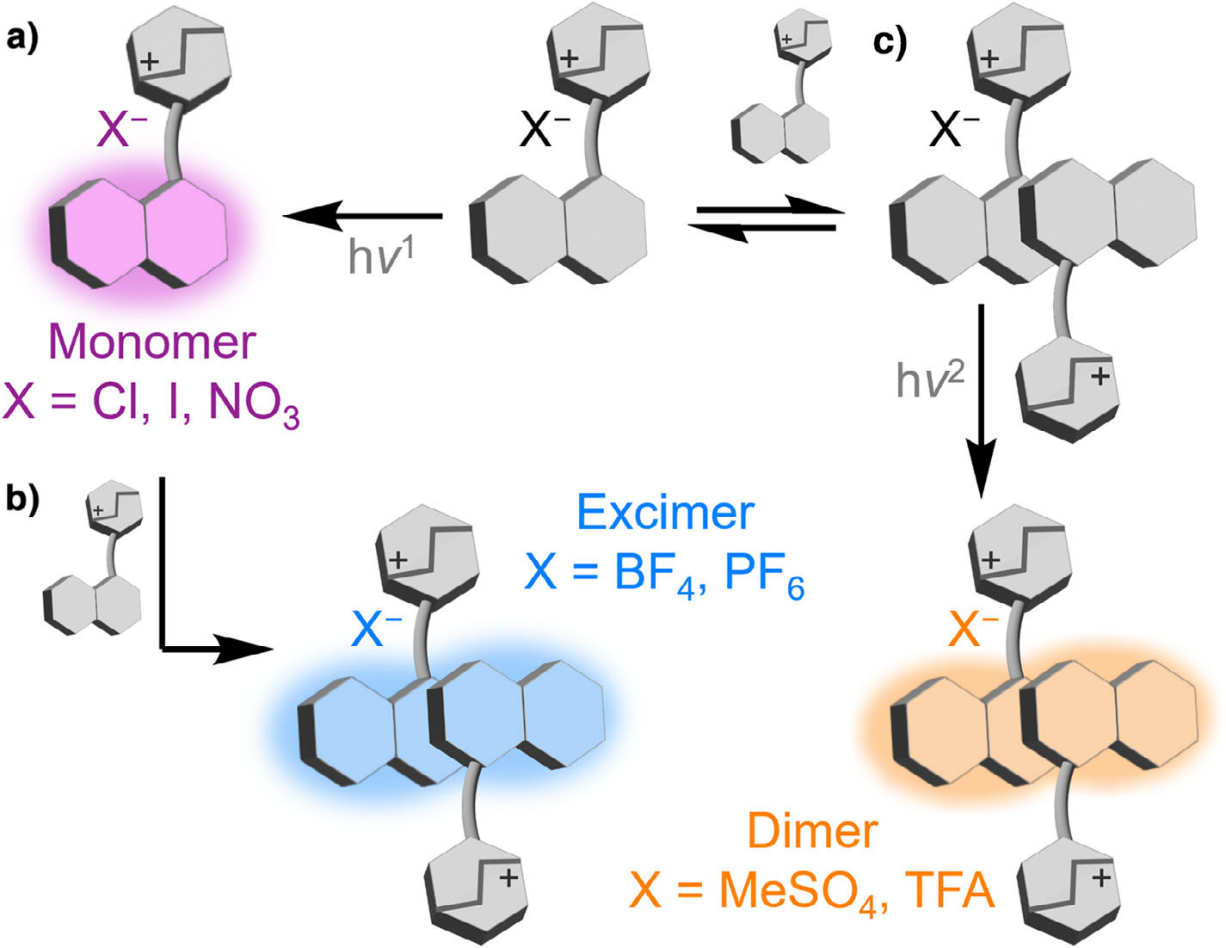
Schematic representation of this work, in which the counterion (X^−^) of a cationic luminophore (gray) dictates whether a) monomer (purple), b) excimer (blue), and/or c) dimer (orange) emission is observed in solution.

Ionic interactions, which are inherent to organic salts, can also be used to organize pairs of luminophores in space.^[^ ^39^, ^40^ ^]^ Drawing comparisons to other types of noncovalent bonding interactions, ionic interactions (i) are typically stronger and (ii) persist over longer distances, as their strength decays more gradually according to Coulomb's law. However, they (iii) are less directional^[^ ^41^ ^]^ and (iv) are often associated with “specific ion effects”,^[^ ^42^, ^43^ ^],^ e.g., changes in solvation, which make an ion's influence over assembly unpredictable. Consequently, despite organic salts (including quinines,^[^ ^44^ ^]^ rhodamines,^[^ ^45^ ^]^ pyranines,^[^ ^46^ ^]^ xanthenes,^[^ ^47^ ^]^ and so forth) being pervasive as fluorescent dyes, the effects that changing nonemissive counterions can have on the ionic interactions and their resulting aggregation and photophysical properties have received limited attention.^[^ ^48^, ^49^, ^50^ ^]^ Here, we show (Figure 1) that the noncovalent interactions between an organic ionic luminophore and its nonemissive counterion mediate its selective assembly into different dimeric species. Although certain ions favor the presence of the monomer (Figure 1a), some induce dimer formation in the excited state (Figure 1b), and others lead to ground‐state dimers (Figure 1c). The ability of the counterion to bridge two charged luminophores, arranging them close in space, therefore changes the structure of the emissive aggregate and dictates the energy of the fluorescence emission.

The permanent positive charge and photoluminescence of the *N*‑methyl quininium salts, **MeQn**·X (Figure 2), make them suitable candidates for our investigation. We have previously shown that *N*‑methylation of quinine^[^ ^44^ ^]^ and other similar luminophores^[^ ^51^, ^52^ ^]^ favors luminescence by preventing intramolecular charge transfer and subsequent nonradiative decay. Unlike protonated analogs, the methylated compounds are not in equilibrium with either the neutral or dicationic (doubly protonated) species, removing the potential complication of changes in charge state and simplifying analysis of counterion effects. Therefore, we synthesized **MeQn** ^+^ salts with a range of monovalent anions (Figure 2a). The salts are readily prepared from quinine following the *N*‐methylation and counterion exchange procedures given in the Supporting Information (Scheme S1).

**Figure 2:**
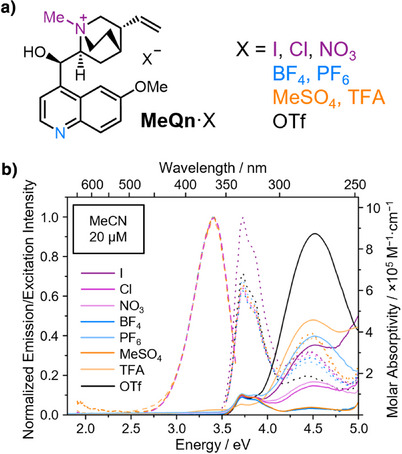
a) Structural formula of the **MeQn**·X salt series. b) Steady‐state absorption (solid lines), emission (dashed lines, *λ*_ex_ = 300 nm) and excitation (dotted lines, detected at 360 nm) spectra of **MeQn**·X salts in MeCN (20 µM).

We first measured the steady‐state UV–vis absorption, emission and excitation spectra of dilute solutions (20 µM) prepared using MeCN (Figure 2b) or water (Figure S29) as solvent. At this low concentration, there is very little variation in the spectra across the series of salts. The **MeQn** ^+^ cation absorbs below 350 nm with a peak absorption wavelength, *λ*_abs_, of 333 nm (Table 1), which corresponds to a π→π* transition centered on the quinoline ring system.^[^ ^44^ ^]^ Circular dichroism spectroscopy shows a negative Cotton effect for this signal (Figure S30). Exciting this π→π* transition (excitation wavelength, *λ*_ex_ = 300 nm) leads to fluorescence with a peak emission wavelength, *λ*_em_, of 364 nm (Table 1), arising from the locally excited state of the monomeric species.

**Table 1:** Photoluminescence data for **MeQn**·X solutions.^a)^

		*λ*_em_ / nm		*τ* / ns			
X	*λ*_abs_ / nm	*λ*_ex_ = 300 nm	*λ*_ex_ = 450 nm	Monomer	Excimer	Dimer	β^b)^
Cl	333	364	–	1.77	–	–	8.9 ± 0.1
I	333	364	–	1.64	–	–	12.1 ± 0.3
NO_3_	333	364	–	1.88	–	–	10.7 ± 0.5
BF_4_	333	364, 453	–	1.86	12.70	–	–
PF_6_	333	364, 453	–	1.86	10.03	–	7.0 ± 0.3
TFA	333, 450	–	490	–	–	2.95	–
MeSO_4_	333, 450	364	503	1.87	–	3.84	11.3 ± 0.5
OTf	333, 450	364, 453	520	1.89	4.56	4.45	9.4 ± 0.4

^a)^ 5 mM solutions of **MeQn**·X in MeCN.

^b)^ Hydrogen bond acceptor parameters reported by Hunter and co‐workers.^[^ ^53^ ^]^

At higher concentration (5 mM), however, the different **MeQn** ^+^ salts give rise to distinctive spectra (Figure 3 and Figures S31–S38). Solutions of the **MeQn**·I (Figure 3a), **MeQn**·Cl and **MeQn**·NO_3_ salts in MeCN give unchanged spectra from the low concentration measurements, retaining the characteristic *λ*_em_ of 364 nm when exciting the monomeric luminophore with *λ*_ex_ of 300 nm. In contrast, both the **MeQn**·BF_4_ (Figure 3b) and **MeQn**·PF_6_ salts give rise to a new blue emission peak at a *λ*_em_ of 453 nm. The excitation spectra (Figures 3f and S32) confirm that the blue emission arises from excitation coincident with the π→π* monomer transition at *λ*_abs_ = 333 nm. Therefore, we attribute this blue emission to an excimer, i.e., an interacting pair of **MeQn** ^+^ luminophores that assemble only in the excited state.

**Figure 3:**
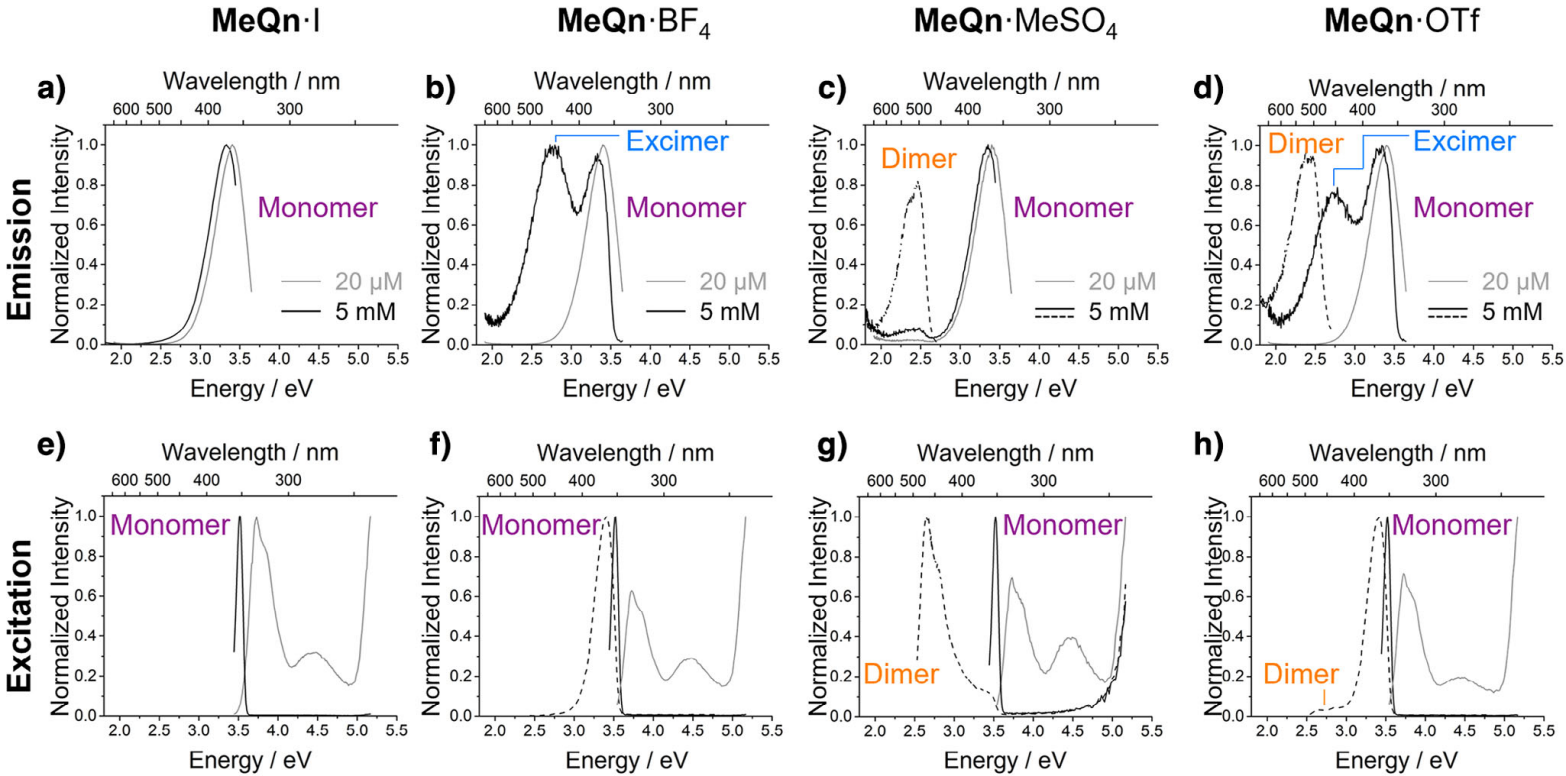
a–d) Normalized photoluminescence emission spectra of MeCN solutions. The emission of 20 µM solutions (gray solid line, *λ*_ex_ = 330 nm) is contrasted with the emission observed for 5 mM solutions (black solid line, *λ*_ex_ = 350 nm; or black dashed line, *λ*_ex_ = 450 nm). e–h) Normalized excitation spectra of MeCN solutions. The excitation of 20 µM solutions (gray solid line, detected at 365 nm) is contrasted with the excitation of 5 mM solutions (black solid line, detected at 360 nm; or black dashed line, detected at 500 nm). (a, e) **MeQn**·I, (b, f) **MeQn**·BF_4_, (c, g) **MeQn**·MeSO_4_, and (d, h) **MeQn**·OTf. Note that the band‐edge absorption is overemphasized in the excitation spectra acquired at 5 mM due to the very high optical density of luminophore.

A third outcome is observed with methyl sulfate (MeSO_4_^−^) and trifluoroacetate (TFA^−^) counterions. We observe a longer wavelength emission (*λ*_em_ ≈ 500 nm) from MeCN solutions of **MeQn**·MeSO_4_ (Figure 3c) and **MeQn**·TFA that is distinct from that of the monomer or excimer. The emission of these salts arises from excitation at *λ*_ex_ of 450 nm (Figures 3g and S37), indicating the presence of a different ground‐state luminophore. We assign this luminophore as a ground‐state dimer of **MeQn** ^+^, bridged by the TFA^−^ or MeSO_4_^−^ counterions (vide infra). The general trends observed in aqueous solution (Figures S31–S38) are similar to those stated for MeCN, although there is some variation in which counterions lead to certain species, e.g., excimer emission is also visible for the aqueous solutions of **MeQn**·I, **MeQn**·Cl, and **MeQn**·NO_3_.

All three possible emissive species of the **MeQn** ^+^ luminophore—monomer, excimer, and dimer—coexist in the presence of triflate (OTf^−^) counterions. The 5 mM MeCN solution of **MeQn**·OTf gives multichromatic fluorescence, emitting (Figure 3d) at *λ*_em_ of 364, 453 and 520 nm. Varying the temperature of the sample changes the proportion of these species (Figure S52), exemplifying the dynamic and reversible nature of the ion pairing. The ratio of monomer to excimer emission changes from ∼3:7 at +20 °C to ∼8:2 at −40 °C.

Our assignment of the monomer, excimer, and dimer species are supported by time‐correlated single photon counting (TCSPC), NMR diffusiometry measurements (Figure 4), and X‐ray crystallographic analysis (Figure 5). The TCSPC data (Table 1, Figures S42–S49) readily distinguish the three states from one another. The **MeQn** ^+^ fluorescence lifetime, *τ*, of the monomer is ∼2 ns, independent of which counterion is present, whereas the excimer emission from the BF_4_^−^, PF_6_^−^, and OTf^−^ salts at *λ*_em_ of 453 nm is longer lived (as is commonly found for excimers^[^ ^54^, ^55^ ^]^) with a *τ* ∼5–13 ns. The dimer emission of the MeSO_4_^−^, TFA^−^, and OTf^−^ salts at *λ*_em_ ∼500 nm is also distinguishable by its intermediate lifetime, which we measure to be *τ* ∼4 ns (Table 1).

**Figure 4:**
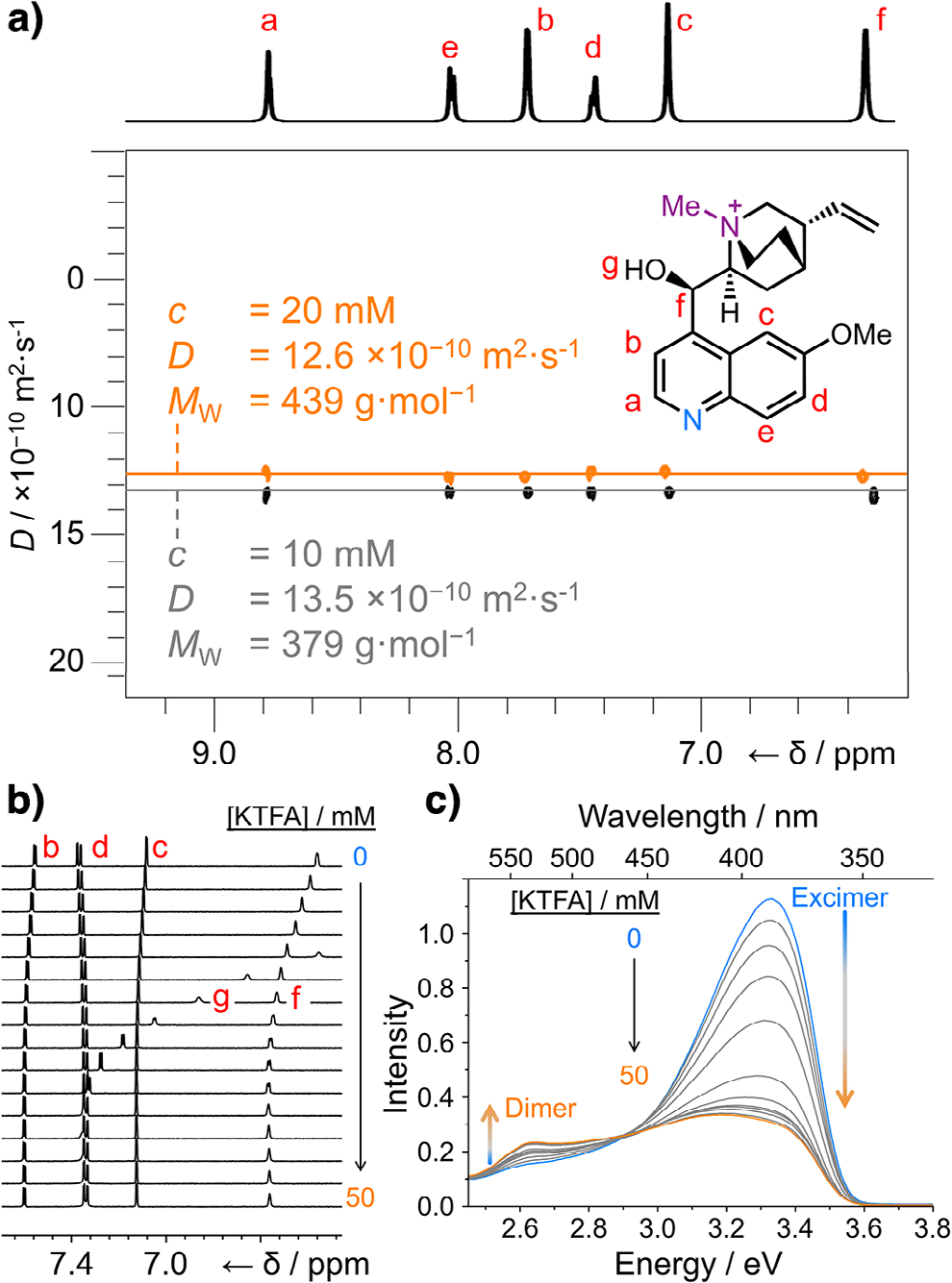
a) Partial ^1^H DOSY NMR spectra (600 MHz, 298 K, and CD_3_CN) of **MeQn**·MeSO_4_ (*c* = 10 mM, black and *c* = 20 mM, orange) showing the reduced diffusion at higher concentration indicative of ground‐state dimerization. Comparison of b) partial ^1^H NMR spectra (500 MHz, 298 K, CD_3_CN, and *c* = 5 mM) and c) excitation spectra (detected at 530 nm, MeCN, and *c* = 5 mM) of **MeQn**·PF_6_ titrated with KTFA (*c* = 0–50 mM).

**Figure 5:**
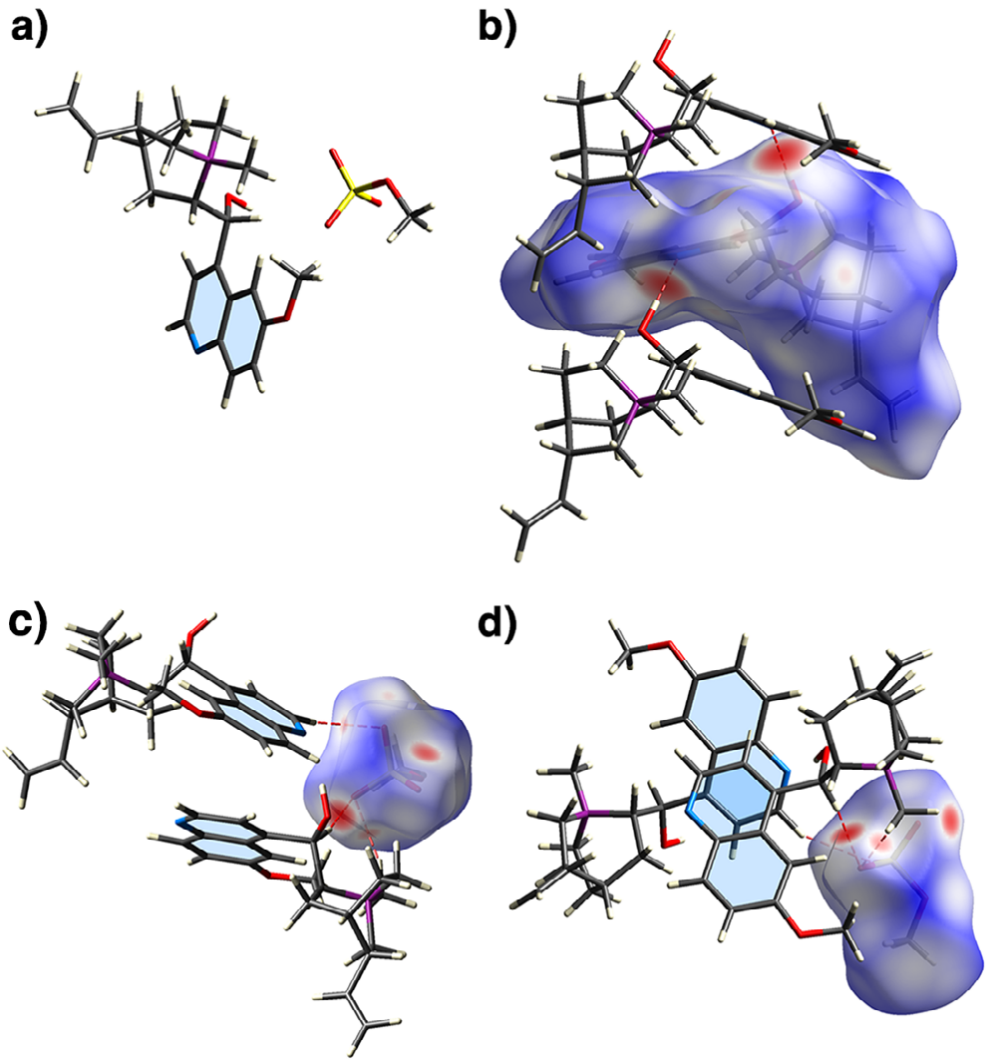
X‐ray crystallographic characterization of **MeQn**·MeSO_4_.^[^ ^55^ ^]^ a) Solid‐state structure of an ion pair. b) A chain of **MeQn** ^+^ ions linked by O─H⋯N hydrogen bonding. The middle ion is overlaid with its Hirshfeld surface. c) A section of the packing structure that shows a MeSO_4_^−^ ion, overlaid with its Hirshfeld surface, taking part in C─H⋯O hydrogen bonding to bridge two **MeQn** ^+^ ions, which d) exhibit face‐to‐face contact of their quinoline ring systems.

Diffusiometry measurements performed using ^1^H diffusion‐ordered NMR spectroscopy (DOSY) qualitatively confirm the ground‐state association of **MeQn** ^+^ fluorophores to form dimers. Comparison of the spectra acquired (Figure 4) at two different concentrations (*c* = 10 and 20 mM) of **MeQn**·MeSO_4_ in CD_3_CN show a modest decrease in the observed diffusion coefficient, *D*, at higher concentration, which is indicative of self‐ assembly of the monomers. At lower concentrations (Table S1), there is negligible difference in *D* between **MeQn**·I, **MeQn**·BF_4_, and **MeQn**·MeSO_4_, which is consistent with monomer being the predominant species. Extrapolating^[^ ^56^, ^57^ ^]^ from the *D* value observed for **MeQn**·MeSO_4_ at 20 mM to an approximate molecular weight (*M*_W_) gives values in the range of ∼440 g·mol^−1^, which is more consistent with the emergence of dimer species at equilibrium, rather than oligomerization to form extended aggregates.

We also characterized the emergence of dimer species by titrating TFA^−^ anions (added as KTFA) into a solution of **MeQn**·PF_6_. The changes in NMR chemical shifts (Figure 4b) fit to a 2:1 binding model (Figure S28) for the formation of a TFA‐bridged dimer, [**MeQn_2_** ·TFA]^+^. The association constant for the formation of an **MeQn**·TFA complex, *K*_11_, is 48 M^−1^, whereas subsequent association with a second cation to produce [**MeQn_2_** ·TFA]^+^ has an association constant, *K*_21_, of 180 M^−1^. Following the same process by fluorescence spectroscopy shows the **MeQn**·PF_6_ excimer luminescence becoming suppressed (Figure S53). Excitation spectra reveal (Figure 4c) a change in luminescence (measured at 530 nm) arising from an excimer state to a dimer as the concentration of TFA^−^ increases and [**MeQn_2_** ·TFA]^+^ is formed. In principle, therefore, these counterion‐mediated interactions could be used to detect the presence of externally added ions through a change in the aggregation state, providing the basis for a sensing mechanism.^[^ ^8^, ^9^ ^]^

X‐ray diffraction analysis of **MeQn**·MeSO_4_ single crystals^[^ ^58^ ^]^ (Figure 5) gives insights into how anions can mediate the assembly of **MeQn** ^+^. In the solid‐state packing structure (Figure 5b), O─H⋯N hydrogen bonding between the secondary alcohol and quinoline nitrogen groups links infinite chains of **MeQn** ^+^ cations. These interactions could form between **MeQn** ^+^ cations regardless of the anion, so they are evidently not sufficient on their own to cause the solution‐state dimerization. However, there are additional bridging contacts between the charged head group of the MeSO_4_^−^ anions and neighboring **MeQn** ^+^ units (Figure 5c), providing additional favorable interactions to support assembly. There is also partial face‐to‐face overlap (Figure 5d) between pairs of quinoline rings at a distance of ∼3.4 Å, which would enable the through‐space electronic communication in the dimer that is responsible for the red‐shifted absorption and emission. Indeed, irradiating a microcrystalline sample of **MeQn**·MeSO_4_ gives emission and excitation spectra (Figure S51) with the same characteristic signals as the solution‐state dimer, suggesting that this face‐to‐face pairing of the aromatic units is representative of the structure formed in solution.

We calculated the Hirshfeld surfaces^[^ ^59^ ^]^ of the cationic (Figure 5b) and anionic components (Figure 5c,d) using Crystal Explorer^[^ ^60^ ^]^ to assess the nature of the noncovalent interactions. Red patches on the anion surface highlight interatomic distances that are closer than the sum of their van der Waals radii. The sites of these close contacts match the resonances that underwent the largest changes in ^1^H NMR chemical shifts during the titration with KTFA, further confirming that the solution‐state [**MeQn_2_** ·X]^+^ dimer geometry resembles that found in the solid state. Resonance g is assigned (Figure 4b) to the alcohol group that forms an O─H⋯N hydrogen bond between two **MeQn** ^+^ units (Figure 5b), whereas resonances d and f correspond to hydrogen atoms that participate in C─H⋯O close contacts with the anion (Figure 5c).

In an attempt to elucidate the differing behavior across the series of counterions, we compared (Table 1) anion hydrogen bond acceptor parameters, β.^[^ ^53^ ^]^ However, it is evident that the propensity of the anions in the series to induce dimerization cannot simply be attributed to variations in the ionic hydrogen bond strengths alone. For example, iodide has the largest β value of 12.1, which suggests it will form the strongest ionic hydrogen bonding interactions, yet it shows no evidence of dimer or excimer fluorescence in MeCN solution (Figure 3a). Indeed, the influence of the anions extends beyond their ion–dipole (ionic hydrogen bonding) interactions with the fluorophore. Specific ion effects,^[^ ^42^, ^43^ ^]^ such as the influences of their size, shape, and charge densities on their Coulombic (ion–ion) interactions, as well as their different solvent–anion interaction strengths (e.g., through hydrogen bonding), all factor into their ability to bridge two of the cationic luminophores. We see evidence of these effects for our system – as noted above, switching from MeCN to water (Figures S31–S38) turns on excimer formation for the Cl^−^, I^−^, and NO_3_^−^ salts. Presumably, the higher dielectric constant of water alters the ion pairing to favor excimer formation. Our observations show that, although difficult to predict *a priori*, the choice of counterion cannot be underestimated because it enables rich and varied photophysical properties, even in the presence of competing interactions (such as with solvent).

Prior to our investigation, nonemissive counterions (and encapsulated ions^[^ ^61^, ^62^, ^63^ ^]^) have been used as “spacers” in the extended packing structures of solid‐state ionic materials to avoid quenching^[^ ^64^, ^65^, ^66^, ^67^, ^68^ ^]^ by preventing electronic communication between ionic luminophores.^[^ ^69^, ^70^ ^]^ Appropriate choice of counterion can favor rigidification and alter dipolar interactions^[^ ^48^ ^]^ in the solid state, inducing fluorescence^[^ ^71^, ^72^, ^73^ ^]^ or phosphorescence.^[^ ^40^, ^74^, ^75^, ^76^, ^77^, ^78^, ^79^ ^]^ Similarly, in solution, symmetry‐breaking^[^ ^48^, ^49^, ^80^ ^]^ or conformational and electronic changes caused by an ion binding to a monomeric luminophore (particularly organometallics^[^ ^81^ ^]^) have been used to modify photoinduced electron transfer^[^ ^82^ ^]^ or energy transfer processes.^[^ ^83^, ^84^ ^]^ Our investigation has shown that specific ion effects in solution^[^ ^42^, ^43^ ^]^ are not only capable of modulating quenching or energy transfer processes, they can also induce selective assembly of discrete aggregates with distinct photophysical properties. Rather than acting as spacers to prevent electronic communication or change solubility,^[^ ^50^, ^85^ ^]^ the counterions act as a “glue” to favor specific types of electronically coupled aggregates. These observations provide a basis to design ion sensors that operate through luminophore assembly, as well as providing convenient systems to probe specific ion effects across a range of solvents and counterions.

## Supporting Information

General experimental details and procedures, X‐ray crystallographic details, electronic spectra, and NMR spectra. The authors have cited additional references within the Supporting Information.^[^ ^86^, ^87^, ^88^, ^89^, ^90^, ^91^, ^92^ ^]^

## Conflict of Interests

The authors declare no conflict of interest.

## Supporting information



Supporting Information

## Data Availability

The data that support the findings of this study are available in the Supporting Information of this article.
