# Supplementary material for: Counterion‐Mediated Luminophore Dimerization
Source: Angew Chem Int Ed Engl. 2025 Jun 25;64(34):e202505433. doi: 10.1002/anie.202505433 (PMC12363632; doi:10.1002/anie.202505433)
Supplement: Supplementary file 1 — Supporting Information [file ANIE-64-e202505433-s001.docx]

**Counterion-Mediated Luminophore Dimerization**

Ash G. Carter,^a,b^ Promeet K. Saha,^a,b^ Antara Sikder,^a^ Juan A. Aguilar,^b^ Andrew P. Monkman,^c^ Alyssa-Jennifer Avestro,^a^ Marc K. Etherington,^d^ Paul R. McGonigal^a,e^

^a^Department of Chemistry, University of York, Heslington, York, YO10 5DD, UK. ^b^Department of Chemistry, Durham University, Lower Mountjoy, Stockton Road, Durham, DH1 3LE, UK. ^c^Department of Ph­­ysics, Durham University, Lower Mountjoy, Stockton Road, Durham, DH1 3LE, UK. ^d^Department of Mathematics, Physics and Electrical Engineering, Northumbria University, Ellison Place, Newcastle upon Tyne, NE1 8ST, UK. ^e^Department of Chemistry, University of Oxford, Mansfield Road, Oxford, OX1 3TA, UK.

**Table of Contents**

1. General Methods S1

2. Synthetic Procedures S4

3. ^1^H, ^13^C{^1^H} and ^19^F NMR Spectroscopic Characterisation of Synthesised Compounds S11

4. Photophysical Data S38

5. X-Ray Crystallographic Analysis S50

6. Additional TCSPC Data S51

7. Additional Photophysical Data S57

Supporting Information

**1. General Methods**

**Materials**: All reagents were purchased from commercial suppliers (Sigma-Aldrich, Acros Organics, or Alfa Aesar) and used without further purification.

**Instrumentation and Analytical Techniques**: Analytical thin-layer chromatography (TLC) was performed on neutral aluminium-sheet silica gel plates and visualised under UV irradiation (254 nm). Column chromatography was performed using a Teledyne Isco CombiFlash Rf 200 system. Nuclear magnetic resonance (NMR) spectra were recorded using a Bruker Advance (III)-400 (^1^H 400.130 MHz and ^13^C 100.613 MHz), Varian Inova-500 (^1^H 500.130 MHz and ^13^C 125.758 MHz), Varian VNMRS-600 (^1^H 600.130 MHz and ^13^C 150.903 MHz) or a Varian VNMRS-700 (^1^H 700.130 MHz and ^13^C 176.048 MHz) spectrometers, at a constant temperature of 298 K unless otherwise stated. Chemical shifts (*δ*) are reported in parts per million (ppm) relative to the signals corresponding to residual non-deuterated solvents [DMSO-*d*_6_: *δ* = 2.50 or 39.52. CD_3_OD: *δ* = 3.31 or 49.00]. Coupling constants (*J*) are reported in Hertz (Hz). ^13^C NMR Experiments were proton-decoupled, whereas ^19^F NMR experiments are coupled and referenced to an internal standard, hexafluorobenzene (HFB, δ = 164.9 ppm). Assignments of ^1^H and ^13^C NMR signals were accomplished by two-dimensional NMR spectroscopy (^1^H–^1^H COSY, ^1^H–^1^H NOESY, ^1^H–^13^C HSQC-multiplicity edited, ^1^H–^13^C HMBC). NMR spectra were processed using MestReNova version 14 or version 15. Data are reported as follows: chemical shift; multiplicity; coupling constants; integral and assignment. A 600 MHz Varian spectrometer equipped with an Agilent OneNMR Probe able to deliver a maximum pulsed field gradient of 62 G cm^-1^ was used to acquire ^1^H Diffusion-Ordered SpectroscopY (DOSY) experiments using a convection compensated pulse sequence based on a double stimulated echo. The pulse sequence, Dbppstee_cc, is part of the VNMJ 4.2 pulse sequence library.^[86,87]^ Twenty gradient amplitudes ranging from 1.95 to 29.25 G cm^-1^ spaced in equal steps of gradient squared were used. Thirty-two transients were collected. Thirty-two steady-state transients were used. The number of complex data points was 21,406 covering 6.3 kHz were collected. The diffusion-encoding pulsed gradient duration was 2.0 ms. The diffusion time was 200 ms. The gradient stabilisation delay was 2.0 ms. The repetition time was 6.4 s, of which 3.4 s comprised the acquisition time. The unbalancing factor was 0.15. The results were analysed with VNMRJ 4.2 using mono-exponential fittings. The effects of non-uniform field gradients were accounted for using methods developed by G.A. Morris, as implemented in VNMRJ 4.2.^[88]^ Molecular weights were estimated by DOSY NMR using the Stokes–Einstein Gierer–Wirtz Estimation method.^[56,57]^ High-resolution electrospray (HR-ESI) mass spectra were measured using a Waters LCT Premier XE high resolution, accurate mass UPLC ES MS. Elemental analysis data was taken using an Exeter Analytical CE-440 elemental analyser, with analysis parameters of horizontal furnace, with static combustion in pure oxygen and calibrated with acetanilide. Samples for elemental analysis were weighed out using Sartorius SE2 microbalance. Melting points were recorded using a Gallenkamp (Sanyo) apparatus and are uncorrected. Samples for solution-state optical measurements were prepared by dilution of concentrated stock solutions under ambient conditions and measured in 10-mm path length quartz cuvettes. UV-Vis-NIR absorbance spectra of solution samples were recorded using an Agilent Technologies Cary Series UV-vis-NIR spectrophotometer at room temperature. Steady-state photoluminescence of solutions were measured using a Jobin Yvon Fluoromax or Fluorolog with machine-specific calibration curves. Low-temperature spectra were acquired using a Janis VNF-100 and Lakeshore model 322 temperature controller to cool the sample. Photoluminescence quantum yields (PLQYs) of the compounds were measured by the absolute method using an integrating sphere in conjunction with the HORIBA Fluorolog-QM with neutral density filters to correct for high intensity excitation. The PLQYs were calculated using the mathematical formula included within the HORIBA FelixFl software. Solid-state luminescence data was acquired for a free-standing microcrystalline powder of **MeQn**·SO_4_ on a glass slide using a Jobin Yvon Fluorolog (excitation) or Hitachi F-4500 (emission) spectrophotometer. Time-correlated single photon counting (TCSPC) measurements were taken on a HORIBA DeltaFlex TCSPC/MCS Fluorescence Lifetime System in combination with a DeltaDiode laser emitting at 300 nm or 415 nm. The X-ray single crystal data for **MeQn**·MeSO_4_ have been collected at temperature 120.0(2)K using MoKα radiation (λ =0.71073Å) on a Bruker D8Venture (Photon100 CMOS detector, IμS-microsource, focusing mirrors) 3-circle diffractometer equipped with a Cryostream (Oxford Cryosystems) open-flow nitrogen cryostat. The structure was solved by direct method and refined by full-matrix least squares on F^2^ for all data using Olex2^[89]^ and SHELXTL^[90]^ software. All non-hydrogen atoms were refined in anisotropic approximation, most of the hydrogen atoms were located in in the residual maps and refined isotropically. Hydrogen atoms of Me-groups were placed in calculated positions and refined in riding mode. The absolute configuration of the compound has been established by measurements of anomalous dispersion effects (Flack parameter -0.05(2), Hooft parameter -0.04(2)). Crystal data and parameters of refinement are listed in Table S2. Circular dichroism spectra were recorded at 20.0 °C in a 10-mm quartz Spectrasil cuvette (QS grade, Hellma Analytics) using a JASCO J-1500 spectropolarimeter configured with a PMT detector (193–950 nm nominal range and 150 W Xe lamp) under an active flow (2 mL min^-1^) of nitrogen gas.

**2. Synthetic Procedures**

**
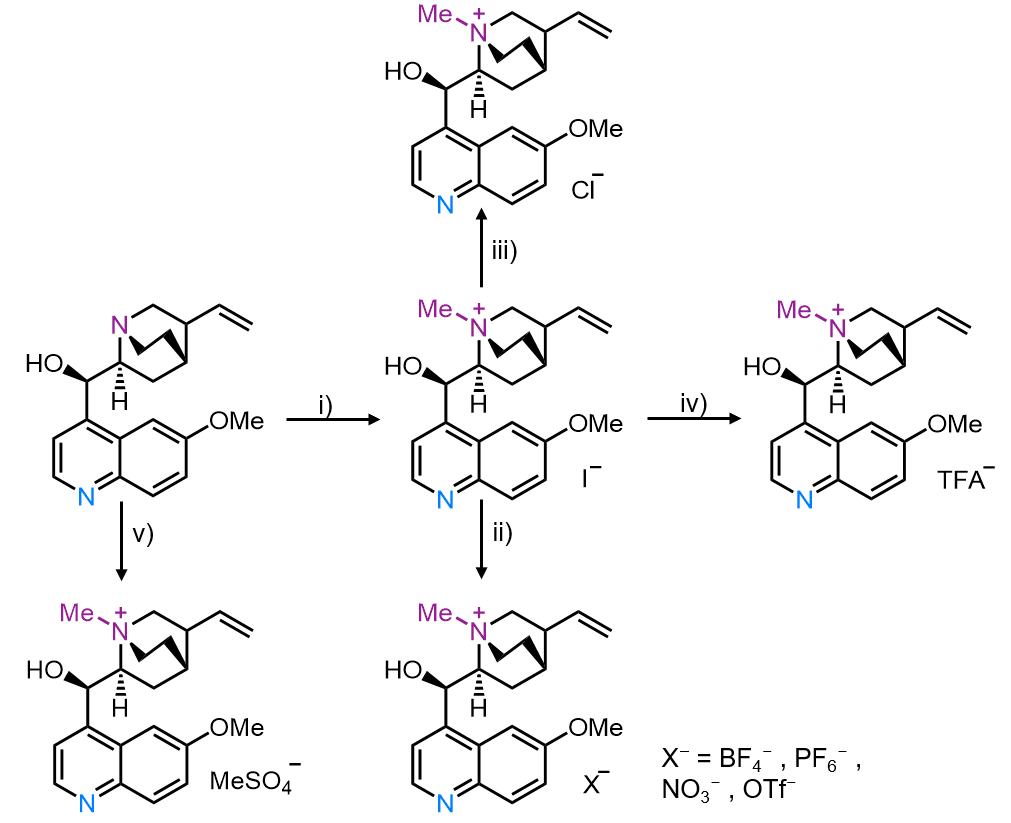
**

**Scheme S1.** Synthetic routes to **MeQn**·X. Reagents and conditions: (i) MeI / rt / 3 d (67%); (ii) AgX / MeCN / 60 °C / 10 min, X^−^ = BF_4_^−^ (86%), PF_6_^−^ (85%), NO_3_^−^ (97%), OTf^−^ (90%); (iii) chloride ion exchange resin in H_2_O/MeOH 4:1 (quant.); (iv) **MeQn**·I / reverse phase chromatography., H_2_O−MeOH with 0.1% TFA (84%); (v) Me_2_SO_4_ / MeCN / 100 °C / 8 h (64%).

**MeQn·X salts**

**MeQn·**I: Quinine (250 mg, 0.77 mmol) was added to an oven-dried microwave vial and sealed. MeI (2.5 mL) was added, and the mixture was sonicated at rt for 20 min. The reaction mixture was stirred for 3 d at rt. The mixture was filtered, and the precipitate was washed with CH_2_Cl_2_ (2 × 5 mL), then dried under high vacuum. The resulting solid was recrystallized from MeCN to yield the iodide salt of title compound, **MeQn·**I, as a crystalline solid (239 mg, 0.513 mmol, 67%). **M.P.** 232–235 °C. **Elemental Analysis** Calculated for C_21_H_27_N_2_O_2_I: C, 54.04; H, 5.79; N, 6.00. Found: C, 53.08; H, 5.77; N, 5.75. **^1^H NMR** (599 MHz, DMSO-*d_6_*) δ 8.79 (d, *J* = 4.5 Hz, 1H, H_10_), 8.00 (d, *J* = 9.1 Hz, 1H, H_2_), 7.70 (d, *J* = 4.5 Hz, 1H, H_9_), 7.48 (dd, *J* = 9.2, 2.6 Hz, 1H, H_3_), 7.21 (d, *J* = 2.7 Hz, 1H, H_6_), 6.53 (d, *J* = 3.5 Hz, 1H, H_21_), 6.22 (d, *J* = 3.6 Hz, 1H, H_11_), 5.75 (ddd, *J* = 17.3, 10.5, 6.8 Hz, 1H, H_17_), 5.13 (dt, *J* = 17.2, 1.4 Hz, 1H, H_18_), 5.01 (dt, *J* = 10.5, 1.3 Hz, 1H, H_18_), 4.12–4.03 (m, 1H, H_20_), 4.00 (s, 3H, H_5_), 3.74–3.68 (m, 1H, H_12_), 3.69–3.60 (m, 2H, H_16_), 3.47–3.39 (m, 1H, H_20_), 3.39 (s, 3H, H_22_), 2.83–2.79 (m, 1H, H_15_), 2.19–2.10 (m, 2H, H_13+19_), 2.10–2.02 (m, 1H, H_14_), 1.96–1.89 (m, 1H, H_19_), 1.38–1.31 (m, 1H, H_13_). **^13^C****{^1^H} NMR** (151 MHz, DMSO-*d_6_*) δ 157.8 (C_4_), 147.9 (C_10_), 144.3 (C_8_), 144.1 (C_1_), 138.5 (C_17_), 131.9 (C_2_), 125.6 (C_7_), 122.0 (C_3_), 120.4 (C_9_), 117.0 (C_18_), 102.0 (C_6_), 67.3 (C_12_), 64.3 (C_11_), 64.3 (C_16_), 56.0 (C_5_), 54.7 (C_20_), 49.2 (C_22_), 38.0 (C_15_), 26.3 (C_14_), 25.1 (C_19_), 19.8 (C_13_). **HRMS-ESI***m/z* = 339.2085 [M−I]^+^, calculated for C_21_H_27_N_2_O_2_: 339.2073. Spectroscopic data are consistent with those previously published.^[44]^

**MeQn·**BF_4_: **MeQn·**I (50 mg, 0.107 mmol) was dissolved in MeCN (4 mL) and heated to 60 °C. A solution of AgBF_4_ (21.0 mg, 0.107 mmol) in MeCN (1 mL) was added dropwise, and the mixture was allowed to stir at 60 °C for 10 min. Upon cooling to rt, the reaction mixture was filtered, and the filtrate was evaporated to dryness to give the title compound as a pale yellow solid (54 mg, 0.127 mmol, 86%). **M.P.** 185–187 °C. **Elemental Analysis** Calculated for C_21_H_27_N_2_O_2_BF_4_.H­_2_O: C, 56.72; H, 6.08; N, 6.30. Found: C, 57.64; H, 6.54; N, 6.42. **^1^H NMR** (700 MHz, DMSO-*d_6_*) δ 8.79 (d, *J* = 4.5 Hz, 1H, H_10_), 8.00 (d, *J* = 9.2 Hz, 1H, H_2_), 7.70 (d, *J* = 4.9 Hz, 1H, H_9_), 7.48 (dd, *J* = 9.2, 2.7 Hz, 1H, H_3_), 7.21 (d, *J* = 2.7 Hz, 1H, H_6_), 6.54 (d, *J* = 2.7 Hz, 1H, H_21_), 6.21 (d, *J* = 3.2 Hz, 1H, H_11_), 5.75 (ddd, *J* = 17.3, 10.5, 6.8 Hz, 1H, H_17_), 5.13–5.01 (m, 2H, H_18_), 4.07 (m, 1H, H_20_), 4.00 (s, 3H, H_5_), 3.74–3.60 (m, 3H, H_16+12_), 3.45–3.35 (m, 4H, H_20+22_), 2.85–2.76 (m, 1H, H_15_), 2.20–2.10 (m, 2H, H_13+19_), 2.06–2.01 (m, 1H, H_14_), 1.97–1.87 (m, 1H, H_19_), 1.38–1.31 (m, 1H, H_13_). **^13^C{^1^H} NMR** (151 MHz, DMSO-*d_6_*) δ 157.8 (C_4_), 147.9 (C_10_), 144.3 (C_8_), 144.1 (C_1_), 138.5 (C_17_), 131.9 (C_2_), 125.6 (C_7_), 121.5 (C_3_), 120.4 (C_9_), 117.0 (C_18_), 102.0 (C_6_), 67.2 (C_12_), 64.4 (C_11_), 64.4 (C_16_), 56.0 (C_5_), 54.7 (C_20_), 49.2 (C_22_), 38.0 (C_15_), 26.3 (C_14_), 25.1 (C_19_), 19.8 (C_13_). **^19^F NMR** (376 MHz, CD_3_OD) δ −150.48 (m, ^10^B), −150.54 (m, ^11^B). **HRMS-ESI***m/z* = 339.2079 [M−BF_4_]^+^, calculated for C_21_H_27_N_2_O_2_: 339.2073. Spectroscopic data consistent with those previously published.^[44]^

**MeQn·**PF_6_: **MeQn·**I (200 mg, 0.428 mmol) was dissolved in MeCN (15 mL) and a solution of AgPF_6_ (108 mg, 0.428 mmol) in MeCN (2 mL) was added dropwise. The mixture was stirred at 60 °C for 1 h. Upon cooling to rt, the reaction mixture was filtered, and the filtrate was evaporated to dryness to yield the title compound as a colorless solid (177 mg, 0.365 mmol, 85%). **M.P.** 214–218 °C. **Elemental Analysis** Calculated for C_21_H_27_N_2_O_2_PF_6_: C, 52.02; H, 5.57; N, 5.78. Found: C, 51.54; H, 5.79; N, 5.87. **^1^H NMR** (400 MHz, DMSO-*d_6_*) δ 8.79 (d, *J* = 4.5 Hz, 1H, H_10_), 8.00 (d, *J* = 9.2 Hz, 1H, H_2_), 7.70 (d, *J* = 4.5 Hz, 1H, H_9_), 7.48 (dd, *J* = 9.2, 2.6 Hz, 1H, H_3_), 7.21 (d, *J* = 2.7 Hz, 1H, H_6_), 6.54 (d, *J* = 3.7 Hz, 1H, H_21_), 6.22 (d, *J* = 3.9 Hz, 1H, H_11_), 5.75 (ddd, *J* = 17.3, 10.5, 6.8 Hz, 1H, H_17_), 5.17–4.97 (m, 2H, H_18_), 4.13–3.96 (m, 1H, H_20_), 4.00 (s, 3H, H_5_), 3.74–3.59 (m, 2H, H_16_), 3.47–3.26 (m, 4H, H_20+22_), 2.81 (d, *J* = 8.4 Hz, 1H, H_15_), 2.18–2.07 (m, 2H, H_13+19_), 2.07 (s, 1H, H_12_), 2.07–2.01 (m, 1H, H_14_), 1.97–1.90 (m, 1H, H_19_), 1.39–1.29 (m, 1H, H_13_). **^13^C{^1^H} NMR** (101 MHz, DMSO‑*d_6_*) δ 157.4 (C_4_), 147.5 (C_10_), 143.9 (C_8_), 143.7 (C_1_), 138.0 (C_17_), 131.5 (C_2_), 125.2 (C_7_), 121.6 (C_3_), 120.0 (C_9_), 116.6 (C_18_), 101.6 (C_6_), 66.8 (C_12_), 63.9 (C_11_), 63.9 (C_16_), 55.5 (C_5_), 54.2 (C_20_), 48.8 (C_22_), 37.6 (C_15_), 25.9 (C_14_), 24.6 (C_19_), 19.3 (C_13_). **^19^F NMR** (376 MHz, DMSO-*d_6_*) δ −72.45 (d, *J*_FP_ = 711.1 Hz). **HRMS-ESI***m/z* = 339.2075 [M−PF_6_]^+^, calculated for C_21_H_27_N_2_O_2_: 339.2073.

**MeQn·**OTf: **MeQn·**I (200 mg, 0.428 mmol) was dissolved in MeCN (15 mL) and a solution of AgOTf (110 mg, 0.428 mmol) in MeCN (2 mL) was added dropwise. The mixture was stirred at 60 °C for 1 h. Upon cooling to rt, the reaction mixture was filtered, and the filtrate was evaporated to dryness to yield the title compound as a colorless solid (189 mg, 0. 387 mmol, 90%). **M.P.** 197–199 °C. **Elemental Analysis** Calculated for C_22_H_27_N_2_O_5_SF_3_.H_2_O: C, 52.12; H, 5.33; N, 5.53. Found: C, 52.19; H, 5.56; N, 5.31. **^1^H NMR** (400 MHz, DMSO-*d_6_*) δ 8.79 (d, *J* = 4.5 Hz, 1H, H_10_), 8.00 (d, *J* = 9.2 Hz, 1H, H_2_), 7.70 (d, *J* = 4.5 Hz, 1H, H_9_), 7.48 (dd, *J* = 9.2, 2.6 Hz, 1H, H_3_), 7.21 (d, *J* = 2.7 Hz, 1H, H_6_), 6.55 (dd, *J* = 3.8, 0.9 Hz, 1H, H_21_), 6.22 (d, *J* = 3.8 Hz, 1H, H_11_), 5.75 (ddd, *J* = 17.3, 10.4, 6.8 Hz, 1H, H_17_), 5.17–4.97 (m, 2H, H_18_), 4.13–4.02 (m, 1H, H_20_), 4.00 (s, 3H, H_5_), 3.75–3.59 (m, 3H, H_16+12_), 3.46–3.36 (m, 4H, H_20+22_), 2.84–2.77 (m, 1H, H_15_), 2.22–2.08 (m, 2H, H_13+19_), 2.11–2.00 (m, 1H, H_14_), 1.97–1.87 (m, 1H, H_19_), 1.40–1.28 (m, 1H, H_13_). **^13^C{^1^H} NMR** (101 MHz, DMSO-*d_6_*) δ 157.4 (C_4_), 147.5 (C_10_), 143.9 (C_8_), 143.6 (C_1_), 138.0 (C_17_), 131.5 (C_2_), 125.2 (C_7_), 121.6 (C_3_), 120.7 (q, *J*_CF_ = 322.6 Hz, SO_3_CF_3_), 120.0 (C_9_), 116.6 (C_18_), 101.6 (C_6_), 66.8 (C_12_), 63.9 (C_11_), 63.9 (C_16_), 55.5 (C_5_), 54.2 (C_20_), 48.8 (C_22_), 37.6 (C_15_), 25.9 (C_14_), 24.6 (C_19_), 19.3 (C_13_). **^19^F NMR** (376 MHz, DMSO-*d_6_*) δ −80.04. **HRMS-ESI***m/z* = 339.2085 [M−OTf]^+^, calculated for C_21_H_27_N_2_O_2_: 339.2073.

**MeQn·**NO_3_: **MeQn·**I (200 mg, 0.428 mmol) was dissolved in MeCN (15 mL) and a solution of AgNO_3_ (72.7 mg, 0.428 mmol) in MeCN (2 mL) was added dropwise. The mixture was stirred at 60 °C for 1 h. Upon cooling to rt, the reaction mixture was filtered, and the filtrate was evaporated to dryness to yield the title compound as a colorless solid (167 mg, 0. 417 mmol, 97%). **M.P.** 207–210 °C. **Elemental Analysis** Calculated for C_21_H_27_N_3_O_5_: C, 62.77; H, 6.73; N, 10.46. Found: C, 61.18; H, 6.77; N, 11.35. **^1^H NMR** (400 MHz, DMSO-*d_6_*) δ 8.79 (d, *J* = 4.5 Hz, 1H, H_10_), 8.00 (d, *J* = 9.2 Hz, 1H, H_2_), 7.70 (d, *J* = 4.5 Hz, 1H, H_9_), 7.48 (dd, *J* = 9.2, 2.6 Hz, 1H, H_3_), 7.21 (d, *J* = 2.7 Hz, 1H, H_6_), 6.57 (d, *J* = 3.7 Hz, 1H, H_21_), 6.22 (d, *J* = 3.8 Hz, 1H, H_11_), 5.75 (ddd, *J* = 17.3, 10.4, 6.8 Hz, 1H, H_17_), 5.17–4.97 (m, 2H, H_18_), 4.13–4.02 (m, 1H, H_20_), 4.00 (s, 3H, H_5_), 3.75–3.59 (m, 3H, H_16+12_), 3.47–3.37 (m, 4H, H_20+22_), 2.84–2.77 (m, 1H, H_15_), 2.21–2.06 (m, 2H, H_13+19_), 2.06–2.02 (m, 1H, H_14_), 1.97–1.90 (m, 1H, H_19_), 1.39–1.29 (m, 1H, H_13_). **^13^C{^1^H} NMR** (101 MHz, DMSO-*d_6_*) δ 157.4 (C_4_), 147.5 (C_10_), 143.9 (C_8_), 143.6 (C_1_), 138.1 (C_17_), 131.5 (C_2_), 125.2 (C_7_), 121.6 (C_3_), 120.0 (C_9_), 116.6 (C_18_), 101.5 (C_6_), 66.8 (C_12_), 63.9 (C_11_), 63.9 (C_16_), 55.5 (C_5_), 54.2 (C_20_), 48.8 (C_22_), 37.6 (C_15_), 25.9 (C_14_), 24.6 (C_19_), 19.3 (C_13_). **HRMS-ESI***m/z* = 339.2084 [M−NO_3_]^+^, calculated for C_21_H_27_N_2_O_2_: 339.2073.

**MeQn·**Cl: **MeQn·**I (200 mg, 0.428 mmol) was dissolved in H_2_O (10 mL) and MeOH (10 mL) and the resulting solution was passed through an ion exchange column (Dowex® 1X8 chloride form, 20 g, H_2_O:MeOH 4:1 as the eluent) to yield the title compound as a colorless solid (175 mg, 0.467 mmol, quant.). **M.P.** 183–186 °C. **Elemental Analysis** Calculated for C_21_H_27_N_2_O_2_Cl.2H_2_O: C, 61.32; H, 6.57; N, 6.81. Found: C, 61.27; H, 7.43; N, 6.77. **^1^H NMR** (400 MHz, DMSO-*d_6_*) δ 8.78 (d, *J* = 4.5 Hz, 1H, H_10_), 8.00 (d, *J* = 9.2 Hz, 1H, H_2_), 7.71 (d, *J* = 4.5 Hz, 1H, H_9_), 7.47 (dd, *J* = 9.2, 2.6 Hz, 1H, H_3_), 7.22 (d, *J* = 2.7 Hz, 1H, H_6_), 6.94 (d, *J* = 4.7 Hz, 1H, H_21_), 6.23 (d, *J* = 4.7 Hz, 1H, H_11_), 5.75 (ddd, *J* = 17.3, 10.5, 6.8 Hz, 1H, H_17_), 5.18–4.97 (m, 2H, H_18_), 4.28–4.16 (m, 1H, H_20_), 4.01 (s, 3H, H_5_), 3.78–3.60 (m, 3H, H_16+12_), 3.44 (s, 3H, H_22_), 3.16 (s, 1H, H_20_), 2.85–2.77 (m, 1H, H_15_), 2.21–2.06 (m, 2H, H_13+19_), 2.06–1.99 (m, 1H, H_14_), 1.96–1.85 (m, 1H, H_19_), 1.41–1.29 (m, 1H, H_13_). **^13^C{^1^H} NMR** (101 MHz, DMSO-*d_6_*) δ 157.4 (C_4_), 147.4 (C_10_), 144.3 (C_8_), 143.5 (C_1_), 138.1 (C_17_), 131.3 (C_2_), 125.3 (C_7_), 121.7 (C_3_), 120.1 (C_9_), 116.5 (C_18_), 101.6 (C_6_), 66.9 (C_12_), 63.9 (C_11_), 63.6 (C_16_), 55.6 (C_5_), 54.0 (C_20_), 48.7 (C_22_), 37.6 (C_15_), 25.9 (C_14_), 24.6 (C_19_), 19.6 (C_13_). **HRMS-ESI***m/z* = 339.2066 [M−Cl]^+^, calculated for C_21_H_27_N_2_O_2_: 339.2073.

**MeQn·**TFA**: MeQn·**I (200 mg, 0.428 mmol) was passed through a reverse phase chromatography column (Teledyne Isco CombiFlash Rf+ system, 15.5 g C18, H_2_O−CH_3_OH, 0–100 % CH_3_OH gradient elution, 0.1% trifluoroacetic acid in the eluent) to yield the title compound as an orange solid (163 mg, 0.359 mmol, 84%). **M.P.** 192–195 °C. **^1^H NMR** (599 MHz, CD_3_OD) δ 8.75 (d, *J* = 4.6 Hz, 1H, H_10_), 8.02 (d, *J* = 9.2 Hz, 1H, H_2_), 7.85 (d, *J* = 4.6 Hz, 1H, H_9_), 7.51 (dd, *J* = 9.2, 2.6 Hz, 1H, H_3_), 7.26 (d, *J* = 2.7 Hz, 1H, H_6_), 6.33 (s, 1H, H_11_), 5.74 (ddd, *J* = 17.2, 10.5, 6.8 Hz, 1H, H_17_), 5.19–5.03 (m, 2H, H_18_), 4.44–4.35 (m, 1H, H_20_), 4.05 (s, 3H, H_5_), 3.78 (dd, *J* = 13.0, 10.6 Hz, 1H, H_12_), 3.69–3.62 (m, 2H, H_16_), 3.51–3.42 (m, 4H, H_20+22_), 2.93–2.90 (m, 1H, H_15_), 2.36–2.26 (m, 2H, H_13+19_), 2.17–2.10 (m, 1H, H_14_), 2.10–2.01 (m, 1H, H_19_), 1.50–1.42 (m, 1H, H_13_). **^19^F NMR** (376 MHz, DMSO-*d_6_*) δ −76.52. **HRMS-ESI**m/z = 339.2081 [M−TFA]^+^, calculated for C_21_H_27_N_2_O_2_: 339.2073.

**MeQn·**MeSO_4_: Dimethyl sulfate (194 mg, 1.54 mmol) and MeCN (4.5 mL) were added to a microwave vial. Quinine (500 mg, 1.54 mmol) was added and the vial was sealed. The resultant mixture was heated to 100 °C for 8 h in microwave reactor. The reaction mixture was evaporated to dryness, and the resulting solid was washed with MeCN (2 mL), then dried under vacuum to yield the title compound as a colorless solid (417 mg, 0.93 mmol, 64%). **M.P.** 191–195 °C. **Elemental Analysis** Calculated for C_22_H_30_N_2_O_6_S: C, 58.60; H, 6.66; N, 6.21. Found: C, 57.66; H, 6.59; N, 5.86. **^1^H NMR** (400 MHz, DMSO-*d_6_*) δ 8.79 (d, *J* = 4.5 Hz, 1H, H_10_), 8.00 (d, *J* = 9.2 Hz, 1H, H_2_), 7.70 (d, *J* = 4.5 Hz, 1H, H_9_), 7.48 (dd, *J* = 9.2, 2.6 Hz, 1H, H_3_), 7.21 (d, *J* = 2.7 Hz, 1H, H_6_), 6.55 (d, *J* = 3.5 Hz, 1H, H_21_), 6.22 (d, *J* = 3.8 Hz, 1H, H_11_), 5.75 (ddd, *J* = 17.3, 10.5, 6.8 Hz, 1H, H_17_), 5.17–4.97 (m, 2H, H_18_), 4.13–4.02 (m, 1H, H_20_), 4.00 (s, 3H, H_5_), 3.75–3.59 (m, 3H, H_16+12_), 3.48–3.38 (m, 4H, H_20+22_), 3.37 (s, 3H, CH_3_SO_4_), 2.84–2.77 (m, 1H, H_15_), 2.21–2.09 (m, 2H, H_13+19_), 2.07–2.01 (m, 1H, H_14_), 1.97–1.87 (m, 1H, H_19_), 1.39–1.29 (m, 1H, H_13_). **^13^C{^1^H} NMR** (101 MHz, DMSO-*d_6_*) δ 157.4 (C_4_), 147.5 (C_10_), 143.9 (C_8_), 143.6 (C_1_), 138.1 (C_17_), 131.5 (C_2_), 125.2 (C_7_), 121.6 (C_3_), 120.0 (C_9_), 116.6 (C_18_), 101.6 (C_6_), 66.8 (C_12_), 63.9 (C_11_), 63.9 (C_16_), 55.5 (C_5_), 54.2 (C_20_), 52.8 (CH_3_SO_4_), 48.7 (C_22_), 37.6 (C_15_), 25.9 (C_14_), 24.6 (C_19_), 19.3 (C_13_). **HRMS-ESI** *m/z* = 339.2078 [M−MeSO_4_]^+^, calculated for C_21_H_27_N_2_O_2_: 339.2073.

### **3.1 ^1^H,** **^13^C and ^19^F NMR Spectroscopic Characterisation of Synthesised Compounds**


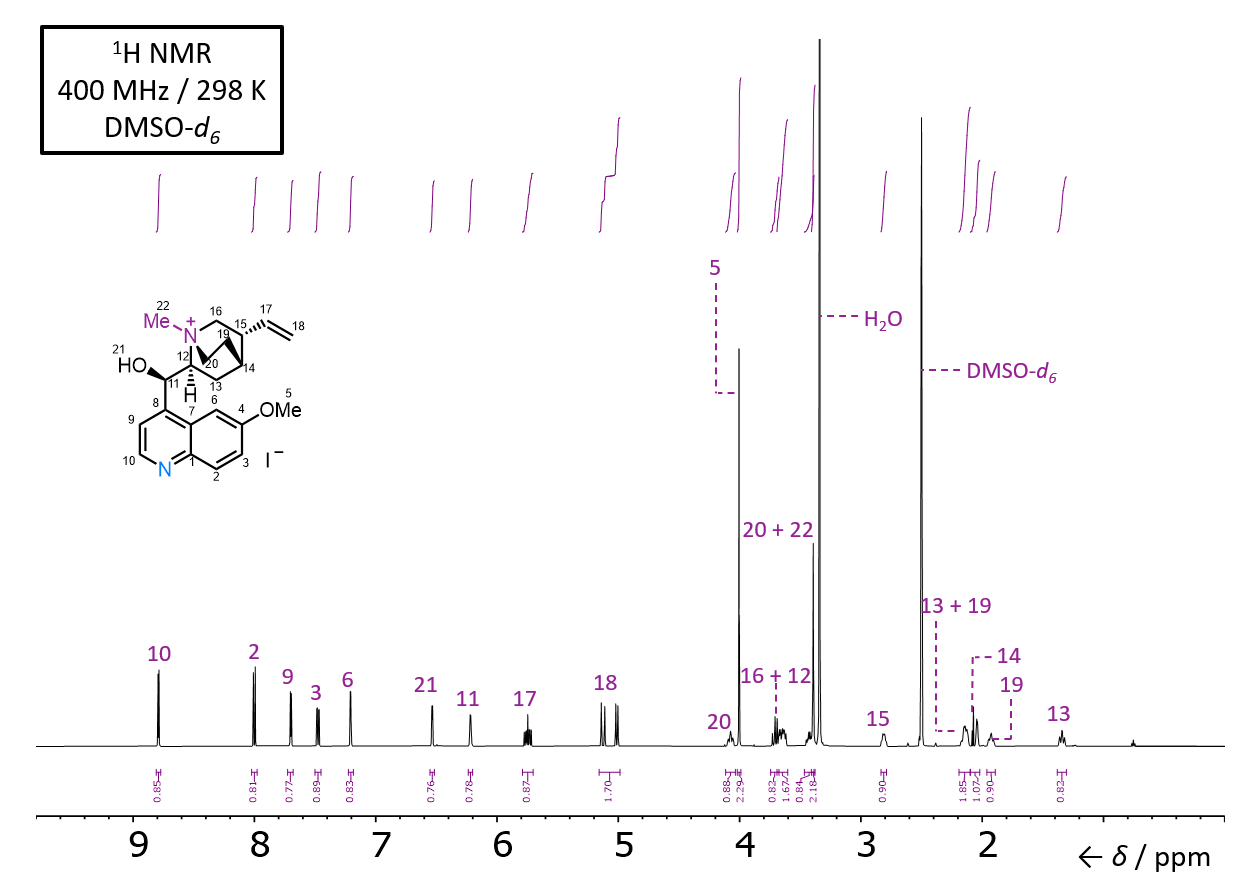


**Fig. S1.** ^1^H NMR Spectrum of **MeQn**·I.

**
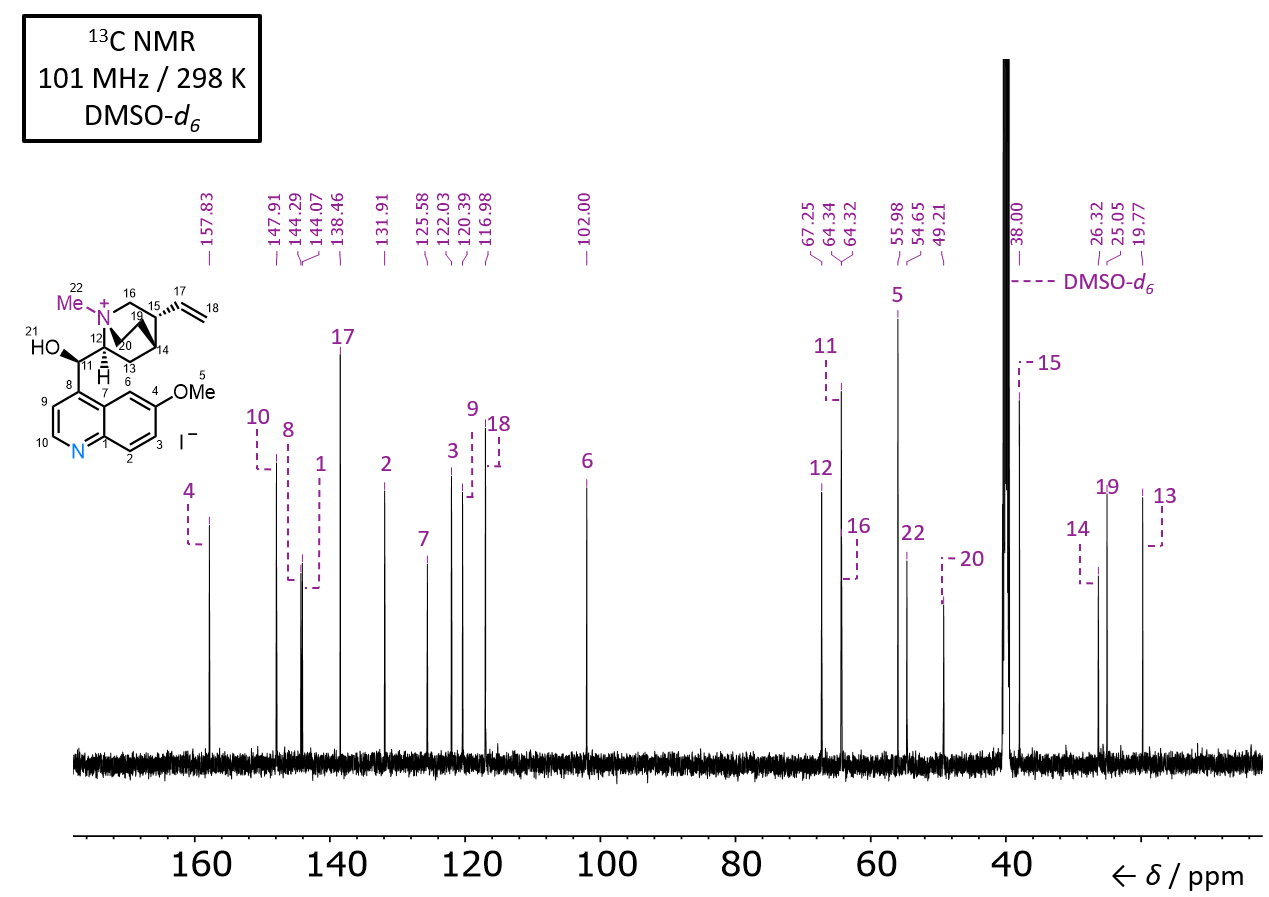
**

**Fig. S2.** ^13^C{^1^H} NMR Spectrum of **MeQn**·I.


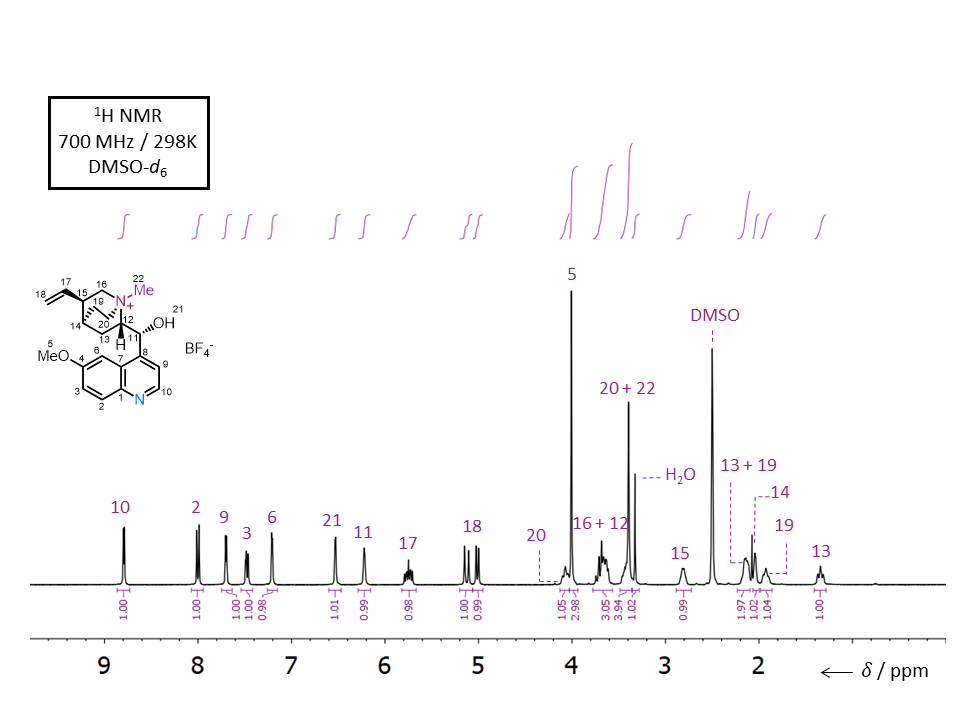

_
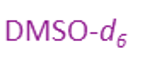
_

**Fig. S3.** ^1^H NMR Spectrum of **MeQn**·BF_4_.

**
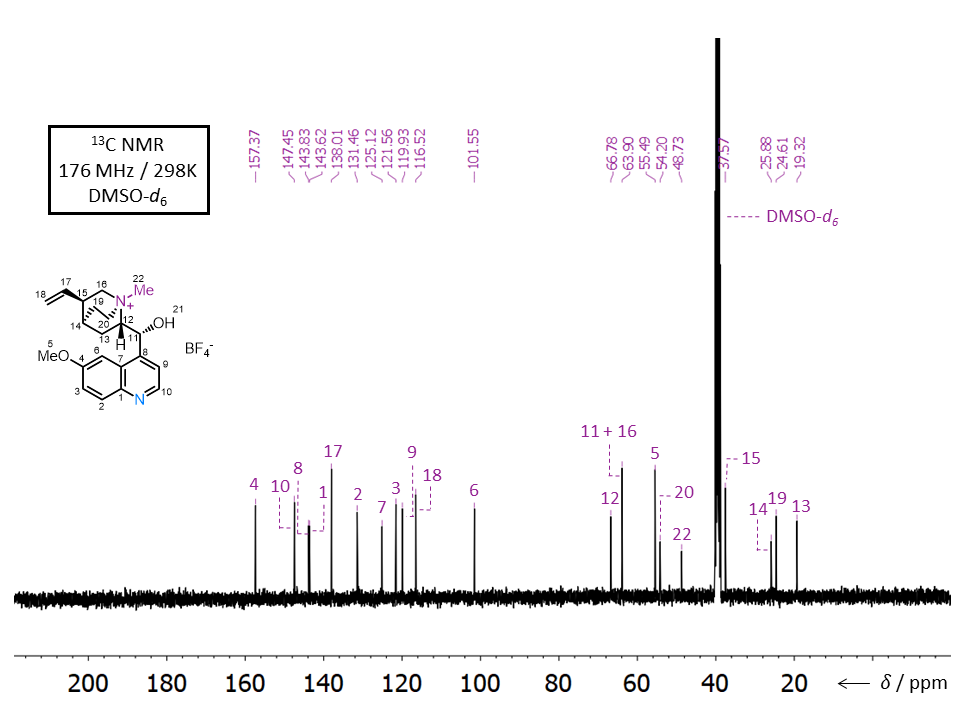
**

**Fig. S4.** ^13^C{^1^H} NMR Spectrum of **MeQn**·BF_4_.


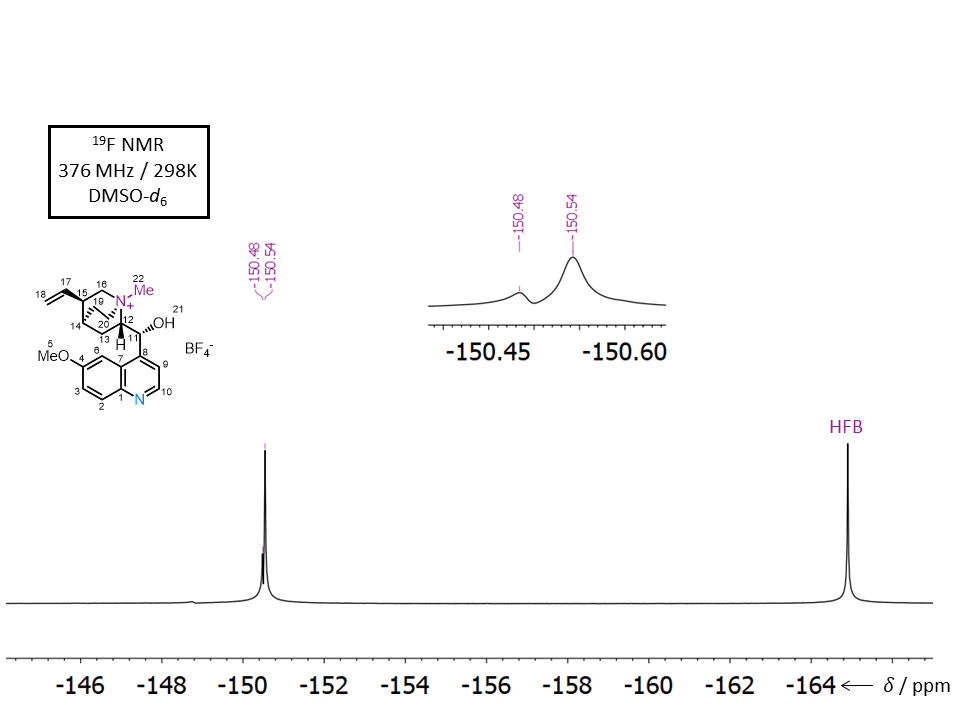

**
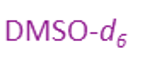
**

**Fig. S5.** ^19^F NMR Spectrum of **MeQn**·BF_4_.


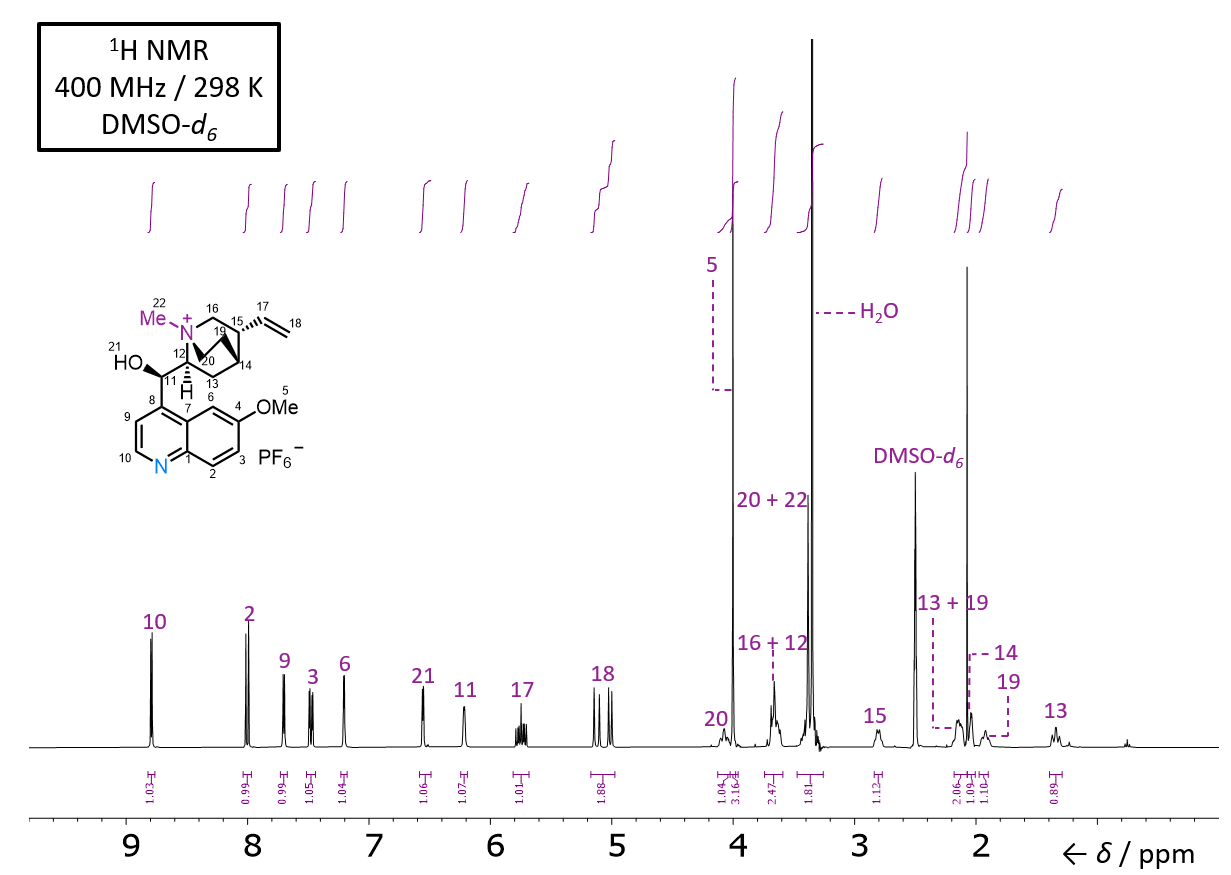


**Fig. S6.** ^1^H NMR Spectrum of **MeQn**·PF_6_.

_
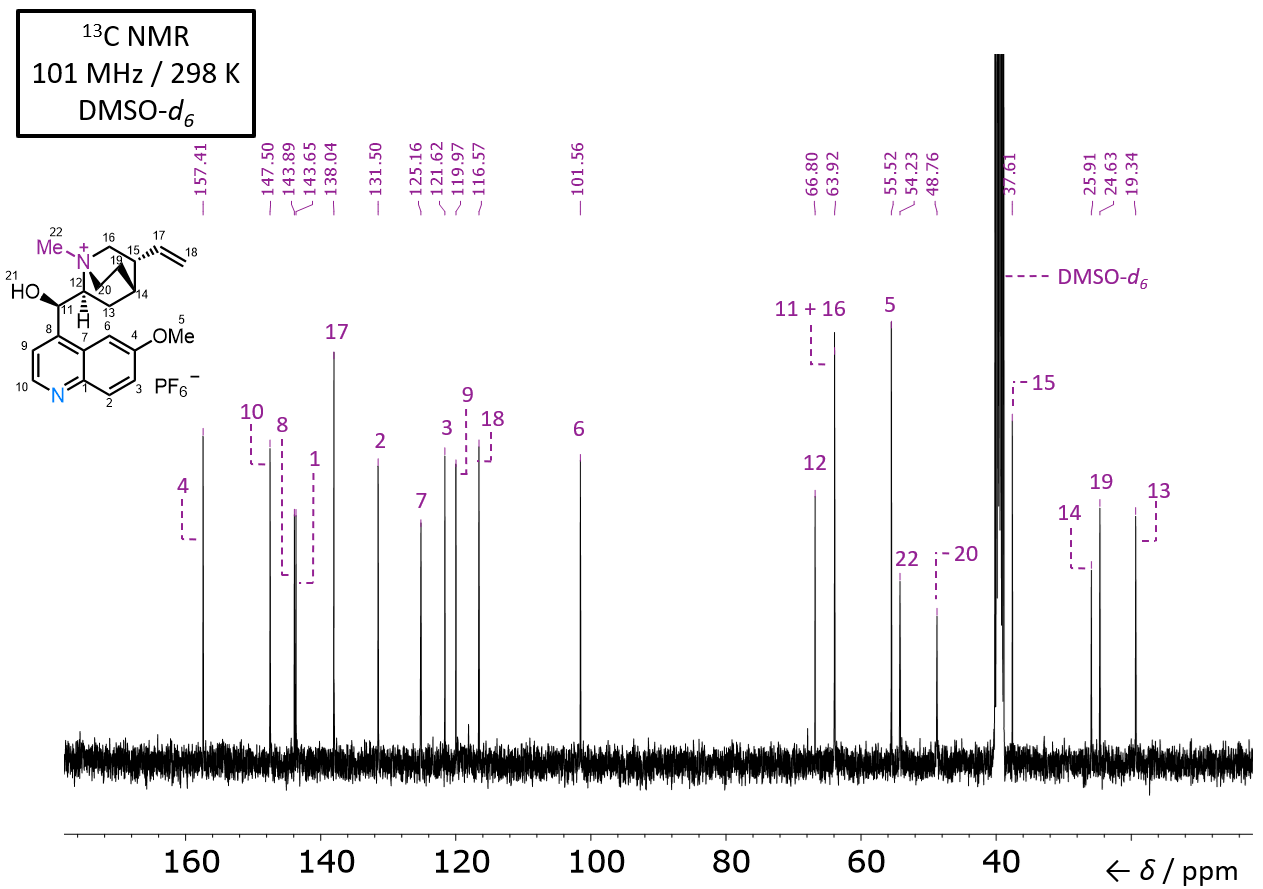
_

**Fig. S7.** ^13^C{^1^H} NMR Spectrum of **MeQn**·PF_6_.


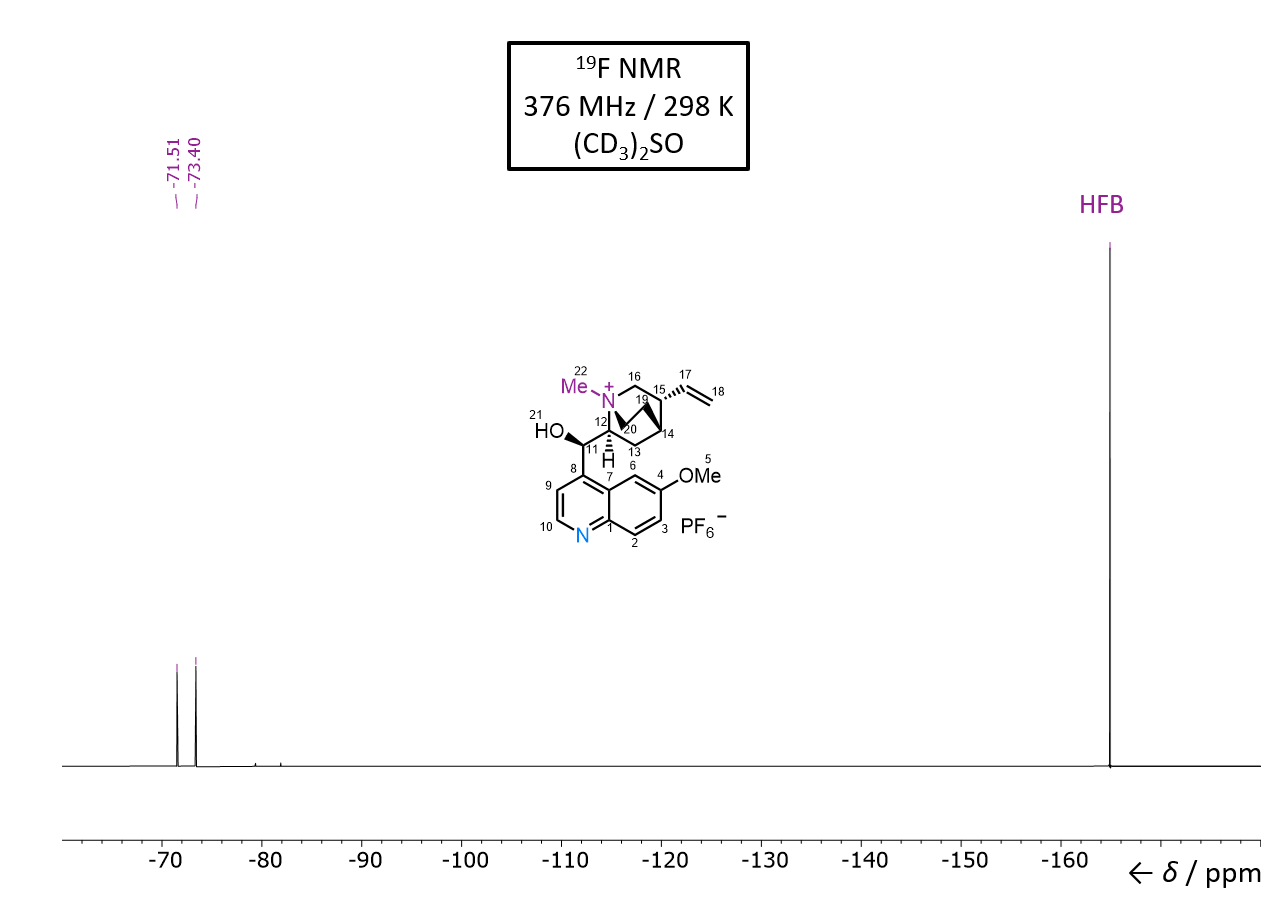

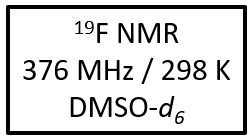


**Fig. S8.** ^19^F NMR Spectrum of **MeQn**·PF_6_.


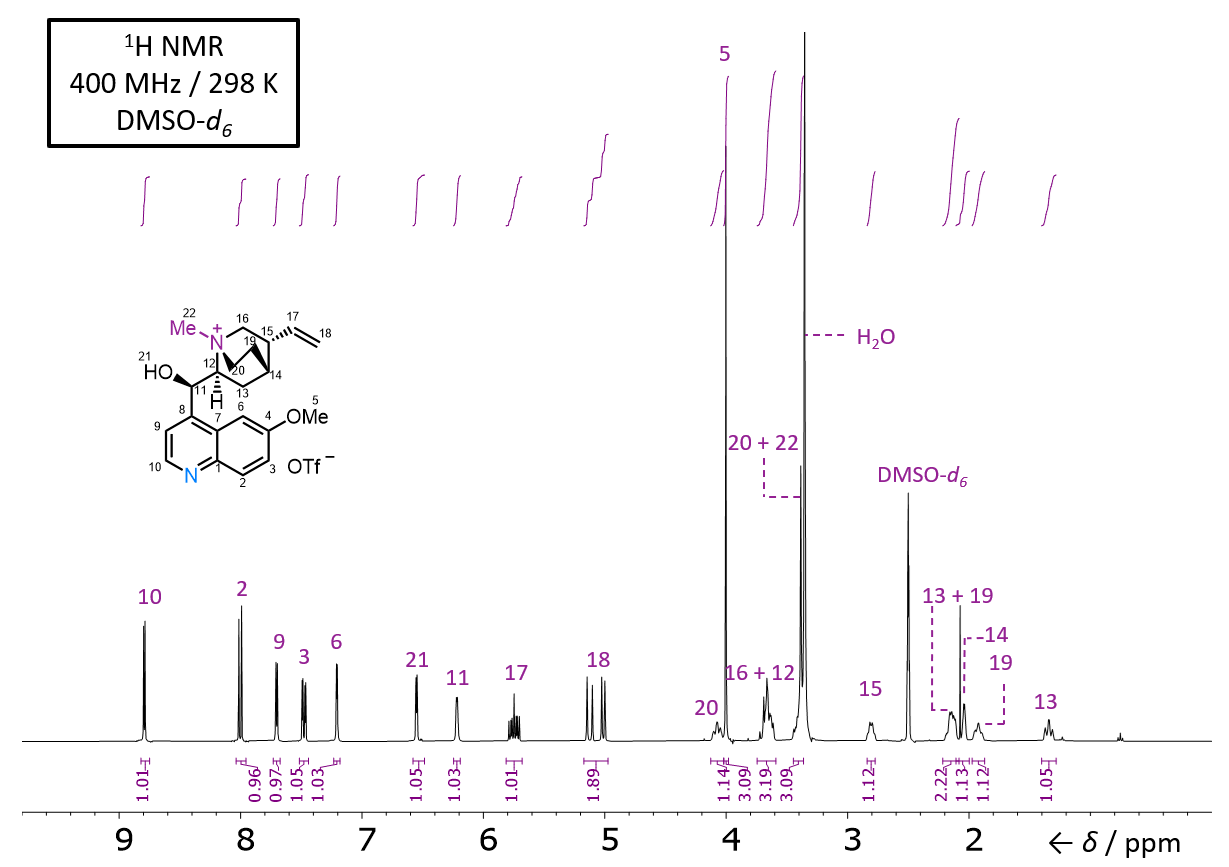


**Fig. S9.** ^1^H NMR Spectrum of **MeQn**·OTf.


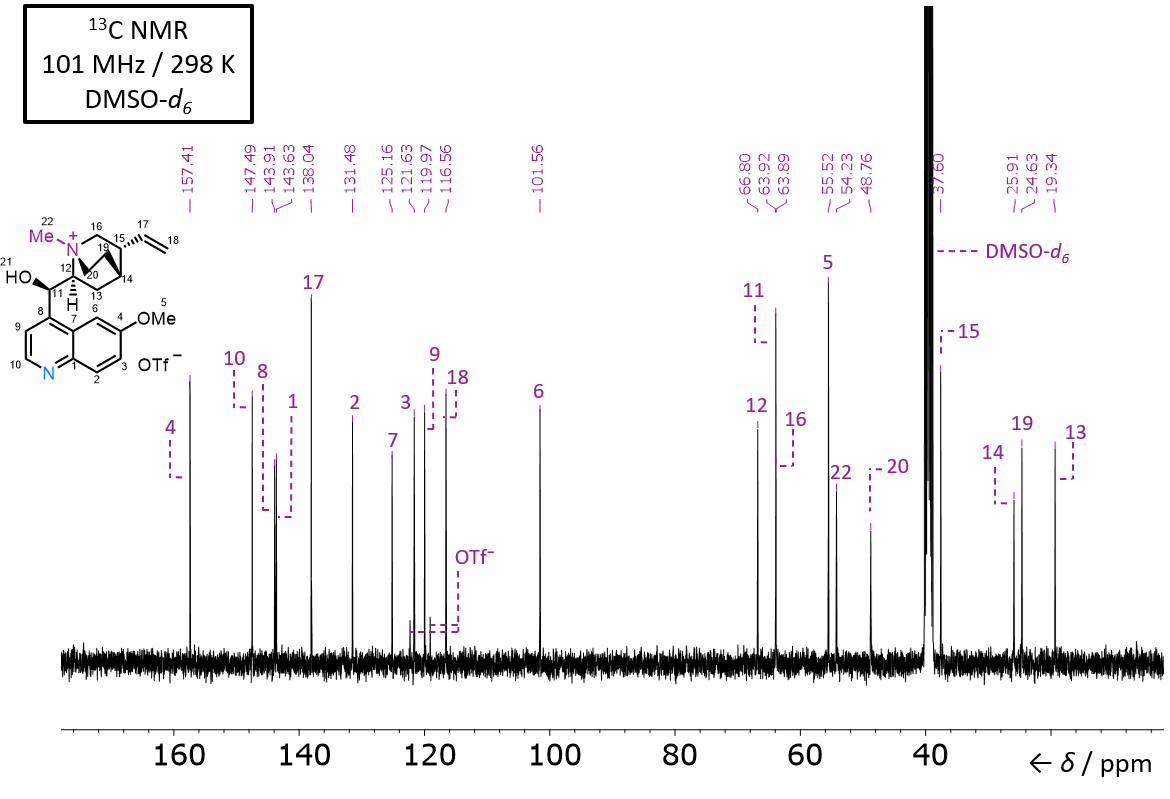


**Fig. S10.** ^13^C{^1^H} NMR Spectrum of **MeQn**·OTf.


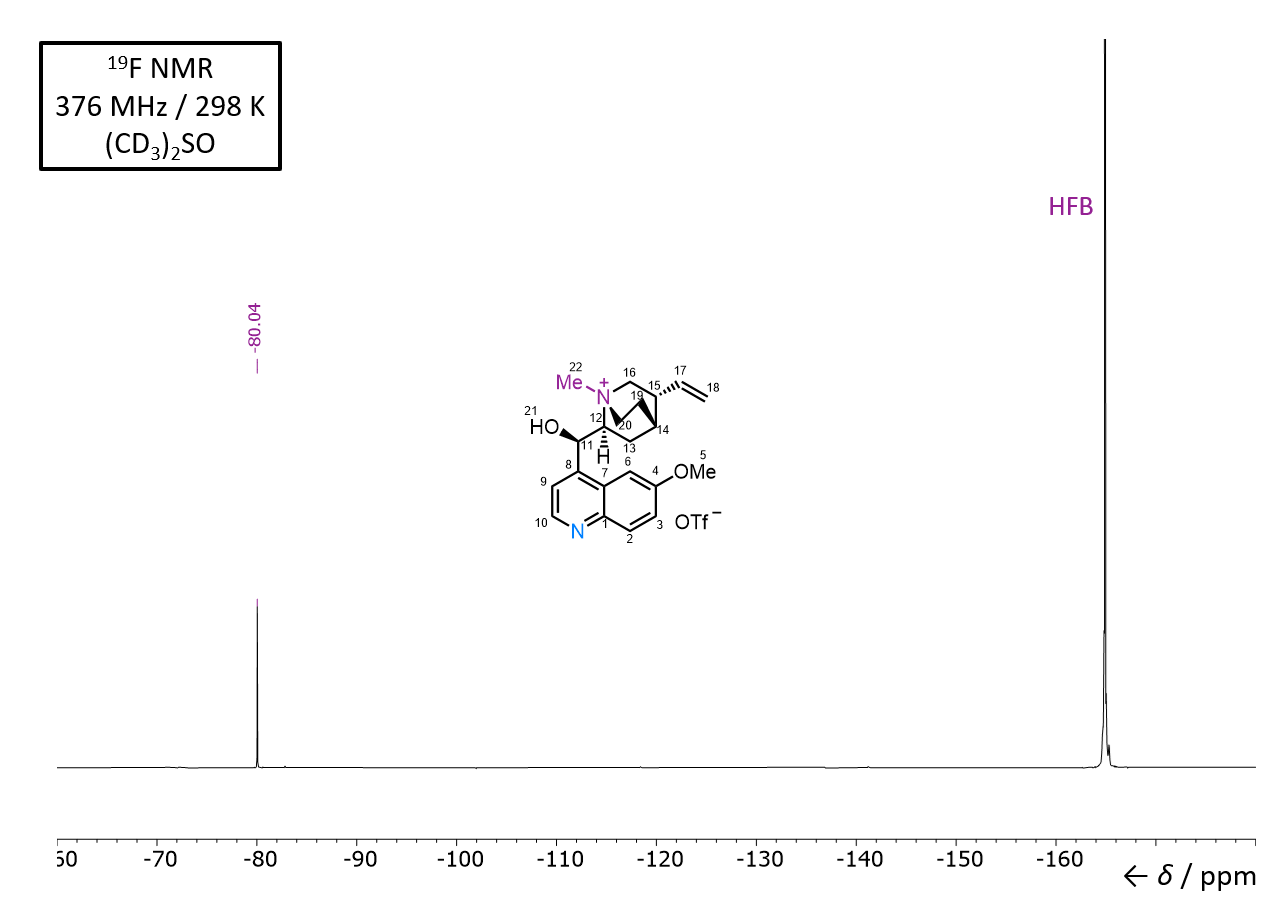

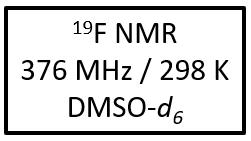


**Fig. S11.** ^19^F NMR Spectrum of **MeQn**·OTf.


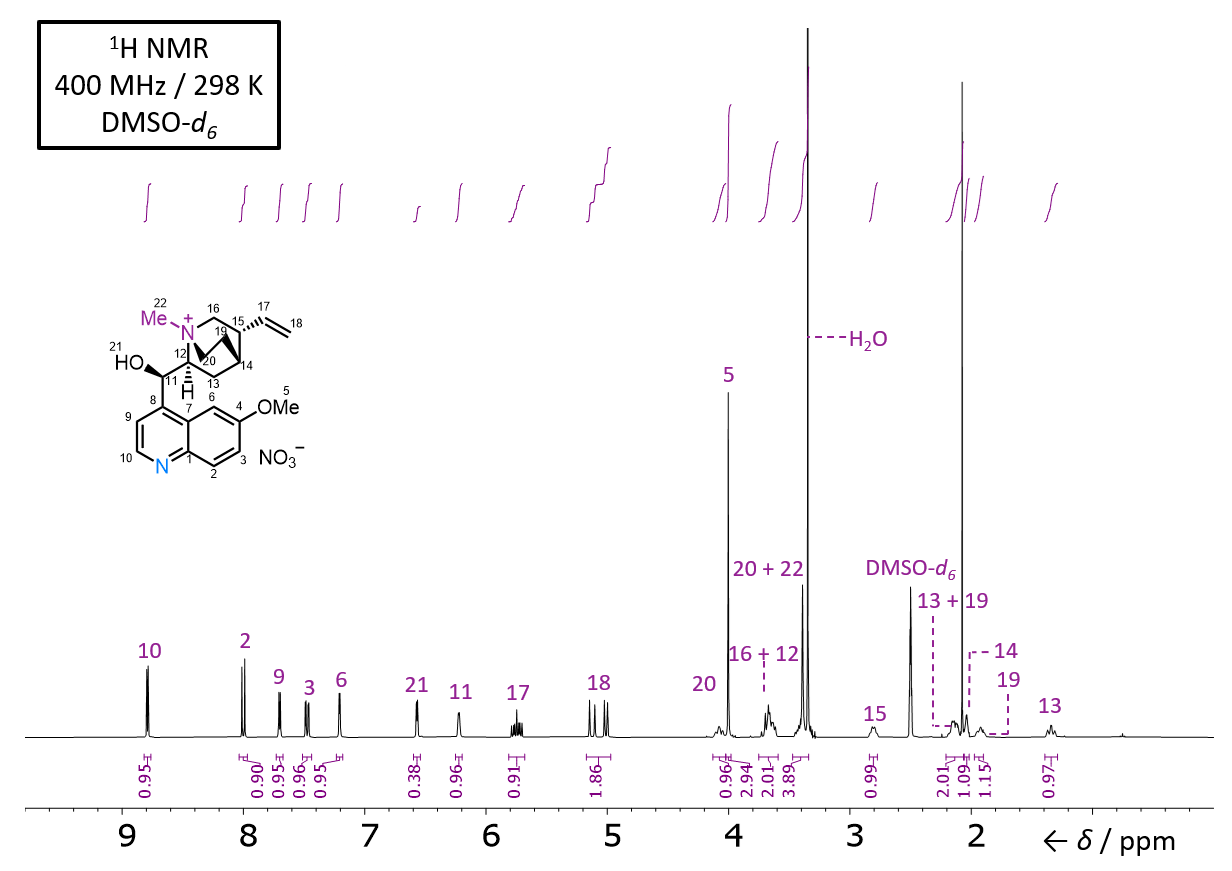


**Fig. S12.** ^1^H NMR Spectrum of **MeQn**·NO_3_.


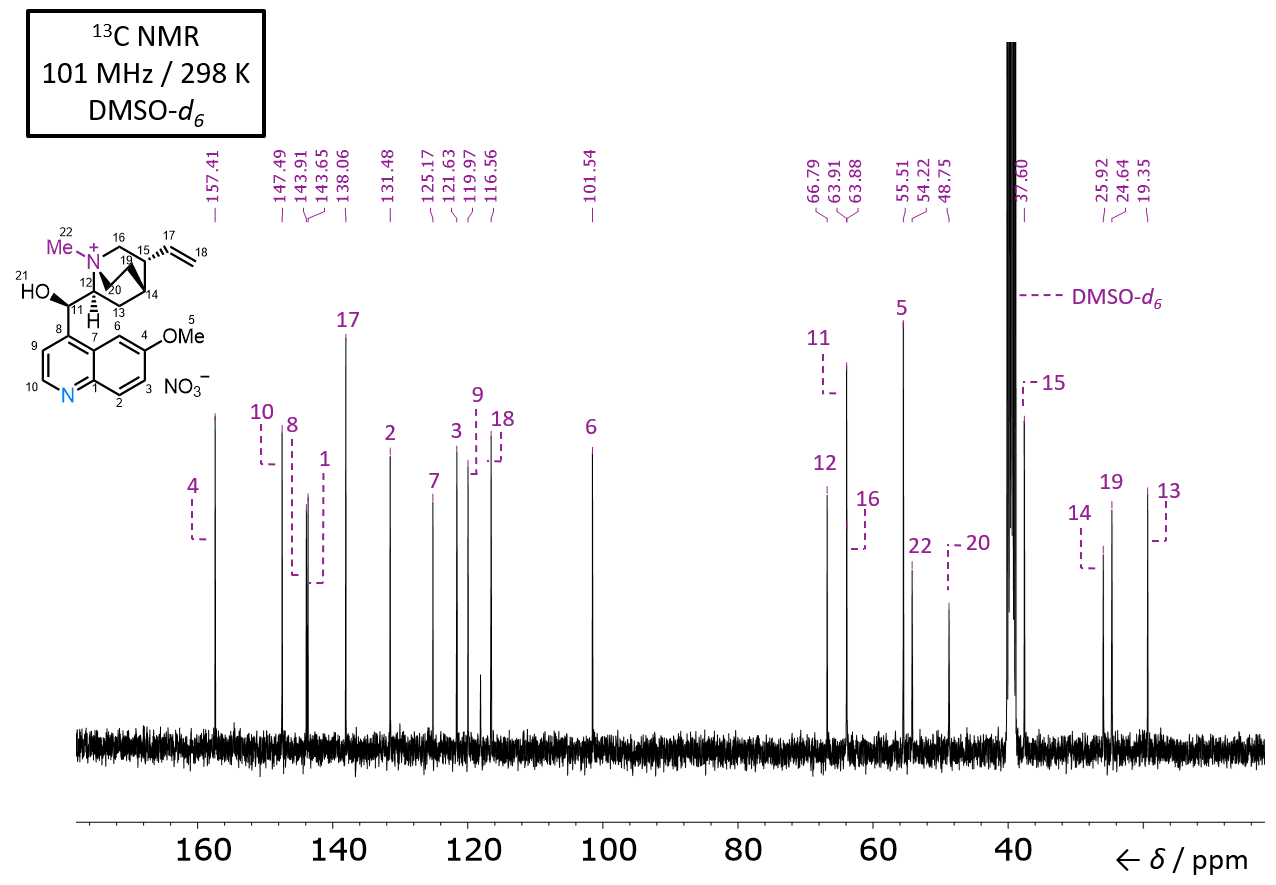


**Fig. S13.** ^13^C{^1^H} NMR Spectrum of **MeQn**·NO_3_.


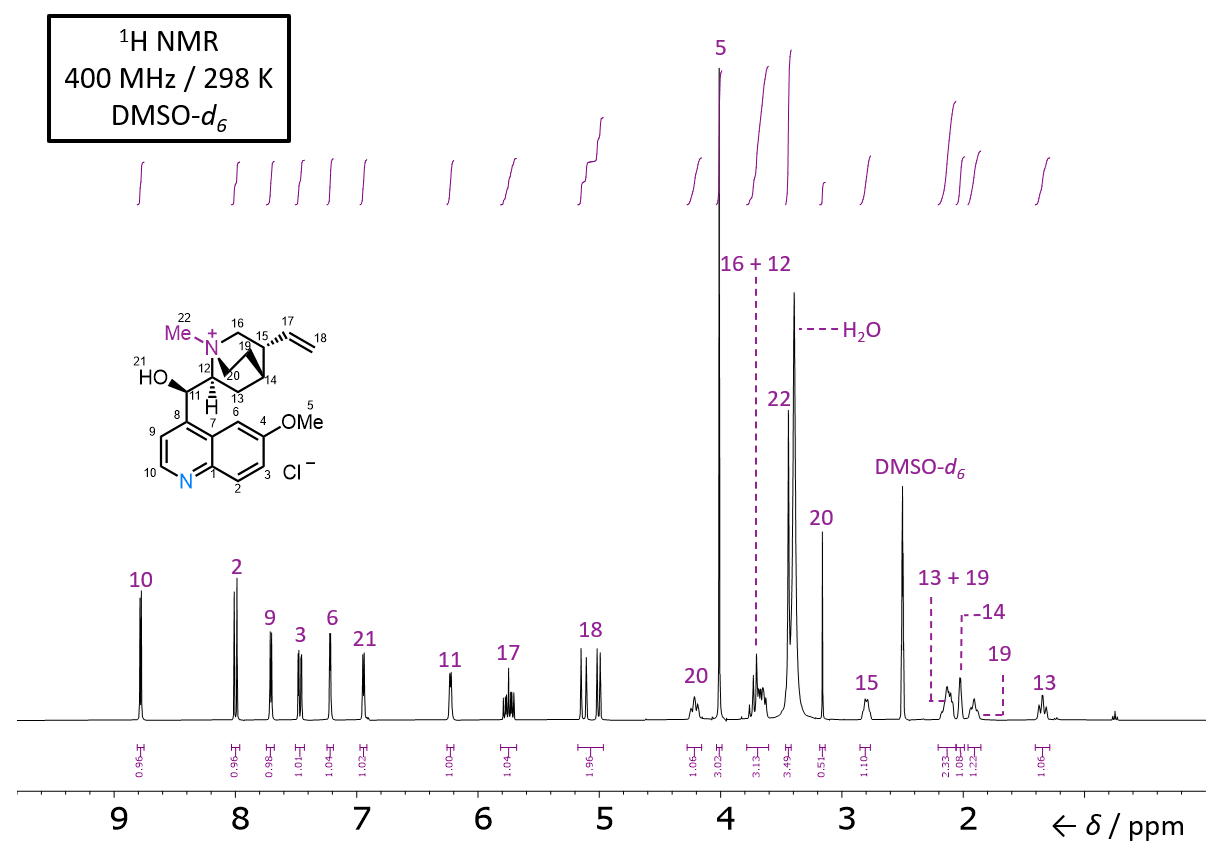


**Fig. S14.** ^1^H NMR Spectrum of **MeQn**·Cl.


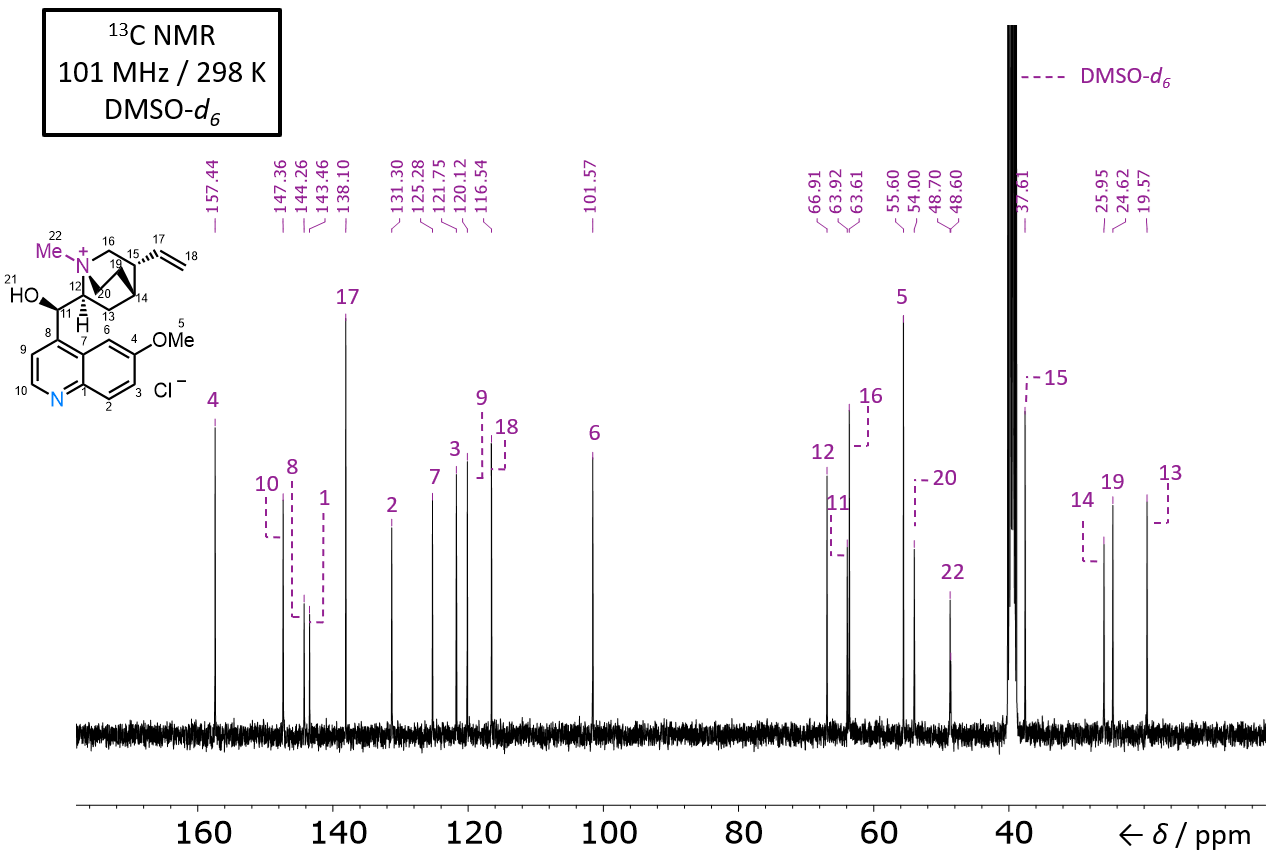


**Fig. S15.** ^13^C{^1^H} NMR Spectrum of **MeQn**·Cl.


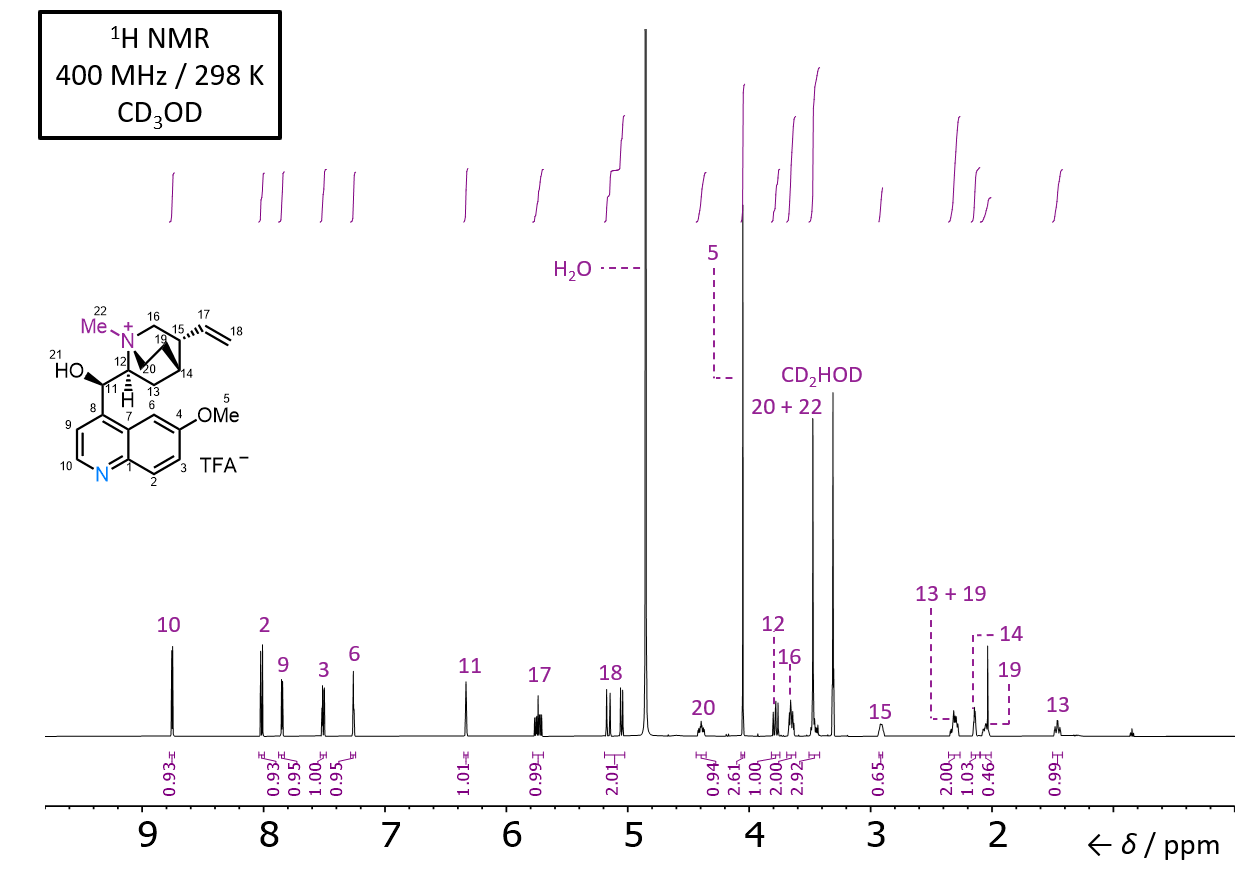


**Fig. S16.** ^1^H NMR Spectrum of **MeQn**·TFA.


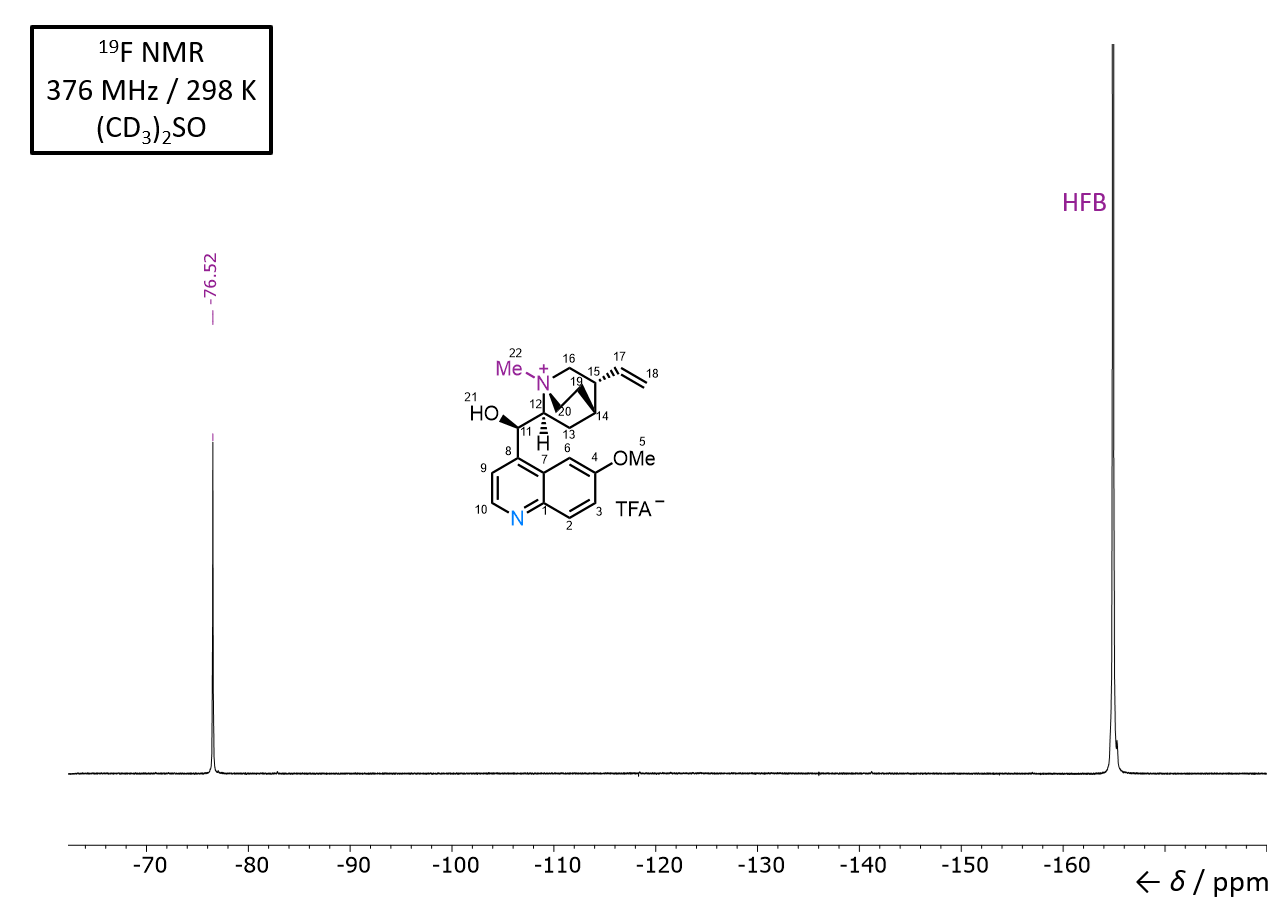

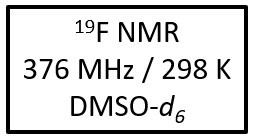


**Fig. S17.** ^19^F NMR Spectrum of **MeQn**·TFA.


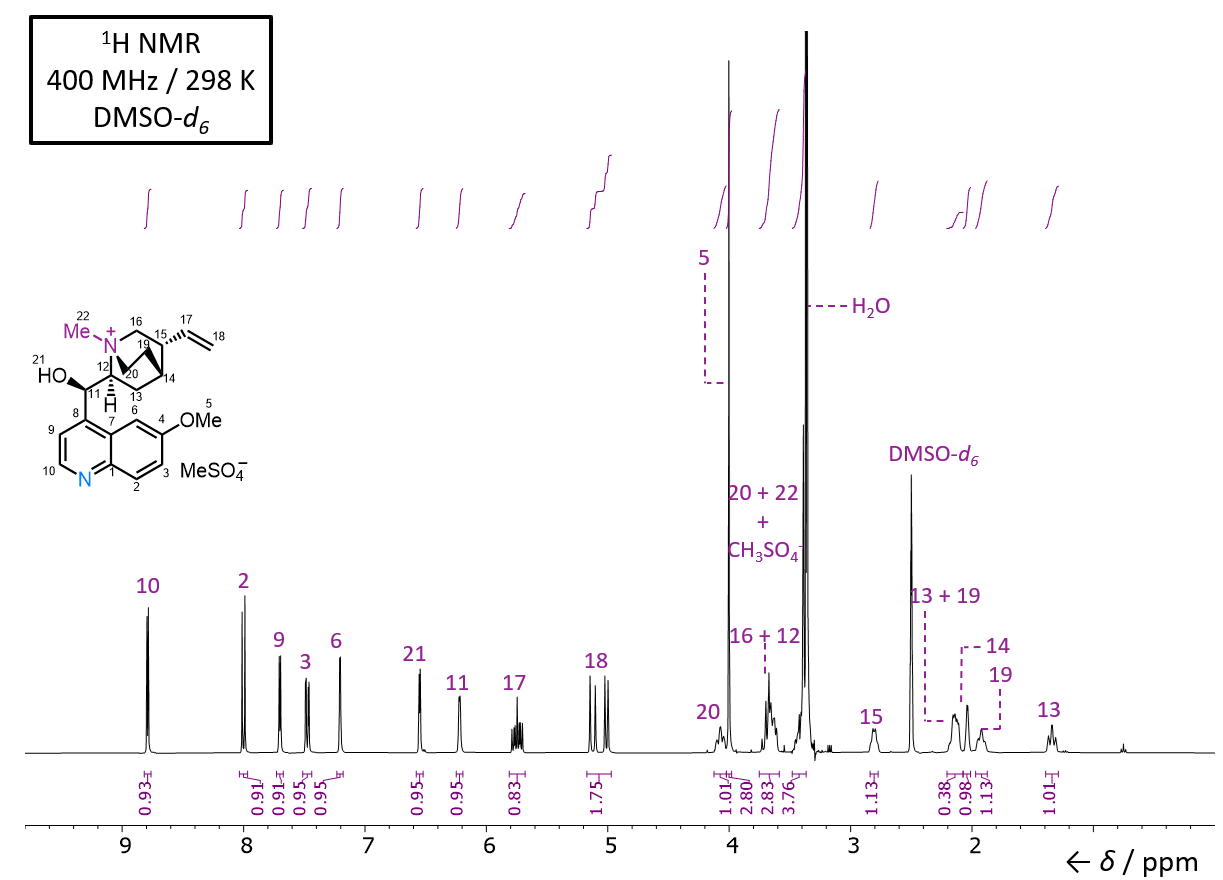


**Fig. S18.** ^1^H NMR Spectrum of **MeQn**·MeSO_4_.


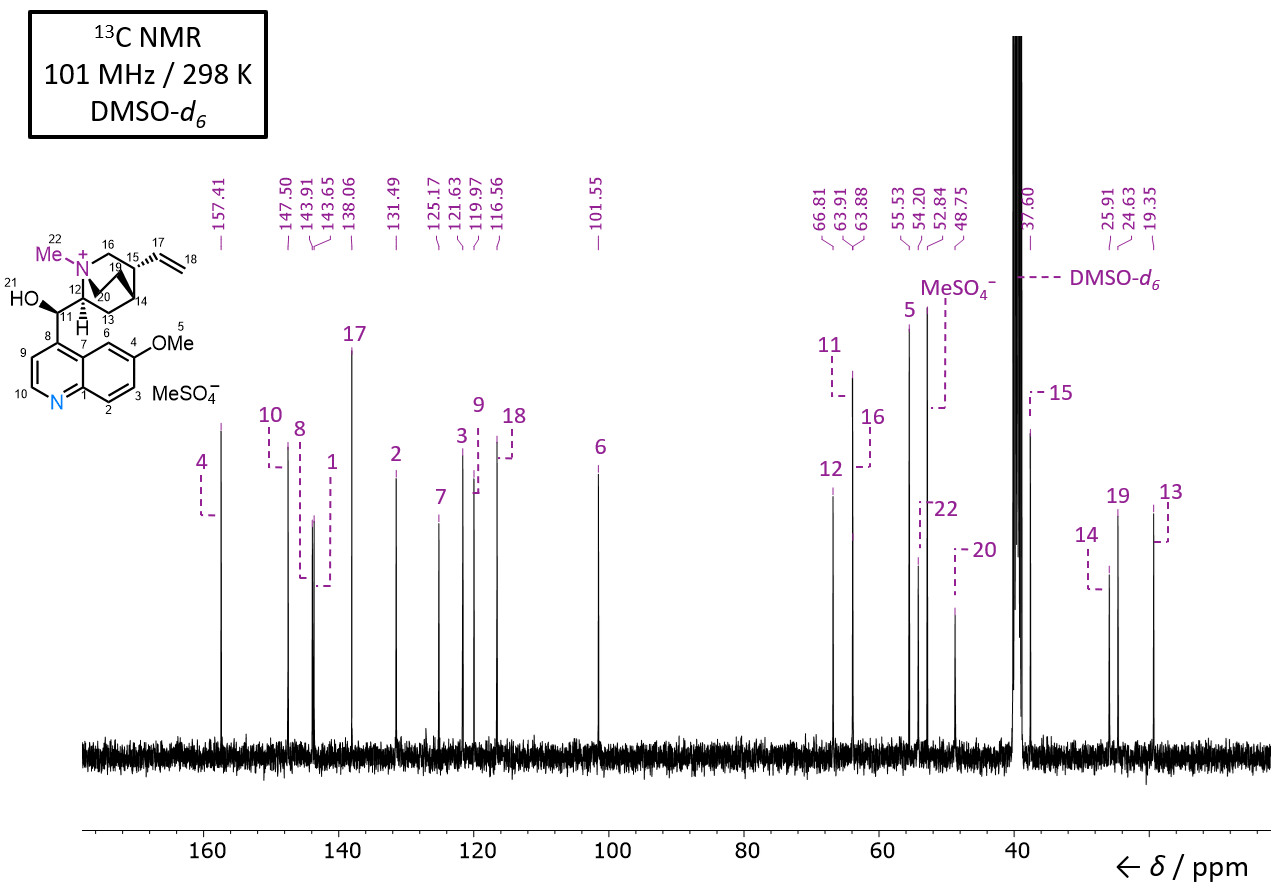


**Fig. S19.** ^13^C{^1^H} NMR Spectrum of **MeQn**·MeSO_4_.


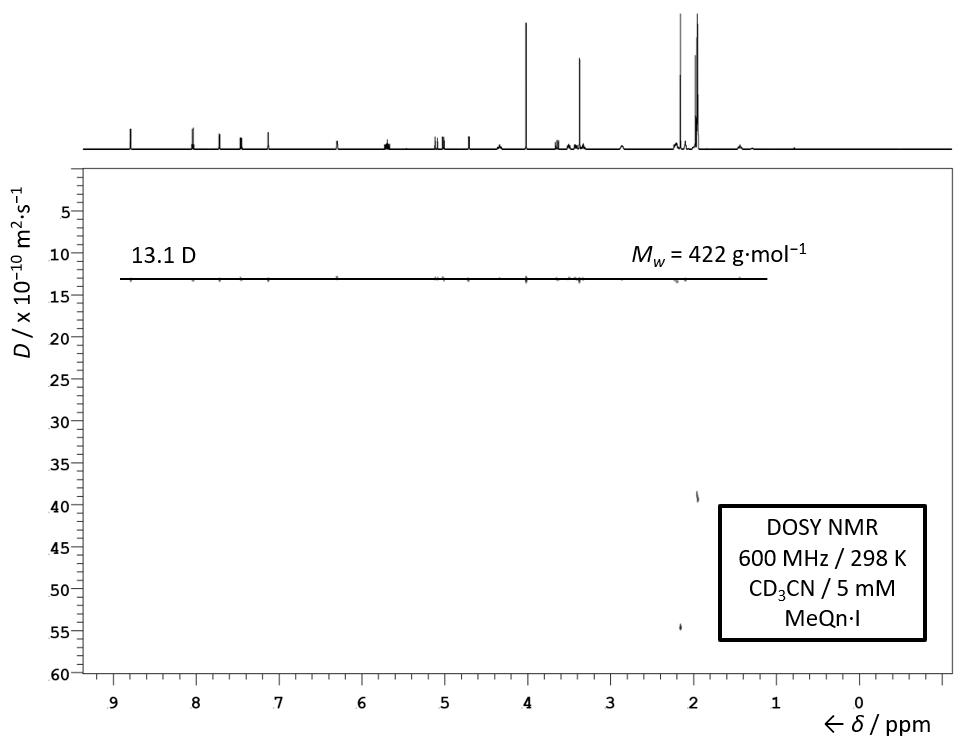
**Convection-compensated ^1^H-DOSY**

**Fig. S20.** Convection-compensated ^1^H-DOSY NMR Spectrum of **MeQn**·I (*c* = 5 mM).


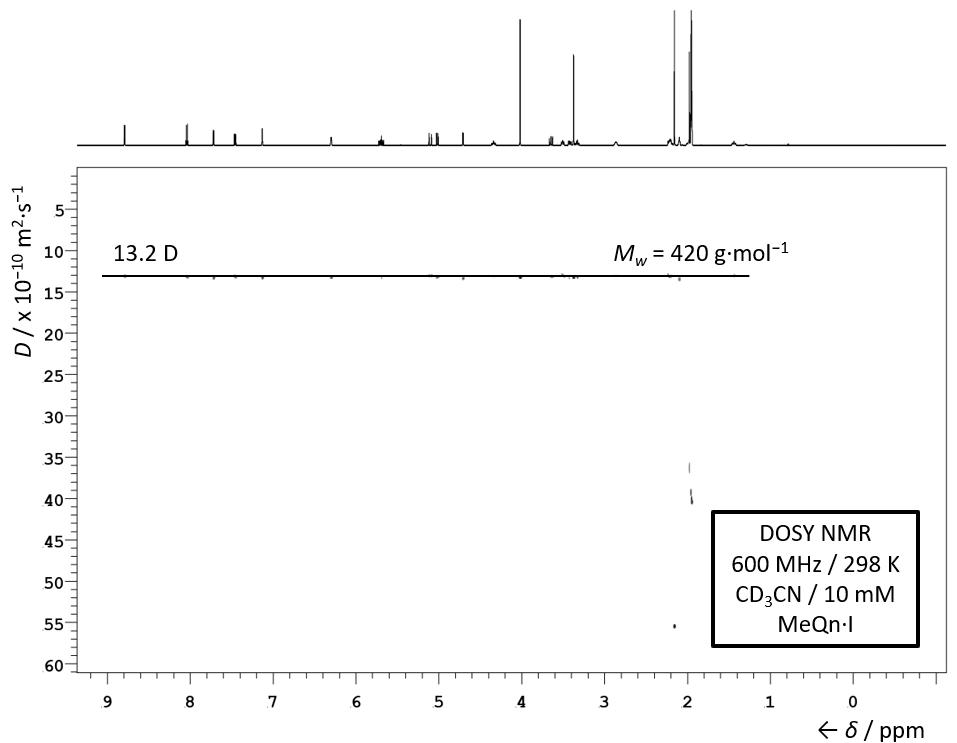


**Fig. S21.** Convection-compensated ^1^H-DOSY NMR Spectrum of **MeQn**·I (*c* = 10 mM).


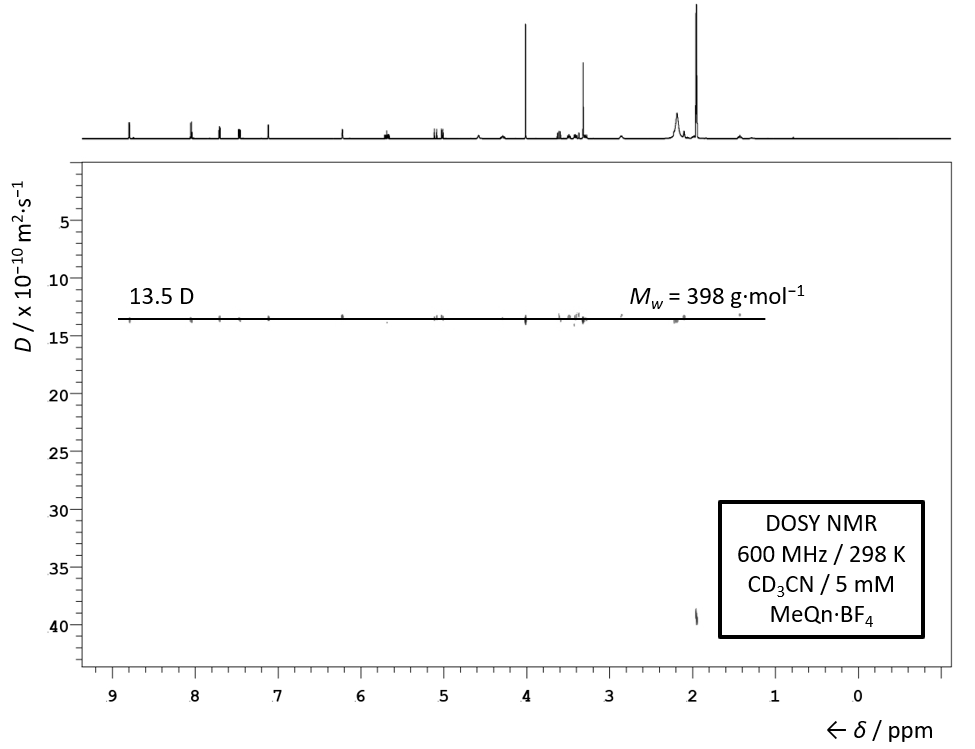


**Fig. S22.** Convection-compensated ^1^H-DOSY NMR Spectrum of **MeQn**·BF_4_ (*c* = 5 mM).


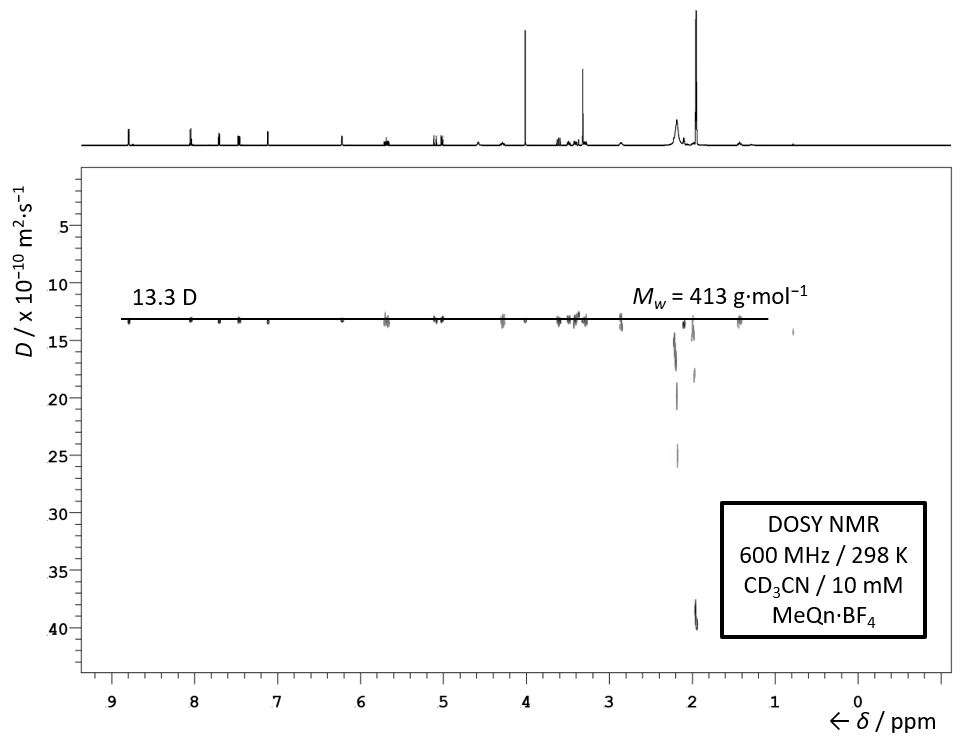


**Fig. S23.** Convection-compensated ^1^H-DOSY NMR Spectrum of **MeQn**·BF_4_ (*c* = 10 mM).


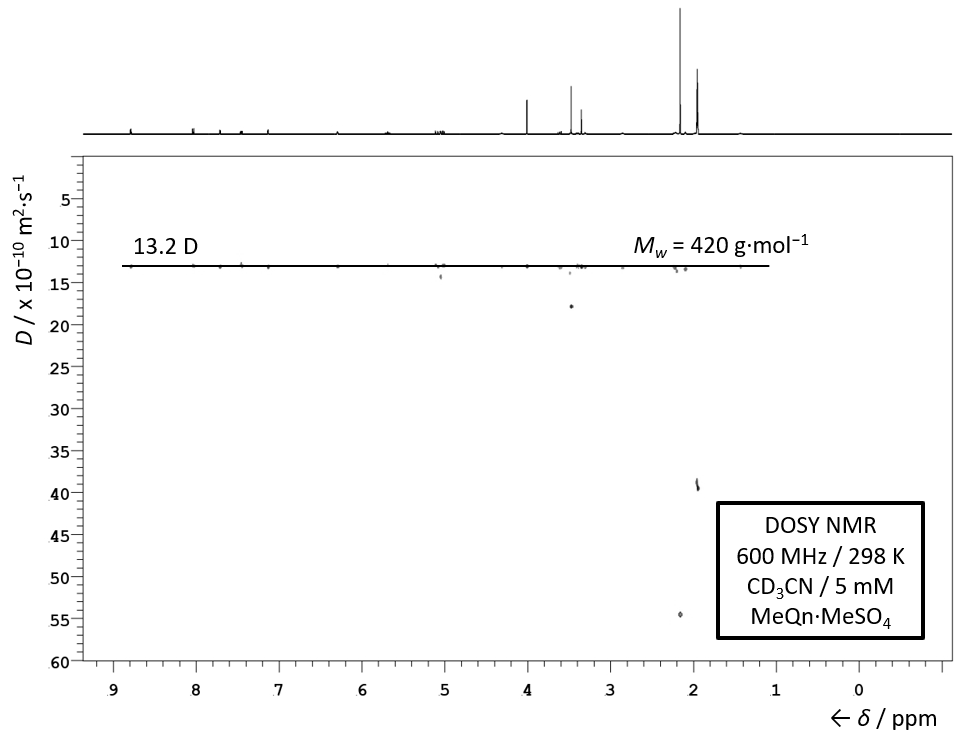


**Fig. S24.** Convection-compensated ^1^H-DOSY NMR Spectrum of **MeQn**·MeSO_4_ (*c* = 5 mM).


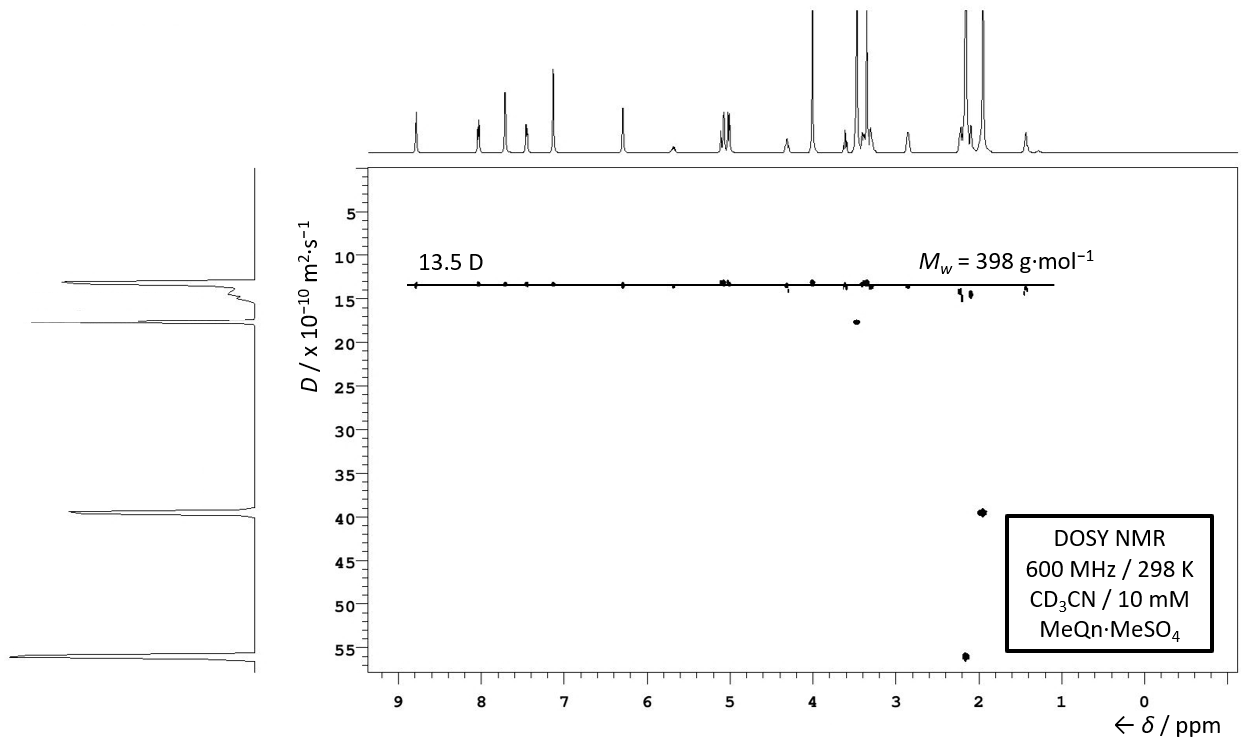


**Fig. S25.** Convection-compensated ^1^H-DOSY NMR Spectrum of **MeQn**·MeSO_4_ (*c* = 10 mM).


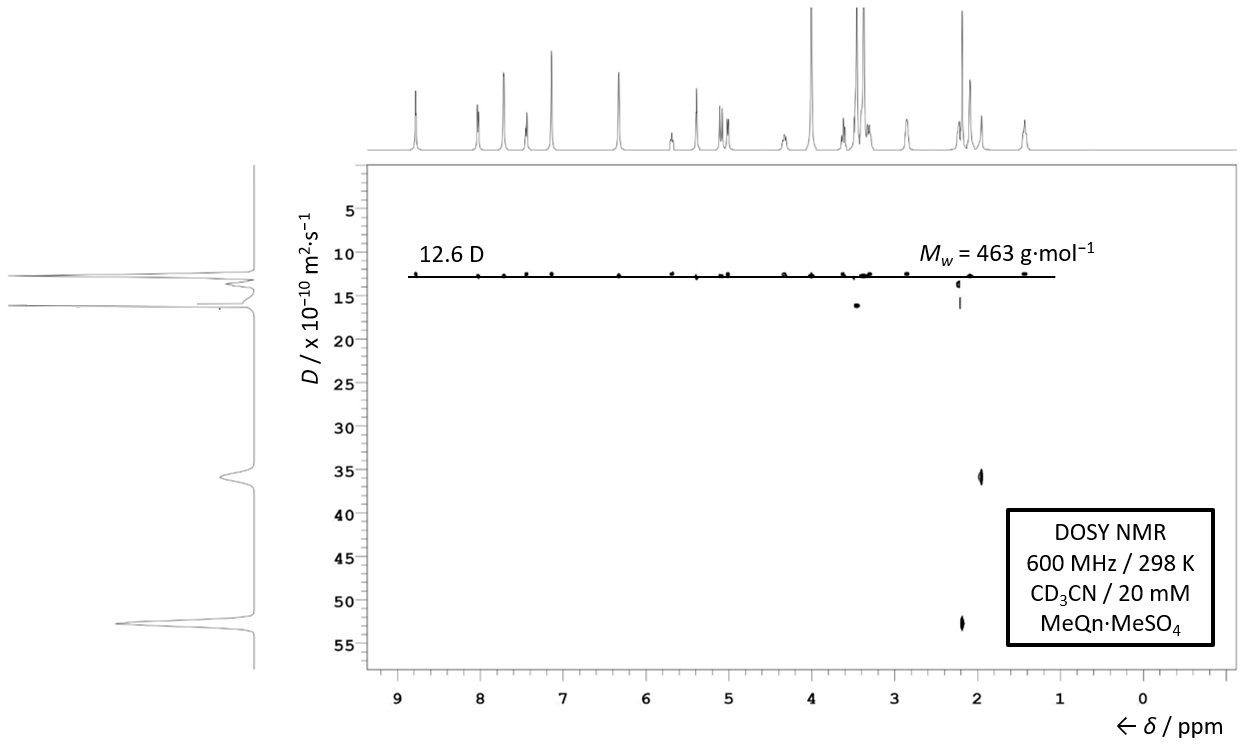


**Fig. S26.** Convection-compensated ^1^H-DOSY NMR Spectrum of **MeQn**·MeSO_4_ (*c* = 20 mM).

**Table S1.** Diffusion coefficients, D, measured by DOSY NMR.

| **Counterion** | **Concentration / mM** | **Diffusion Coefficient / ×10^−10^ m^2^·s^−1^** |
| --- | --- | --- |
| I^−^ | 5 | 13.1 |
| I^−^ | 10 | 13.2 |
| BF_4_^−^ | 5 | 13.5 |
| BF_4_^−^ | 10 | 13.3 |
| MeSO_4_^−^ | 5 | 13.2 |
| MeSO_4_^−^ | 10 | 13.5 |
| MeSO_4_^−^ | 20 | 12.6 |

**NMR Titration**

The binding affinity of trifluoroacetate for **MeQn^+^** was determined by performing a titration experiment. A stock solution of 5.06 mM **MeQn**·PF_6_ and 320 mM potassium trifluoroacetate in CD_3_CN was added in portions to a solution of 5.06 mM **MeQn**·PF_6_ in CD_3_CN and the ^1^H NMR spectra were recorded after each addition (Figure S27). Changes in NMR chemical shifts of the **MeQn^+^** were modelled with Bindfit using a 2:1 binding model with additive cooperativity that was optimized using the Nelder–Mead algorithm (Figure S28).^[91,92]^

**
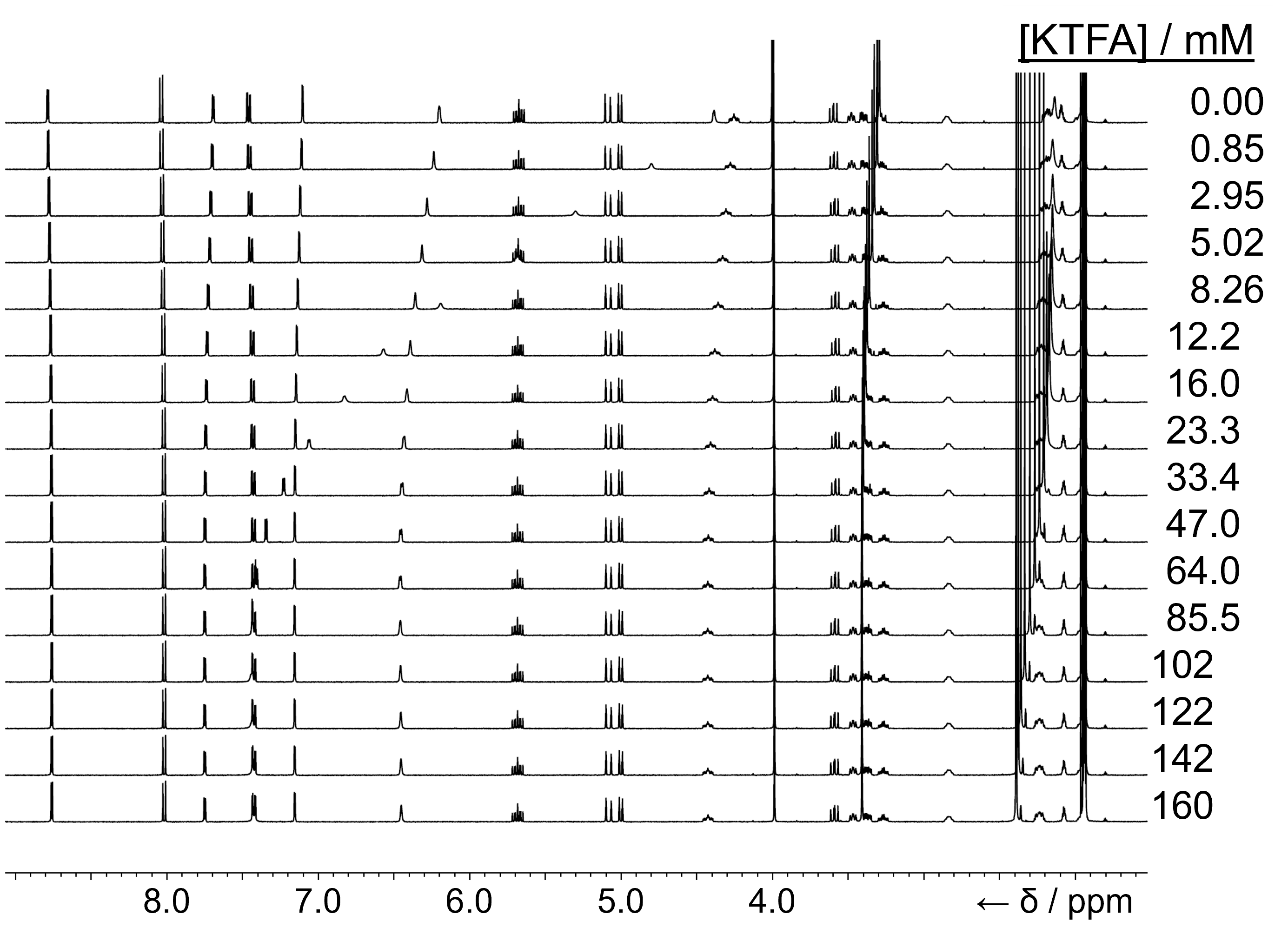
**

**Fig. S27.** ^1^H NMR spectra (500 MHz, 298 K, CD_3_CN) of **MeQn**·PF_6_ (5.06 mM) in the presence of KTFA (0→160 mM).

**
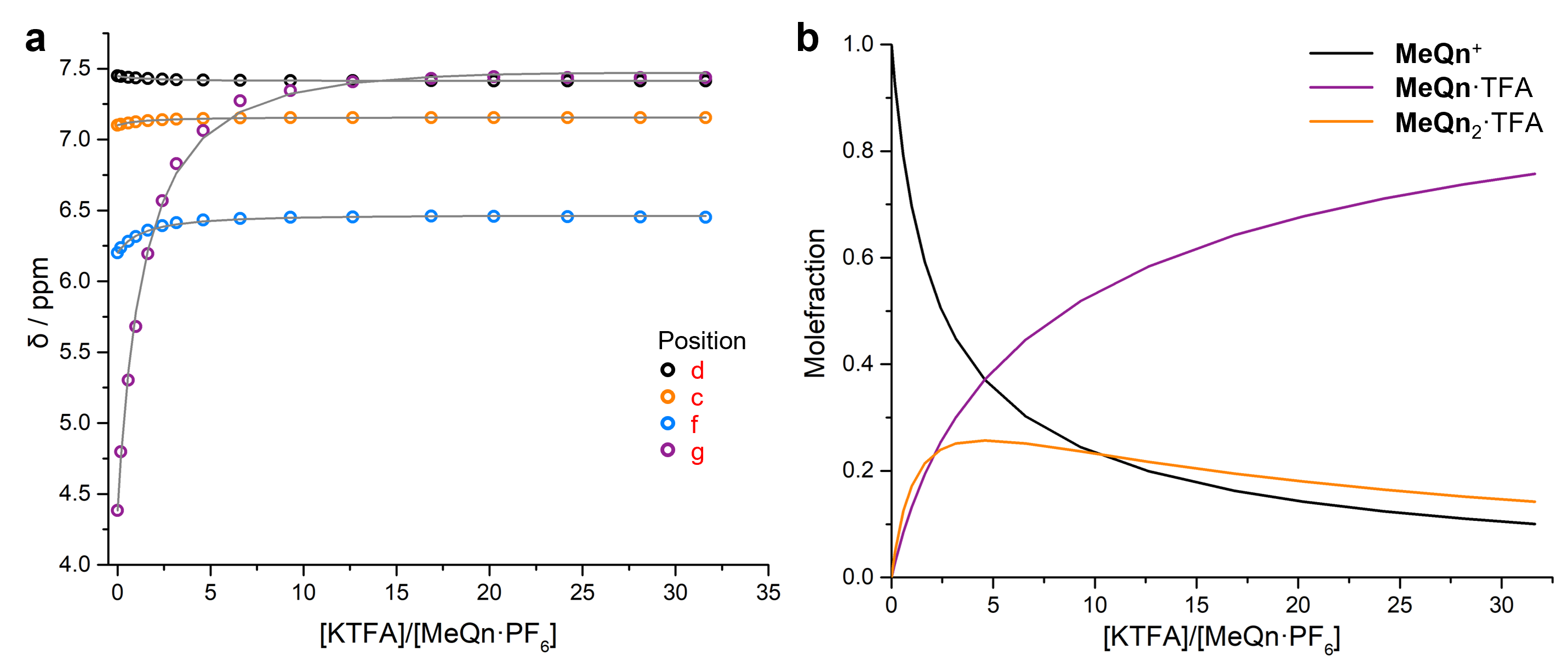
**

**Fig. S28.** NMR titration fitting to a 2:1 binding model. (a) Experimental (circles) and modelled (lines) chemical shift data for four signals. Positional labels correspond to those shown in manuscript Figure 4. (b) Mole fractions of the species modelled at differing ratios of KTFA and **MeQn**·PF_6_.

**4. Photophysical Data**

**
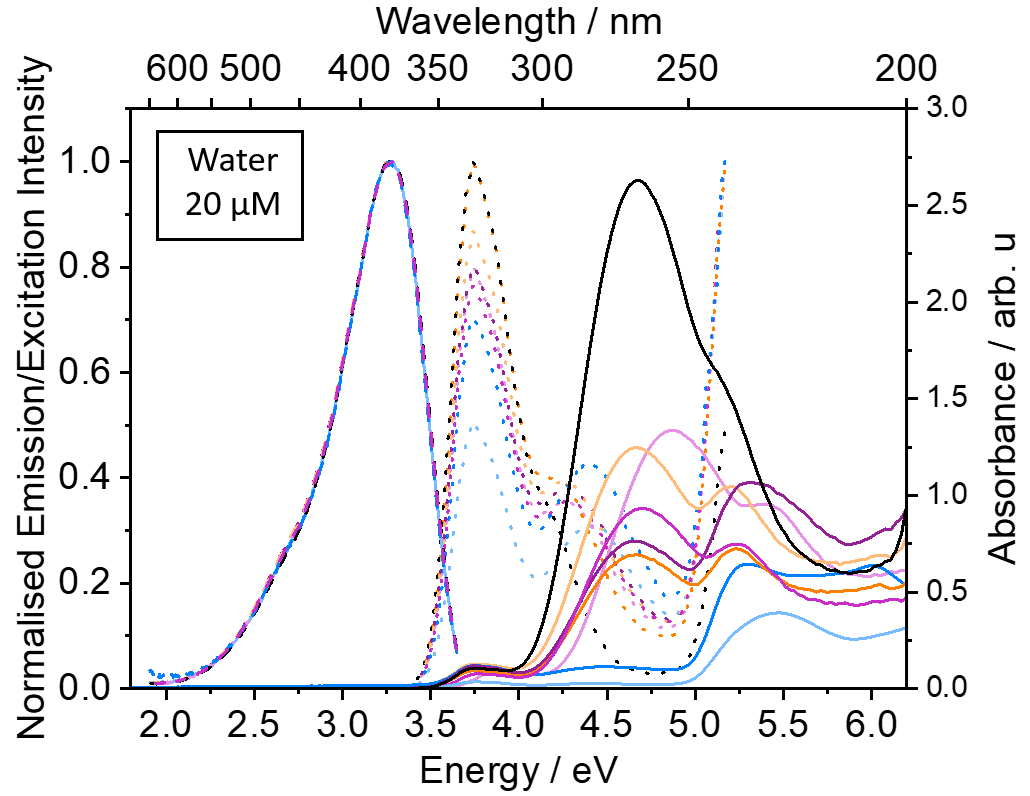

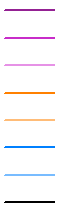
Photophysical Data of Quinine Salts in Dilute Solutions**

I⁻

Cl⁻

NO_3_⁻

BF_4_⁻

PF_6_⁻

MeSO_4_⁻

TFA⁻

OTf⁻

Absorption

Emission

Excitation

**Fig. S29.** UV-vis absorption, normalised emission spectra (*λ*_ex_ = 330 nm) and normalised excitation spectra (*λ*_em_ = 370 nm) of **MeQn·**X salts in dilute solutions of deionized water (*c* = 20 µM). Salts are iodide (dark purple), chloride (purple), nitrate (pale purple), tetrafluoroborate (orange), hexafluorophosphate (pale orange), methyl sulfate (blue), trifluoroacetate (pale blue), triflate (black).

**PLQYs in dilute solutions (*c* = 20 μM)**

**Table S2.** Photoluminescence data for **MeQn·**X salts (*c* = 20 µM) in MeCN.

| X^−^ | PLQY / % | Peak maximum / nm | | Lifetime / ns |
| --- | --- | --- | --- | --- |
|  |  | Absorption | Emission  (*λ*_ex_ = 300 nm) | Monomer |
| I^−^ | 8 | 330 | 375 | 1.94 |
| Cl^−^ | 5 | 330 | 375 | 2.11 |
| NO_3_^−^ | 12 | 330 | 375 | 1.90 |
| BF_4_^−^ | 8 | 330 | 375 | 1.84 |
| PF_6_^−^ | 7 | 330 | 375 | N/A |
| TFA^−^ | N/A | 330 | 375 | 2.30 |
| MeSO_4_^−^ | 8 | 330 | 375 | 1.94 |
| OTf^−^ | 10 | 330 | 375 | 1.84 |

**Circular dichroism**

Samples for circular dichroism (CD) spectroscopy were prepared fresh using HPLC grade solvent and their optical densities (OD) confirmed to fall between 0.6–1.0 by absorption spectroscopy prior to CD experiments. CD was measured applying the following acquisition parameters: data integration time (response time) = 4 sec, scan speed = 100 nm min^–1^, bandwidth = 1 nm and sensitivity = 20 mdeg/0.5 dOD (± 20 mdeg range). Sample temperature was regulated using a built-in Peltier thermostat and chiller unit (Julabo). Characteristic signals for the methylquinine salts presented in this work were observed in the 200–600 nm range and averaged over at least 5 accumulations following baseline correction with respect to a blank solvent sample measured under the same experimental conditions. CD data are expressed as ellipticity *θ* in the units of mdeg, which can be thusly converted to molar ellipticity *[θ]* in the historical units of deg·cm^2^·dmol^–1^ according to equation (S1) below:

*[θ]* = *θ* × *M* / (*c* × *l* × 10) (Equation S1)

where *θ* is ellipticity (mdeg), *M* is the molecular weight (g·mol^–1^), *c* is concentration (g·L^–1^), and *l* is the path length of the cell (cm).

**
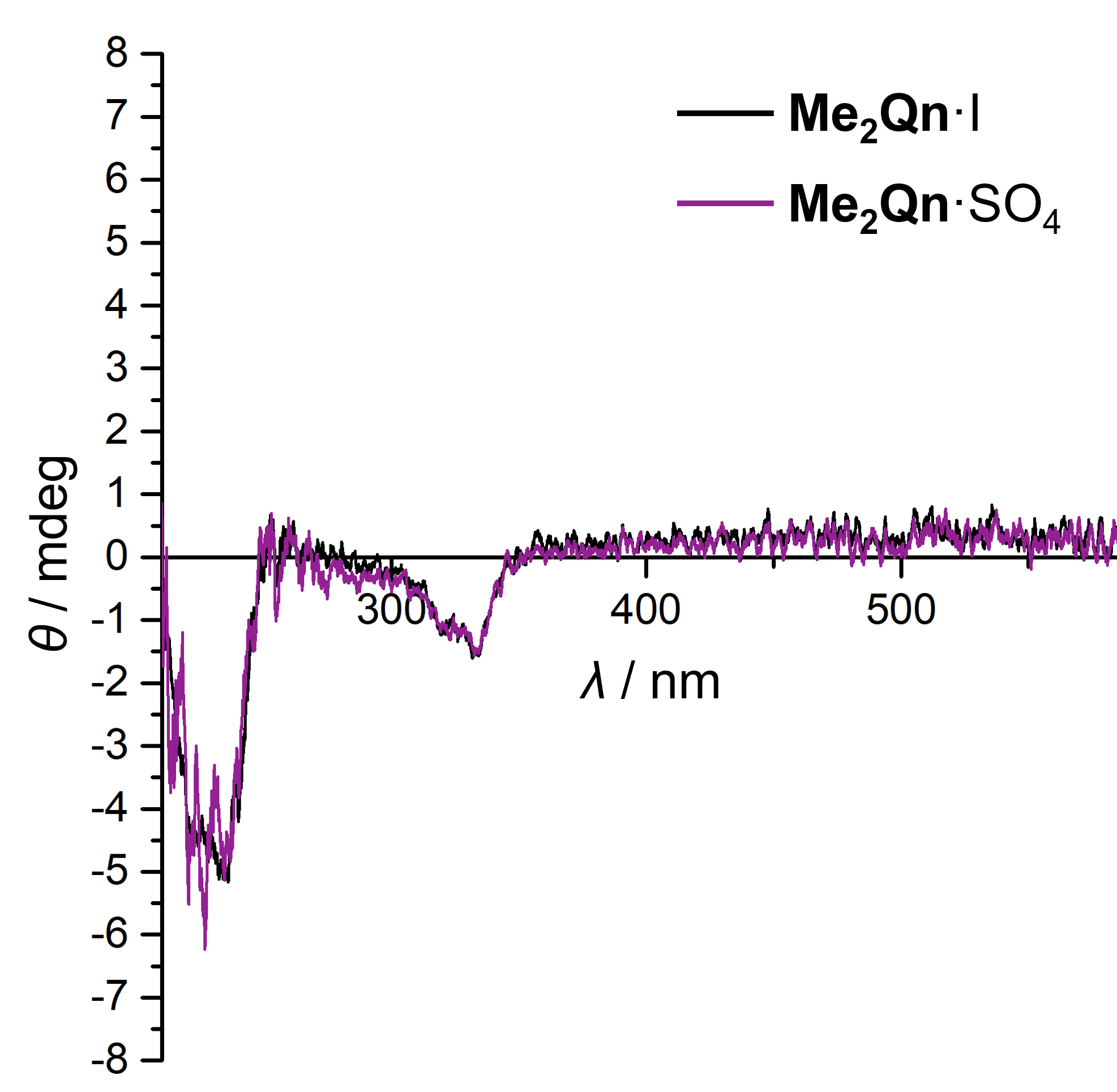
**

**Fig. S30.** Overlaid CD spectra of **MeQn·**I and **MeQn·**MeSO_4_ in MeCN (20 μM).

## **
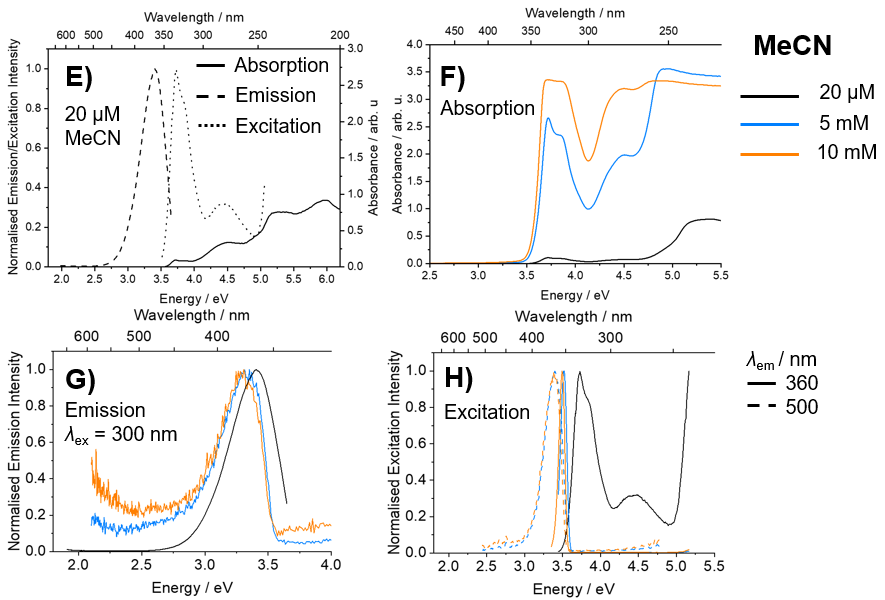
**
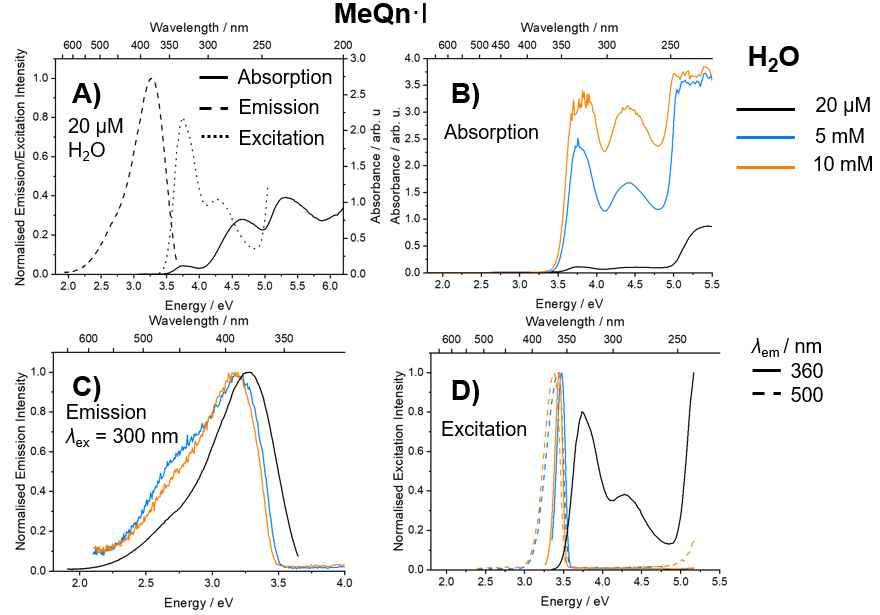
**Concentration studies**

**Fig. S31.** Photophysical data for **MeQn·**I in deionised water (A–D) and MeCN (E–H) at a range of concentrations: *c* = 20 µM (black), 5 mM (blue) and 10 mM (orange). A,E) absorption (solid), emission (dashed) and excitation (dotted) spectra (*c* = 20 µM). B,F) Absorption data for different concentrations. C,G) Emission spectra at a range of concentrations *λ*_ex_ = 300 nm. D,H) Excitation spectra at a range of concentrations *λ*_em_ = 360 nm (solid) and 500 nm (dashed).


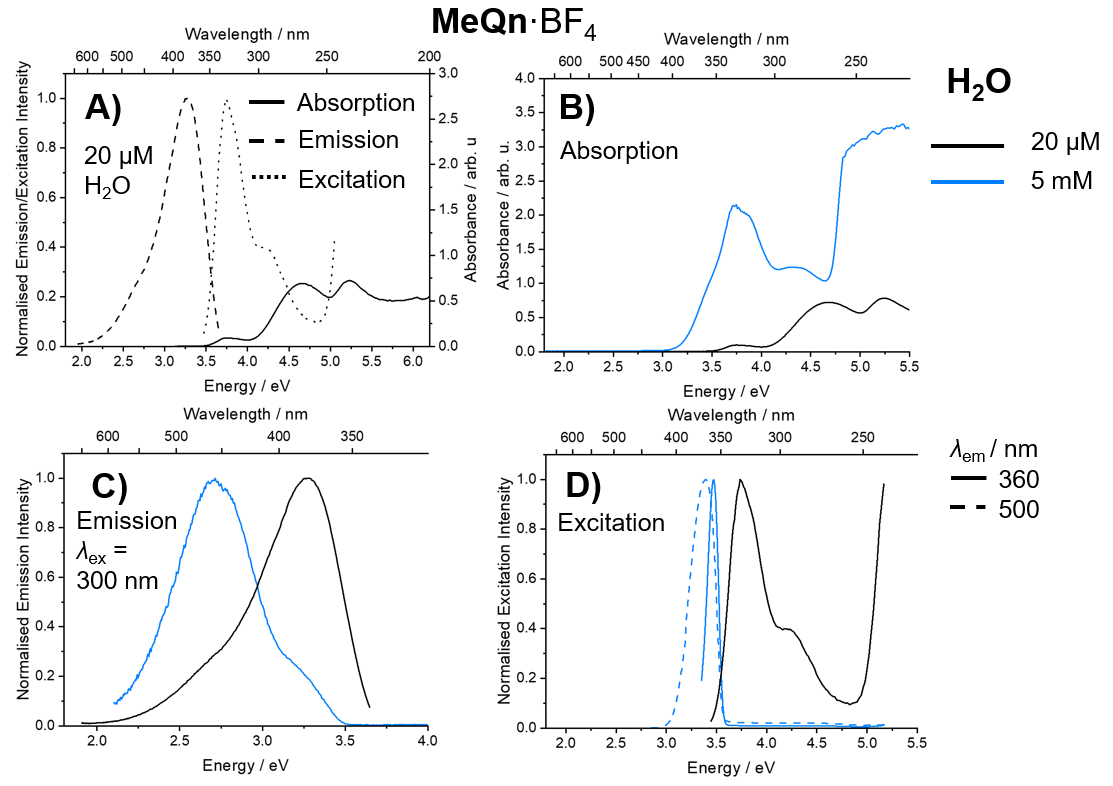
**
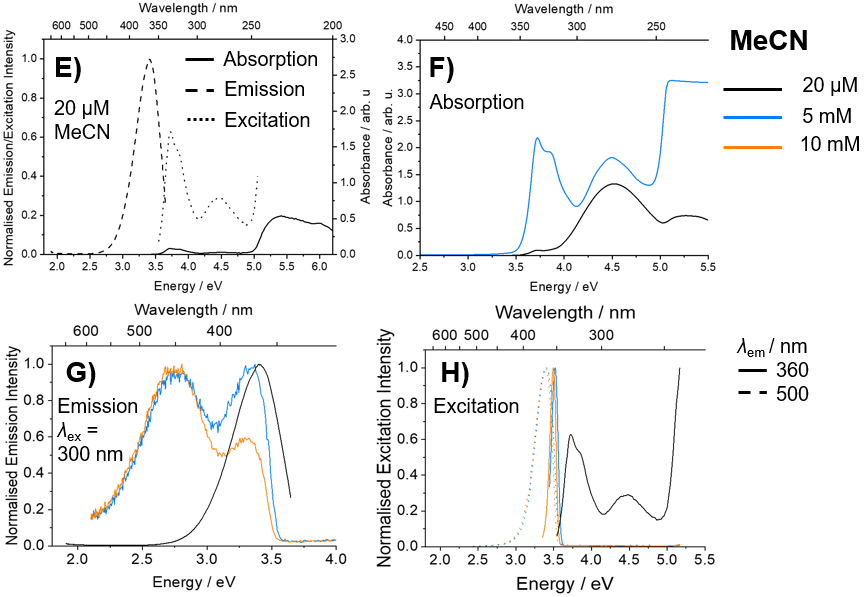
**

**Fig. S32.** Photophysical data for **MeQn·**BF_4_ in deionised water (A–D) and MeCN (E–H) at a range of concentrations: *c* = 20 µM (black), 5 mM (blue) and 10 mM (orange). A,E) 20 µM absorption (solid), emission (dashed) and excitation (dotted) data (*c* = 20 µM). B,F) Absorption data for different concentrations C,G) Emission spectra at a range of concentrations *λ*_ex_ = 300 nm. D,H) Excitation spectra at a range of concentrations *λ*_em_ = 360 nm (solid) and 500 nm (dashed).


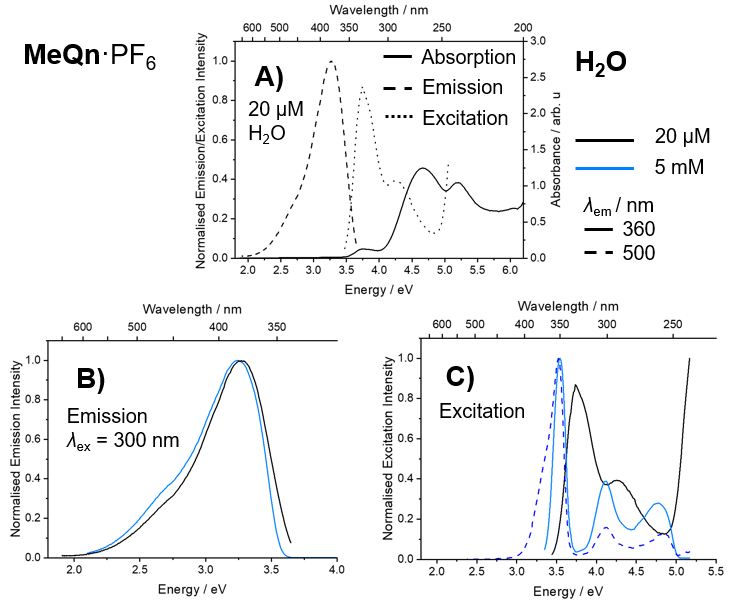


**
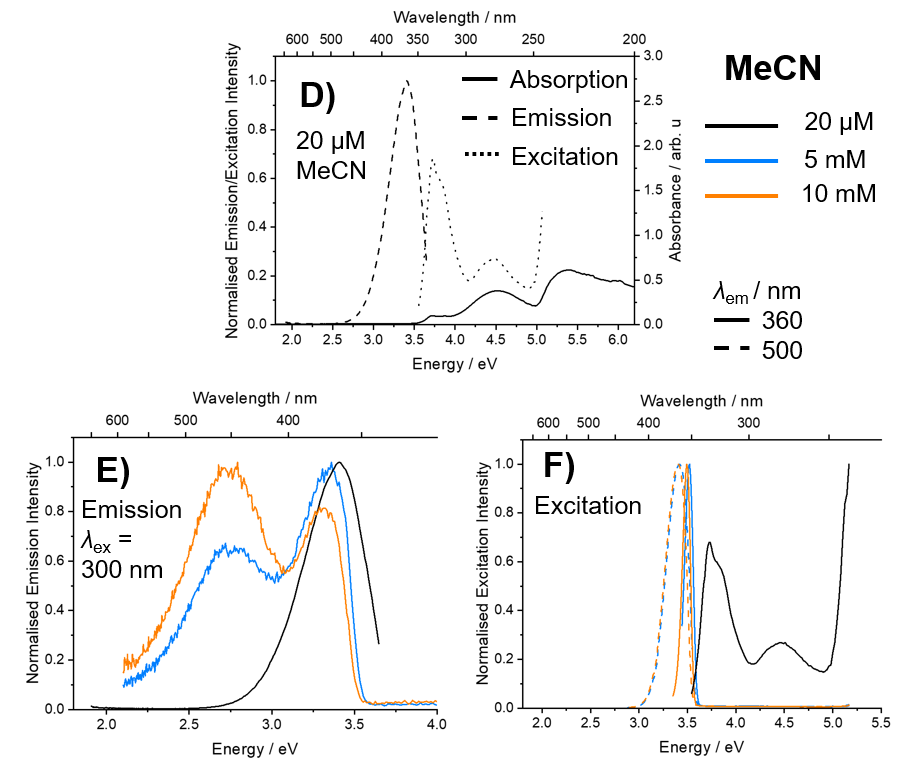
**

**Fig. S33.** Photophysical data for **MeQn·**PF_6_ in deionised water (A–C) and MeCN (D–F) at a range of concentrations: *c* = 20 µM (black), 5 mM (blue) and 10 mM (orange). A,D) absorption (solid), emission (dashed) and excitation (dotted) spectra (*c* = 20 µM). B,E) Emission spectra at a range of concentrations *λ*_ex_ = 300 nm. C,F) Excitation spectra at a range of concentrations *λ*_em_ = 360 nm (solid) and 500 nm (dashed).

**
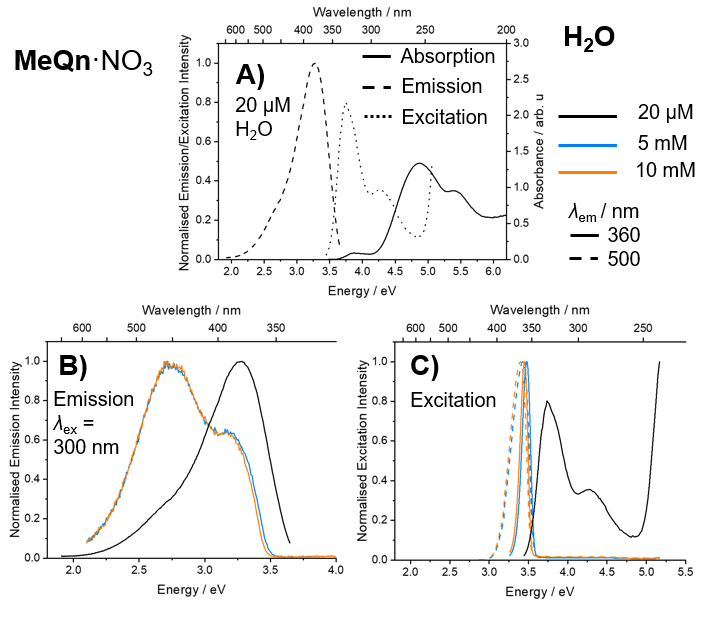
**

**
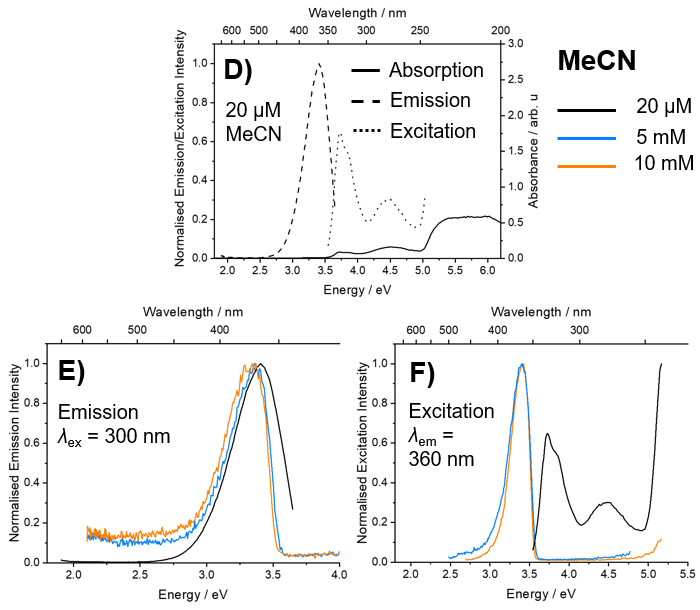
**

**Fig. S34.** Photophysical data for **MeQn·**NO_3_ in water (A–C) and MeCN (D–F) at a range of concentrations: *c* = 20 µM (black), 5 mM (blue) and 10 mM (orange). A,D) absorption (solid), emission (dashed) and excitation (dotted) spectra (*c* = 20 µM). B,E) Emission spectra at a range of concentrations *λ*_ex_ = 300 nm. C,F) Excitation spectra at a range of concentrations *λ*_em_ = 360 nm (solid) and 500 nm (dashed).

**
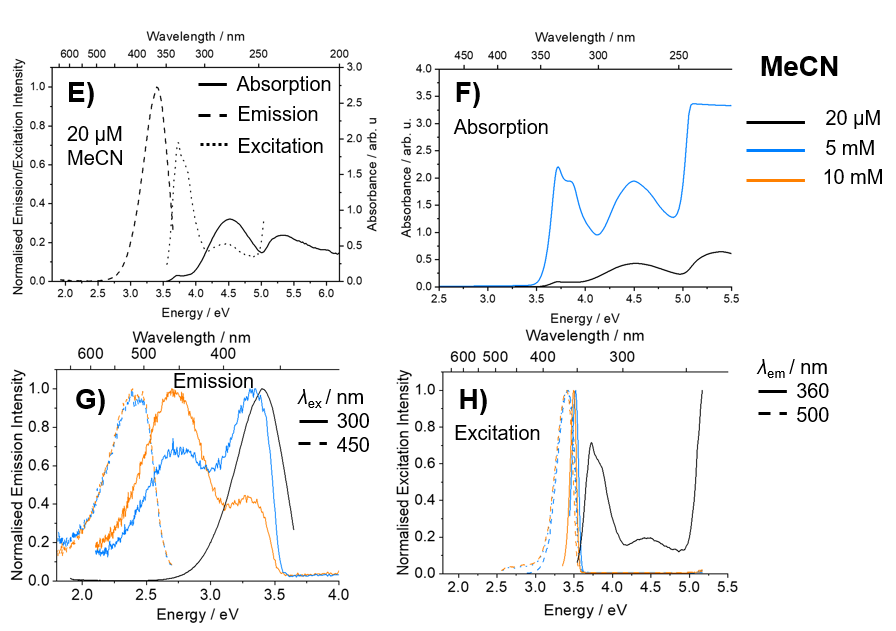
**
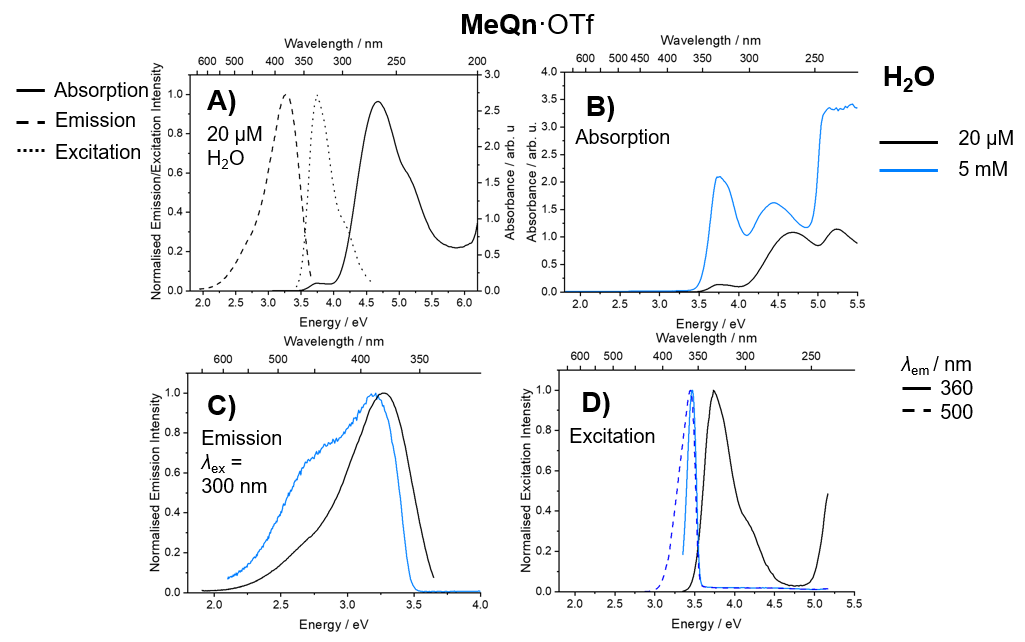


**Fig. S35.** Photophysical data for **MeQn·**OTf in deionised water (A–D) and MeCN (E–H) at a range of concentrations: *c* = 20 µM (black), 5 mM (blue) and 10 mM (orange). A,E) absorption (solid), emission (dashed) and excitation (dotted) spectra (*c* = 20 µM). B,F) Absorption data for different concentrations. C,G) Emission spectra at a range of concentrations *λ*_em_ = 300 nm (solid) and 450 nm (dashed). D&H) Excitation data at a range of concentrations *λ*_em_ = 360 nm (solid) and 500 nm (dashed).

**
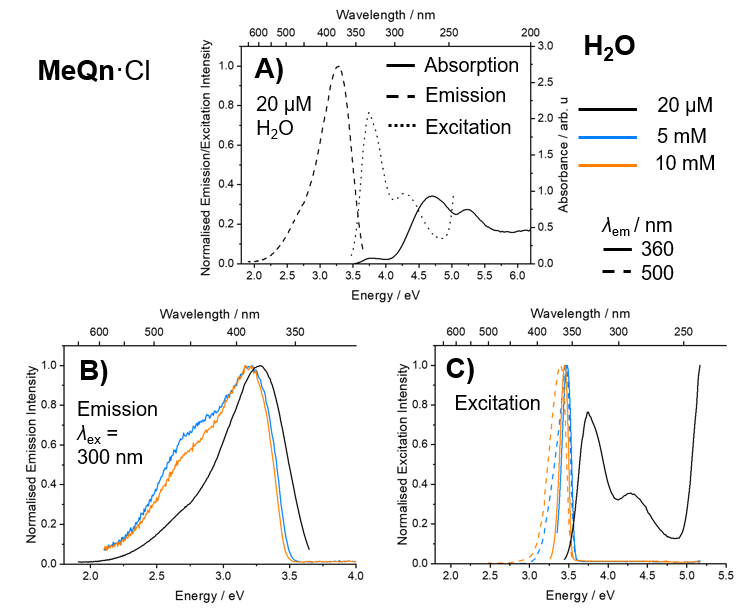
**

**
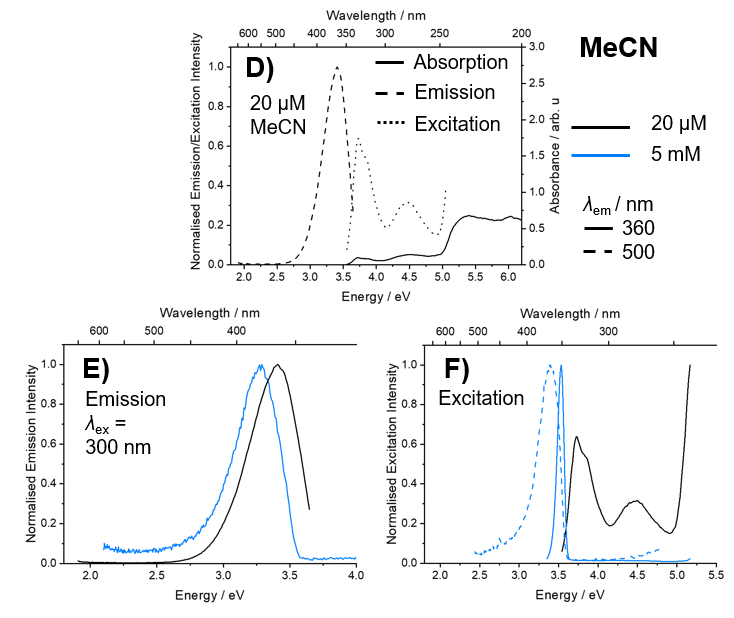
**

**Fig. S36.** Photophysical data for **MeQn·**Cl in deionised water (A–C) and MeCN (D–F) at a range of concentrations: *c* = 20 µM (black), 5 mM (blue) and 10 mM (orange). A,D) absorption (solid), emission (dashed) and excitation (dotted) spectra (*c* = 20 µM). B,E) Emission spectra at a range of concentrations *λ*_ex_ = 300 nm. C,F) Excitation spectra at a range of concentrations *λ*_em_ = 360 nm (solid) and 500 nm (dashed).

**
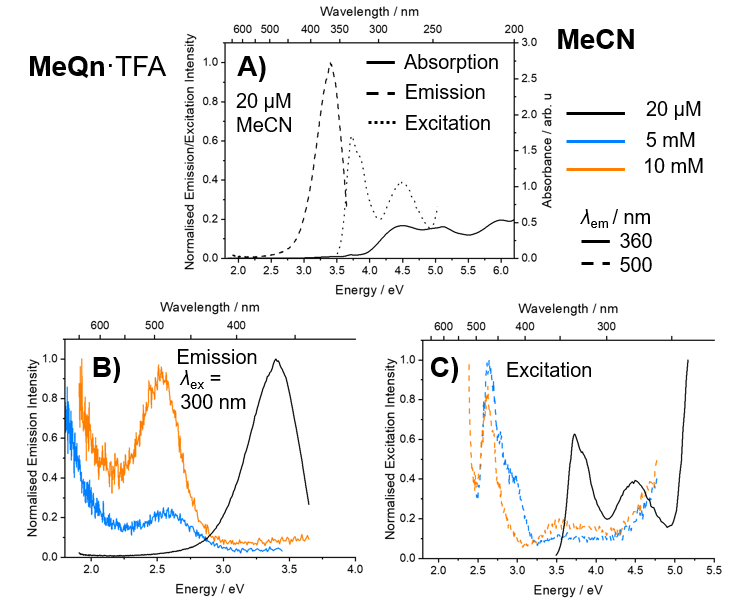
**

**Fig. S37.** Photophysical data for **MeQn·**TFA in MeCN (A–C) at a range of concentrations: *c* = 20 µM (black), 5 mM (blue) and 10 mM (orange). A) absorption (solid), emission (dashed) and excitation (dotted) spectra (*c* = 20 µM). B) Emission spectra at a range of concentrations *λ*_ex_ = 300 nm. C) Excitation spectra at a range of concentrations *λ*_em_ = 360 nm (solid) and 500 nm (dashed).

**
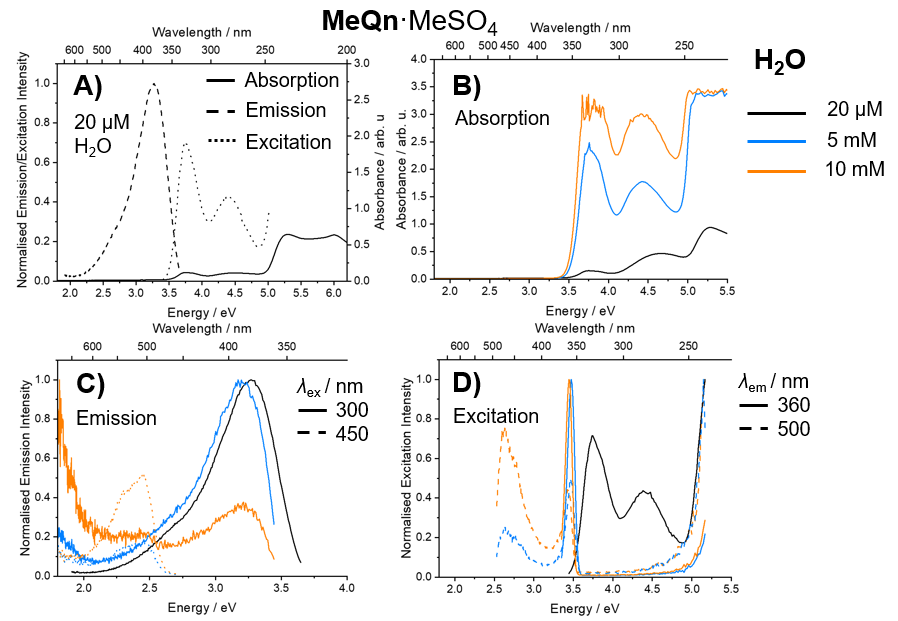
**

**
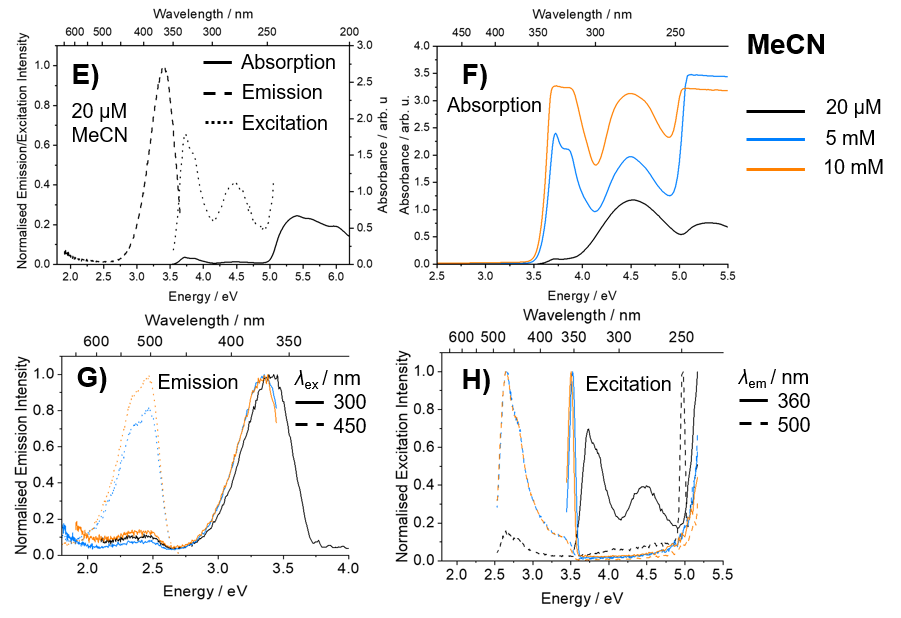
**

**Fig. S38.** Photophysical data for **MeQn·**MeSO_4_ in deionised water (A–D) and MeCN (E–H) at a range of concentrations: *c* = 20 µM (black), 5 mM (blue) and 10 mM (orange). A,E) absorption (solid), emission (dashed) and excitation (dotted) spectra (*c* = 20 µM). B,F) Absorption data for different concentrations. C,G) Emission spectra at a range of concentrations *λ*_em_ = 300 nm (solid) and 450 nm (dashed). D&H) Excitation data at a range of concentrations *λ*_em_ = 360 nm (solid) and 500 nm (dashed).

**
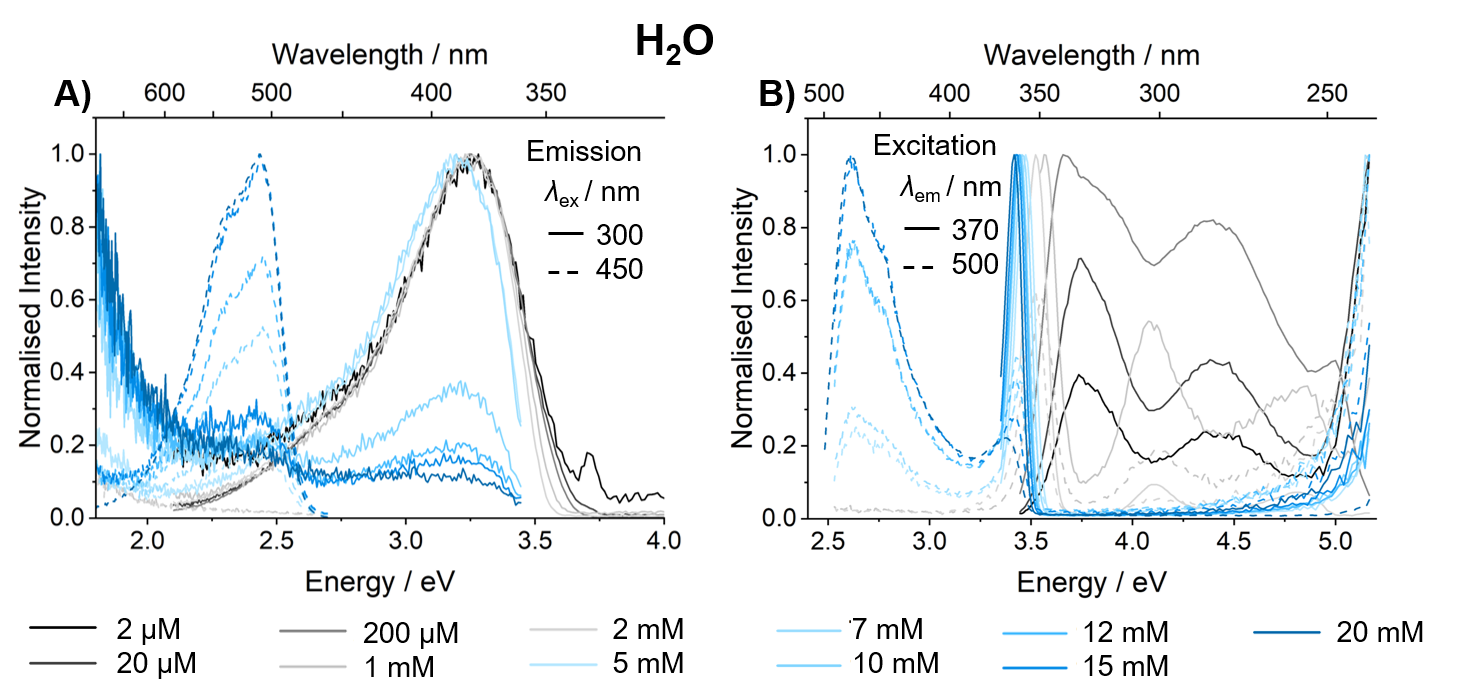
**

**Fig. S39.** Photophysical data for **MeQn·**MeSO_4_ in deionised water at a range of concentrations: *c* = 2 µM (black), 20 µM (dark grey), 200 µM (grey), 1 mM (light grey), 2 mM (palest grey), 5 mM (palest blue), 7 mM (pale blue), 10 mM (blue), 12 mM (dark blue), 15 mM (darker blue) and 20 mM (navy blue). A) Emission spectra at a range of concentrations *λ*_em_ = 300 nm (solid) and 450 nm (dashed). B) Excitation data at a range of concentrations *λ*_em_ = 370 nm (solid) and 500 nm (dashed).

**
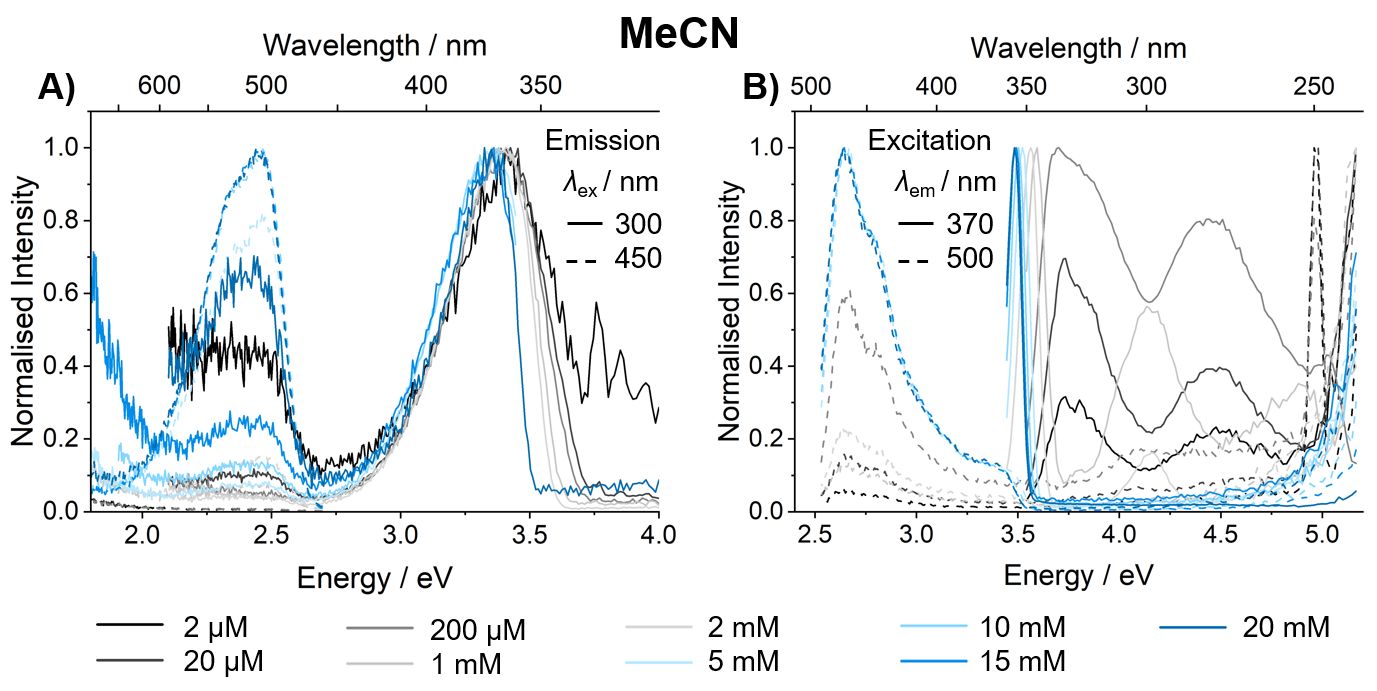
**

**Fig. S40.** Photophysical data for **MeQn·**MeSO_4_ in deionised water at a range of concentrations: *c* = 2 µM (black), 20 µM (dark grey), 200 µM (grey), 1 mM (light grey), 2 mM (palest grey), 5 mM (palest blue), 10 mM (blue), 15 mM (darker blue) and 20 mM (navy blue). A) Emission spectra at a range of concentrations *λ*_em_ = 300 nm (solid) and 450 nm (dashed). B) Excitation data at a range of concentrations *λ*_em_ = 370 nm (solid) and 500 nm (dashed).

**5. X-Ray Crystallographic Analysis**

**
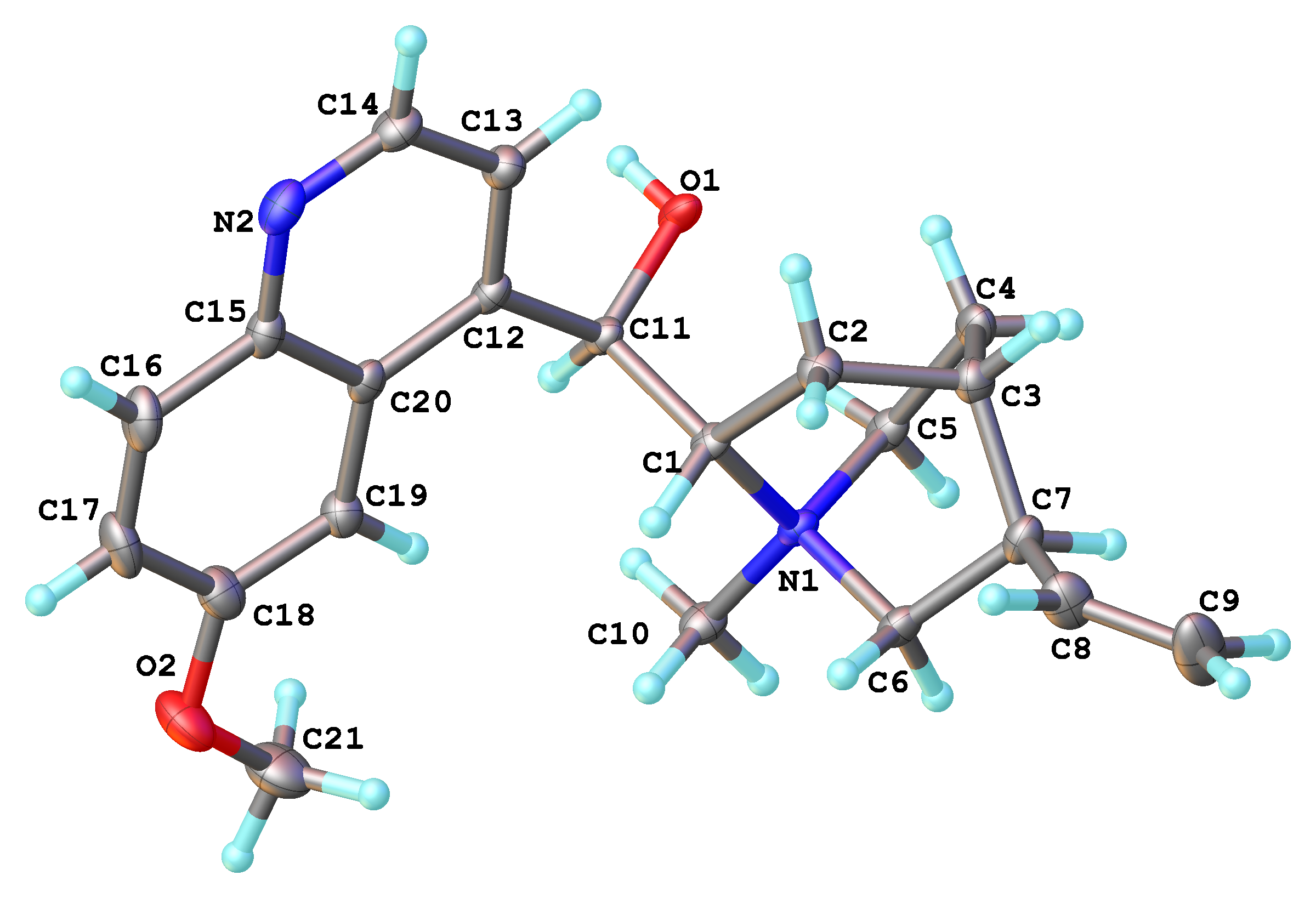
**Crystals of **MeQn**·MeSO_4_ suitable for X-ray diffraction were grown by slow evaporation of a saturated MeCN solution.

**Fig. S41.** Solid-state structure of **MeQn**·MeSO_4_ including probability ellipsoids at 50%. The counterion has been omitted for clarity.

**Table S3.** Crystal data and structure refinement for **MeQn**·MeSO_4_.

| **MeQn**·MeSO_4_ | |
| --- | --- |
| CCDC Deposition Number | 2419115 |
| Empirical formula | C_22_H_27_N_2_O_2_ $\times$CH_3_OSO_3_ $\times$ CH_3_CN |
| Formula weight | 491.59 |
| Temperature/K | 120.0 |
| Crystal system | orthorhombic |
| Space group | P2_1_2_1_2_1_ |
| a/Å | 7.9512(4) |
| b/Å | 14.3111(8) |
| c/Å | 21.8950(12) |
| α/° | 90 |
| β/° | 90 |
| γ/° | 90 |
| Volume/Å^3^ | 2491.4(2) |
| Z | 4 |
| ρ_calc_g/cm^3^ | 1.311 |
| μ/mm^‑1^ | 0.174 |
| F(000) | 1048.0 |
| Crystal size/mm^3^ | 0.34 × 0.26 × 0.09 |
| Radiation | MoKα (λ = 0.71073) |
| 2Θ range for data collection/° | 4.684 to 57.974 |
| Index ranges | -10 ≤ h ≤ 10, -19 ≤ k ≤ 19, -29 ≤ l ≤ 29 |
| Reflections collected | 51830 |
| Independent reflections | 6626 [R_int_ = 0.0526, R_sigma_ = 0.0314] |
| Data/restraints/parameters | 6626/0/395 |
| Goodness-of-fit on F^2^ | 1.052 |
| Final R indexes [I>=2σ (I)] | R_1_ = 0.0352, wR_2_ = 0.0842 |
| Final R indexes [all data] | R_1_ = 0.0412, wR_2_ = 0.0870 |
| Largest diff. peak/hole / e Å^-3^ | 0.36/-0.30 |
| Flack parameter | -0.049(19) |

# 6. Additional TCSPC Data

**
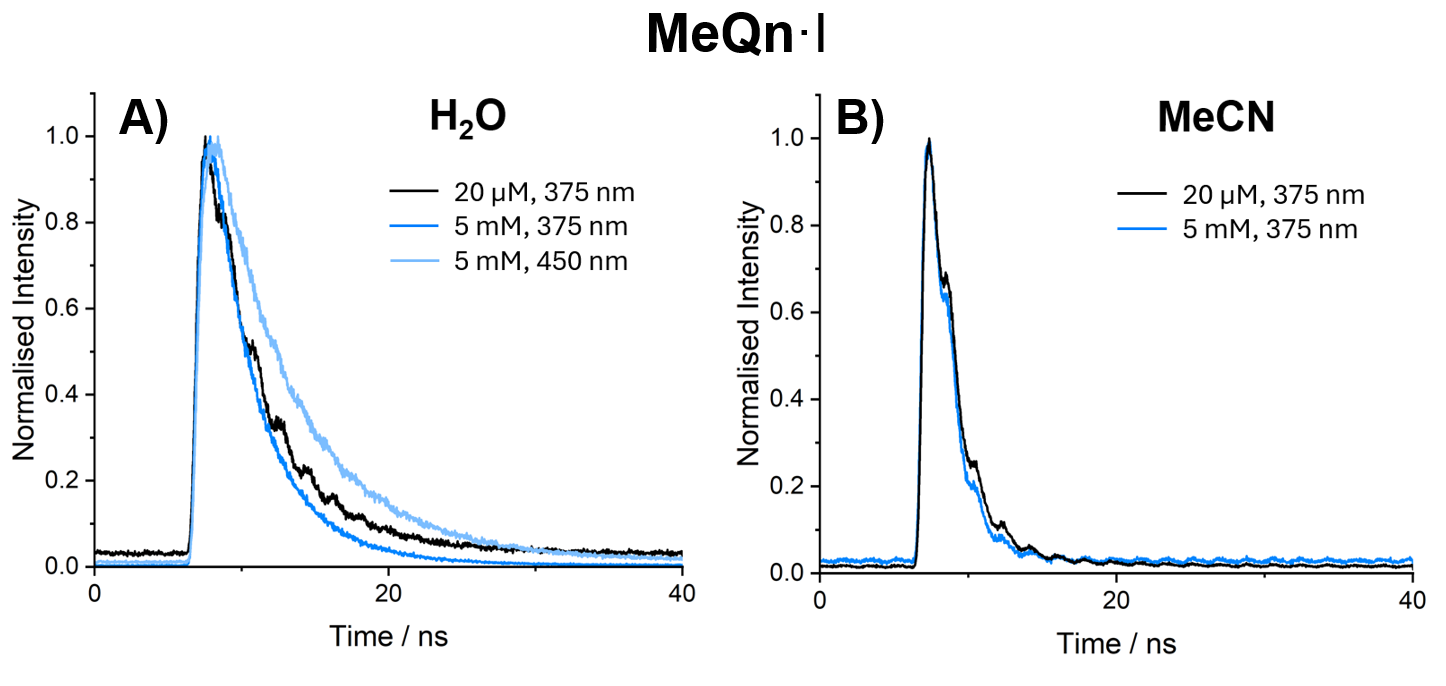
Fig. S42.** TCSPC data for **MeQn·**I in deionised water (A) and MeCN (B), *c* = 20 µM and *λ*_em_ = 375 nm peak (black), *c* = 5 mM and *λ*_em_ = 375 nm peak (dark blue), *c* = 5 mM and *λ*_em_ = 375 nm peak (pale blue) and *c* = 5 mM and *λ*_em_ = 450 nm peak (palest blue).

**
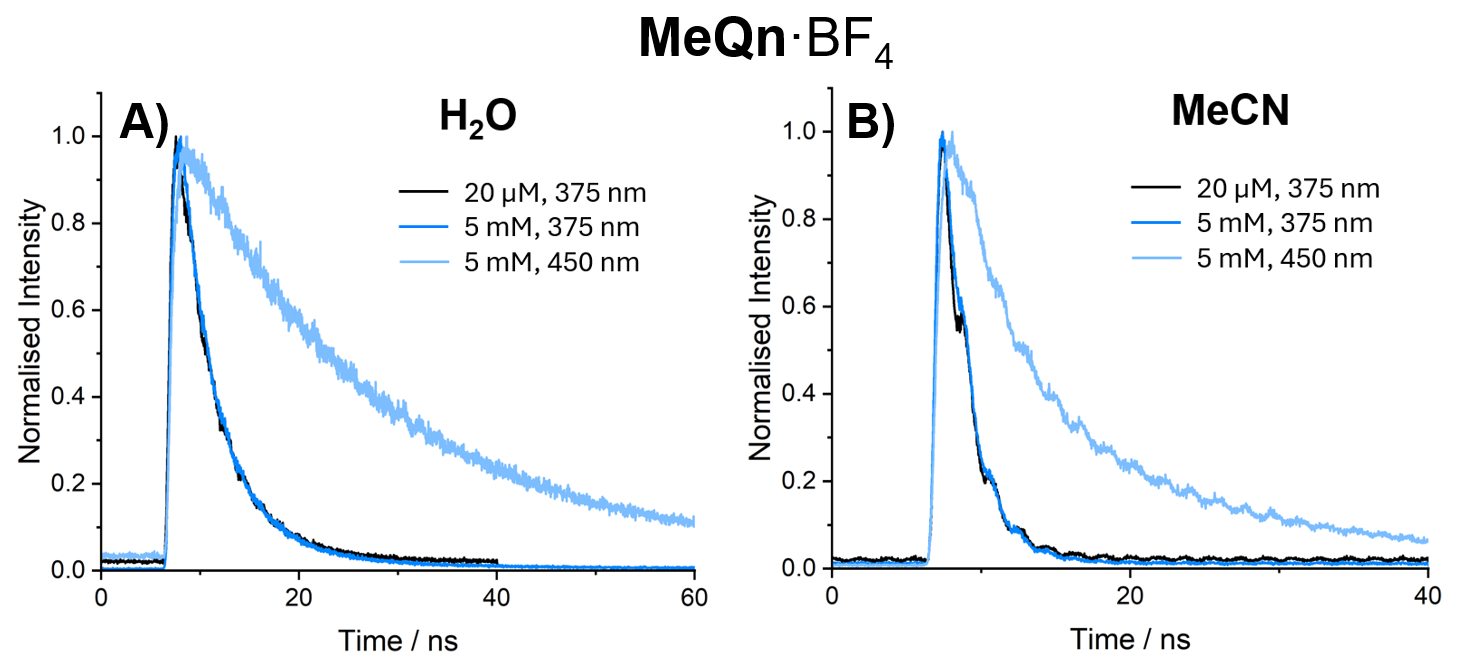
Fig. S43.** TCSPC data for **MeQn·**BF_4_ in deionised water (A) and MeCN (B), *c* = 20 µM and *λ*_em_ = 375 nm peak (black), *c* = 5 mM and *λ*_em_ = 375 nm peak (dark blue), *c* = 5 mM and *λ*_em_ = 375 nm peak (pale blue) and *c* = 5 mM and *λ*_em_ = 450 nm peak (palest blue).

**
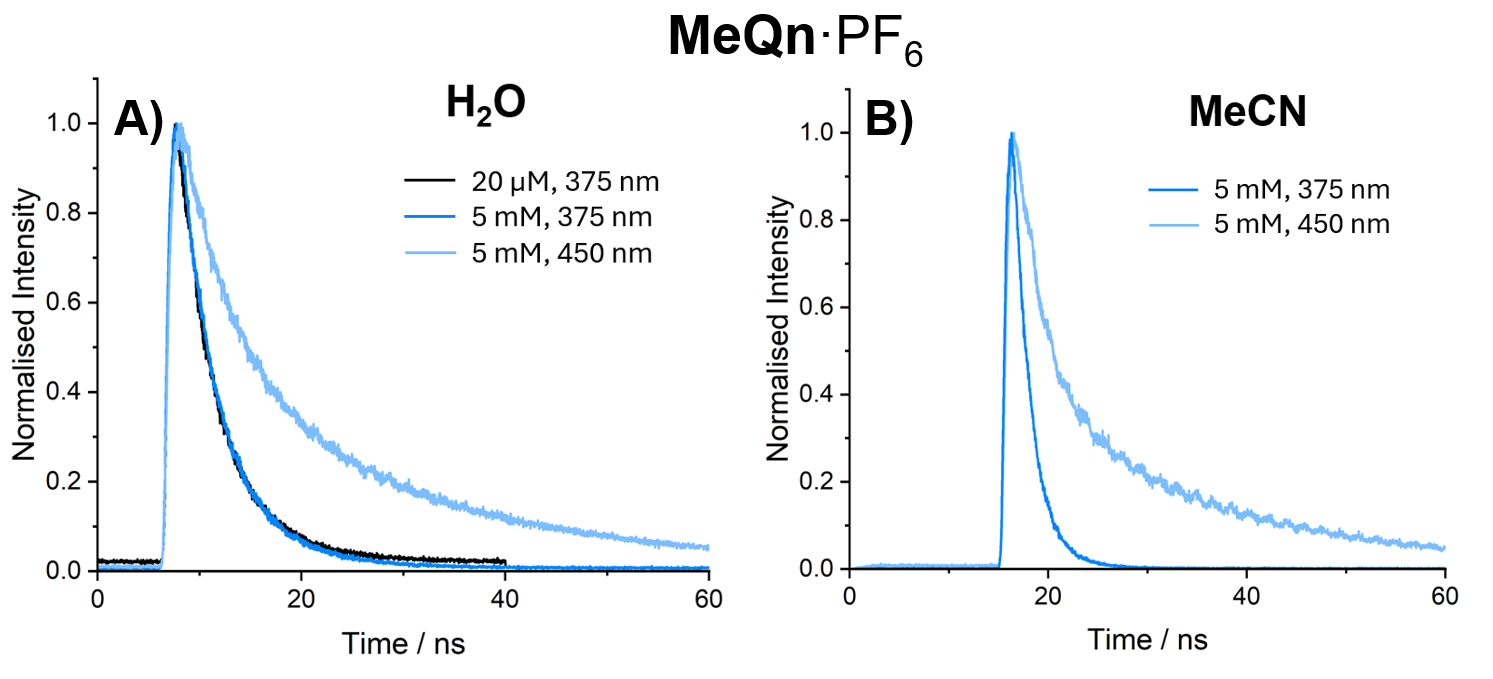
Fig. S44.** TCSPC data for **MeQn·**PF_6_ in deionised water (A) and MeCN (B), *c* = 20 µM and *λ*_em_ = 375 nm peak (black), *c* = 5 mM and *λ*_em_ = 375 nm peak (dark blue), *c* = 5 mM and *λ*_em_ = 375 nm peak (pale blue) and *c* = 5 mM and *λ*_em_ = 450 nm peak (palest blue).

**
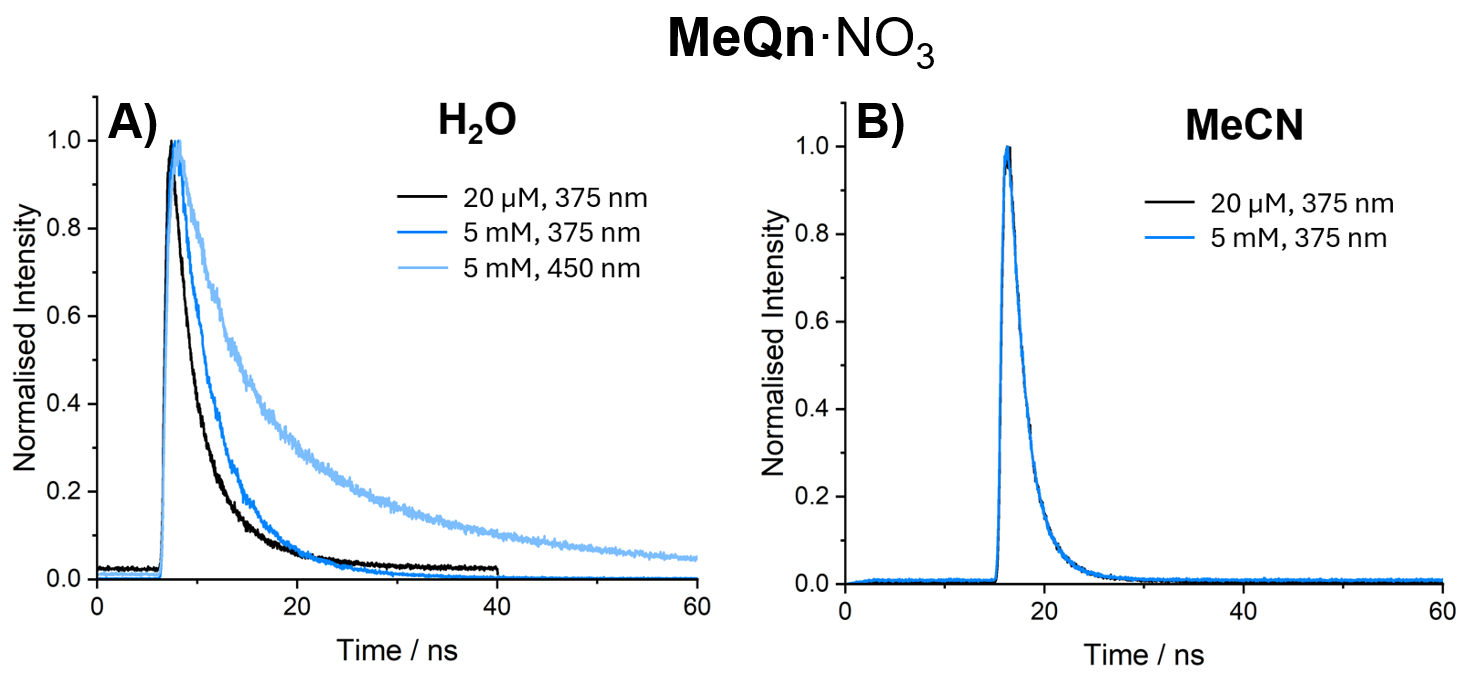
Fig. S45.** TCSPC data for **MeQn·**NO_3_ in deionised water (A) and MeCN (B), *c* = 20 µM and *λ*_em_ = 375 nm peak (black), *c* = 5 mM and *λ*_em_ = 375 nm peak (dark blue), *c* = 5 mM and *λ*_em_ = 375 nm peak (pale blue) and *c* = 5 mM and *λ*_em_ = 450 nm peak (palest blue).

**
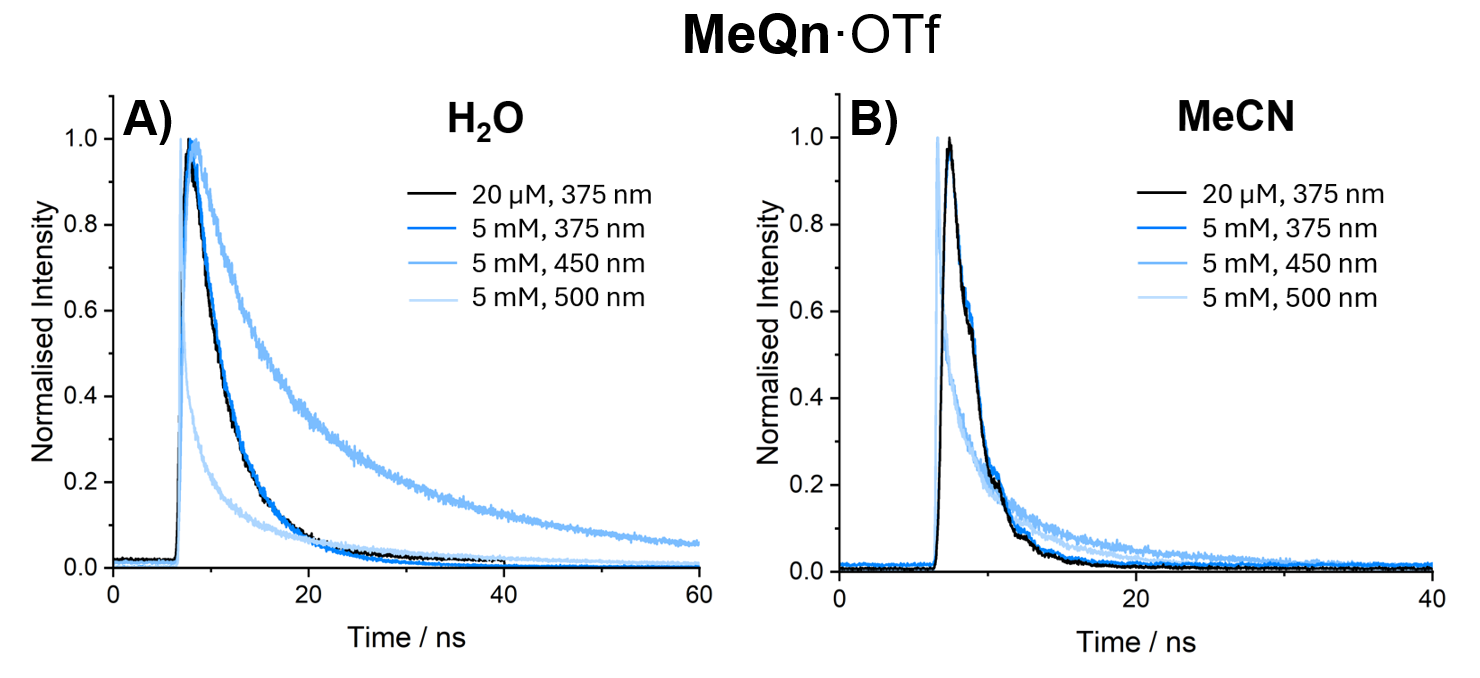
Fig. S46.** TCSPC data for **MeQn·**OTf in deionised water (A) and MeCN (B), *c* = 20 µM and *λ*_em_ = 375 nm peak (black), *c* = 5 mM and *λ*_em_ = 375 nm peak (dark blue), *c* = 5 mM and *λ*_em_ = 375 nm peak (pale blue), *c* = 5 mM and *λ*_em_ = 450 nm peak (paler blue) and *c* = 5 mM and *λ*_em_ = 500 nm peak (palest blue).

**
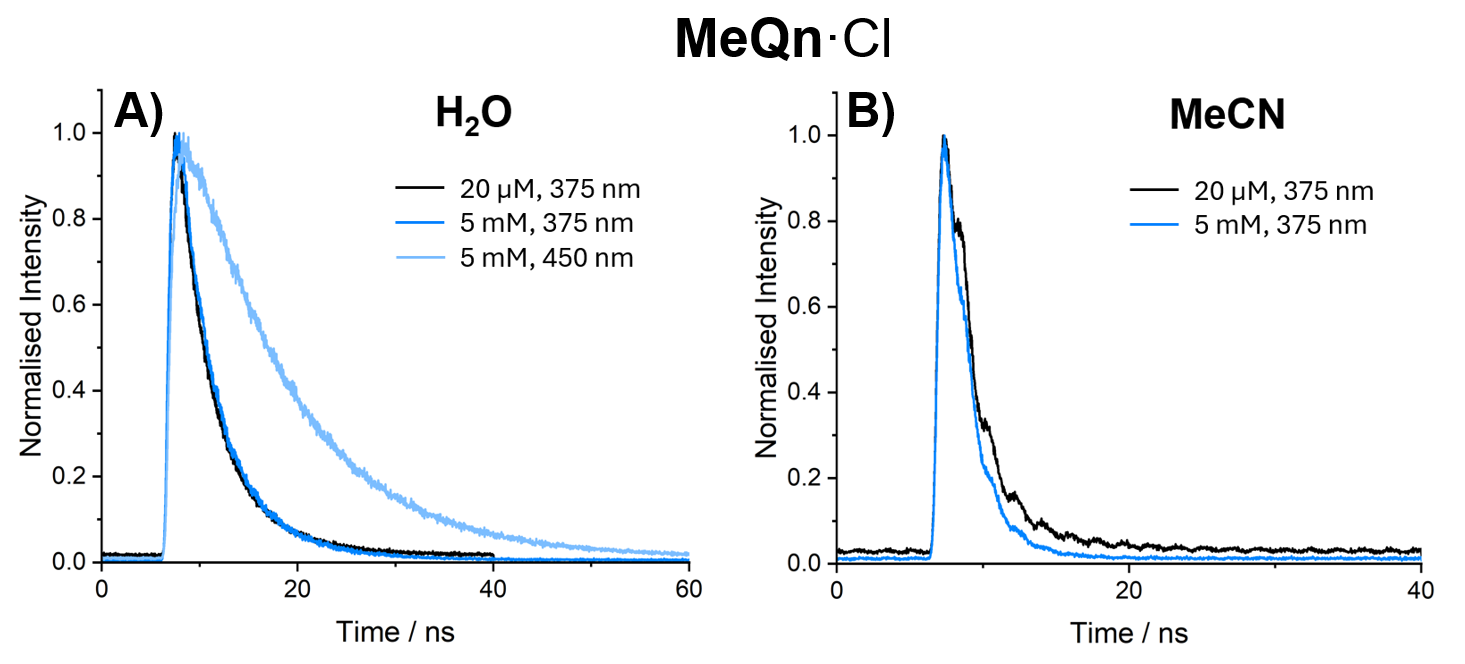
Fig. S47.** TCSPC data for **MeQn·**Cl in deionised water (A) and MeCN (B), *c* = 20 µM and *λ*_em_ = 375 nm peak (black), *c* = 5 mM and *λ*_em_ = 375 nm peak (dark blue), *c* = 5 mM and *λ*_em_ = 375 nm peak (pale blue) and *c* = 5 mM and *λ*_em_ = 450 nm peak (palest blue).

**
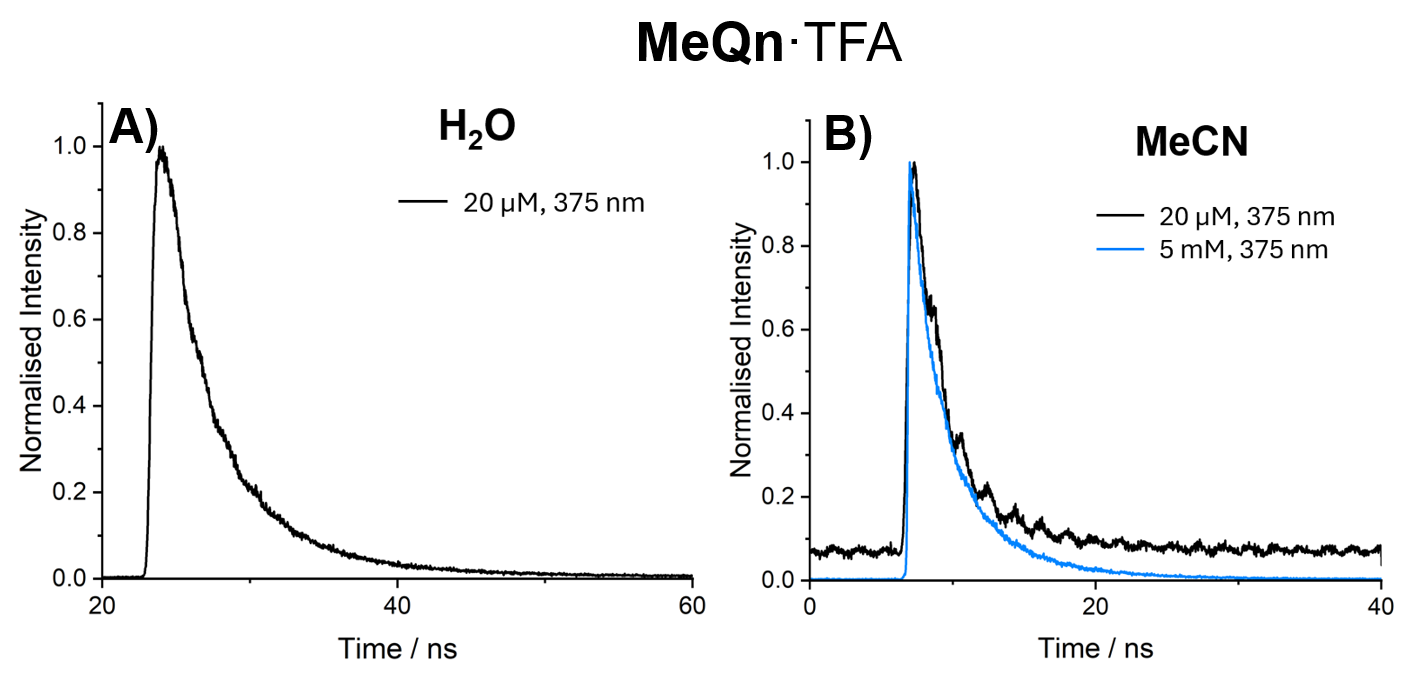
Fig. S48.** TCSPC data for **MeQn·**TFA in deionised water (A) and MeCN (B), *c* = 20 µM and *λ*_em_ = 375 nm peak (black) and *c* = 5 mM and *λ*_em_ = 375 nm peak (blue).

**
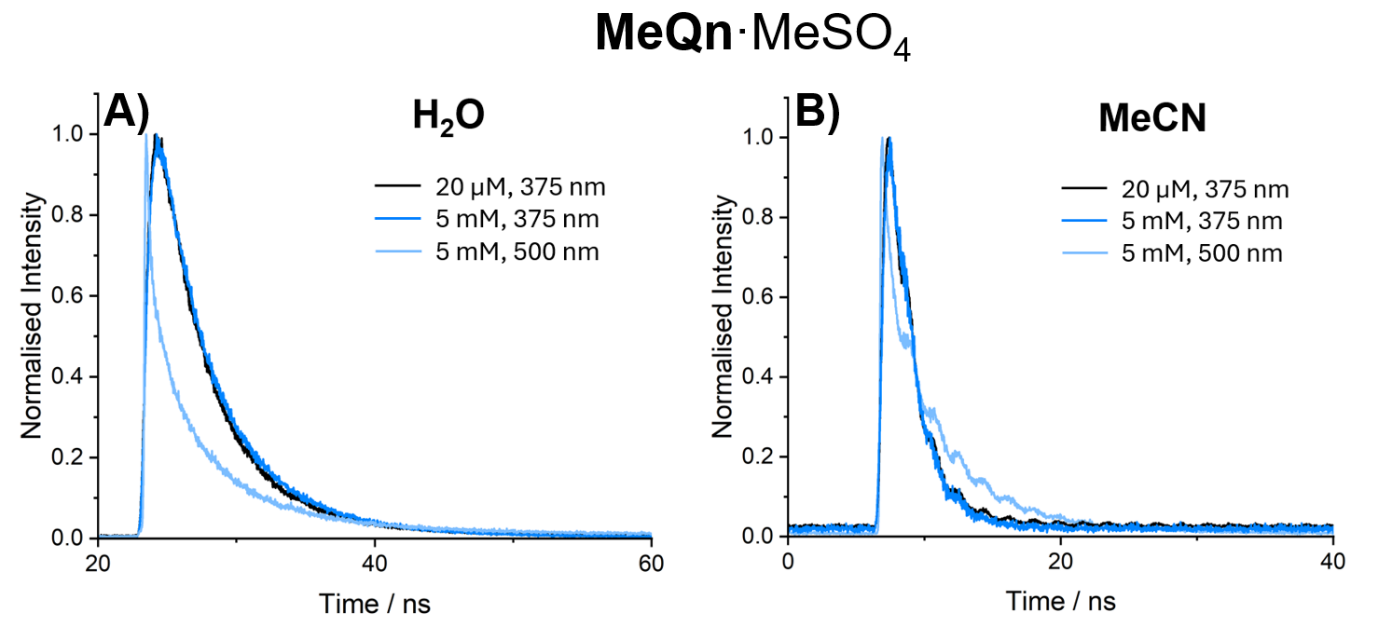
Fig. S50.** TCSPC data for **MeQn·**MeSO_4_ in deionised water (A) and MeCN (B), *c* = 20 µM and *λ*_em_ = 375 nm peak (black), *c* = 5 mM and *λ*_em_ = 375 nm peak (dark blue), *c* = 5 mM and *λ*_em_ = 375 nm peak (pale blue) and *c* = 5 mM and *λ*_em_ = 500 nm peak (palest blue).

**Table S4.** TCSPC data for **MeQn·**X salts (*c* = 20 µM) in MeCN and deionised water.

| 20µM | MeCN | | H_2_O | |
| --- | --- | --- | --- | --- |
| Counterion | t1 | r-squared | t1 | r-squared |
| I | 1.94 | 0.9875 | 4.08 | 0.9935 |
| BF_4_ | 1.84 | 0.9858 | 4.09 | 0.9960 |
| PF_6_ |  |  | 4.05 | 0.9967 |
| OTf (SO_3_CF_3_) | 1.84 | 0.9923 | 4.00 | 0.9971 |
| TFA (C_2_F_3_O_2_) | 2.30 | 0.9822 | 4.01 | 0.9906 |
| Cl | 2.11 | 0.9851 | 3.93 | 0.9973 |
| NO_3_ | 1.90 | 0.9965 | 3.33 | 0.9891 |
| MeSO_4_ | 1.94 | 0.9891 | 4.09 | 0.9971 |

**Table S5.** TCSPC data for **MeQn·**X salts (*c* = 5 mM) in deionised water, measured at 375 nm, 450 nm and 500 nm.

| 5mM H_2_O | 375nm | | 450nm | | 500nm | |
| --- | --- | --- | --- | --- | --- | --- |
| Counterion | t1 | r-squared | t1 | r-squared | t1 | r-squared |
| I | 3.39 | 0.9981 | 5.76 | 0.9978 |  |  |
| BF_4_ | 4.24 | 0.9941 | 20.52 | 0.9964 |  |  |
| PF_6_ | 4.18 | 0.9962 | 14.13 | 0.9853 |  |  |
| OTf (SO_3_CF_3_) | 4.16 | 0.9973 | 14.25 | 0.9866 | 4.76 | 0.9288 |
| TFA (C_2_F_3_O_2_) |  |  |  |  |  |  |
| Cl | 4.19 | 0.9962 | 10.78 | 0.9976 |  |  |
| NO_3_ | 4.23 | 0.9969 | 12.26 | 0.9832 |  |  |
| MeSO_4_ | 4.39 | 0.9972 |  |  | 4.60 | 0.9843 |

**Table S6.** TCSPC data for **MeQn·**X salts (*c* = 5 mM) in MeCN, measured at 375 nm, 450 nm and 500 nm.

| 5mM MeCN | 375nm | | 450nm | | 500nm | |
| --- | --- | --- | --- | --- | --- | --- |
| Counterion | t1 | r-squared | t1 | r-squared | t1 | r-squared |
| I | 1.64 | 0.9850 |  |  |  |  |
| BF_4_ | 1.86 | 0.9924 | 10.03 | 0.9779 |  |  |
| PF_6_ | 1.86 | 0.9971 | 12.70 | 0.9628 |  |  |
| OTf (SO_3_CF_3_) | 1.89 | 0.9925 | 4.56 | 0.9513 | 4.45 | 0.9124 |
| TFA (C_2_F_3_O_2_) |  |  |  |  | 2.95 | 0.9928 |
| Cl | 1.77 | 0.9923 |  |  |  |  |
| NO_3_ | 1.88 | 0.9966 |  |  |  |  |
| MeSO_4_ | 1.87 | 0.9871 |  |  | 3.84 | 0.9867 |

# 7. Additional Photophysical Data

**
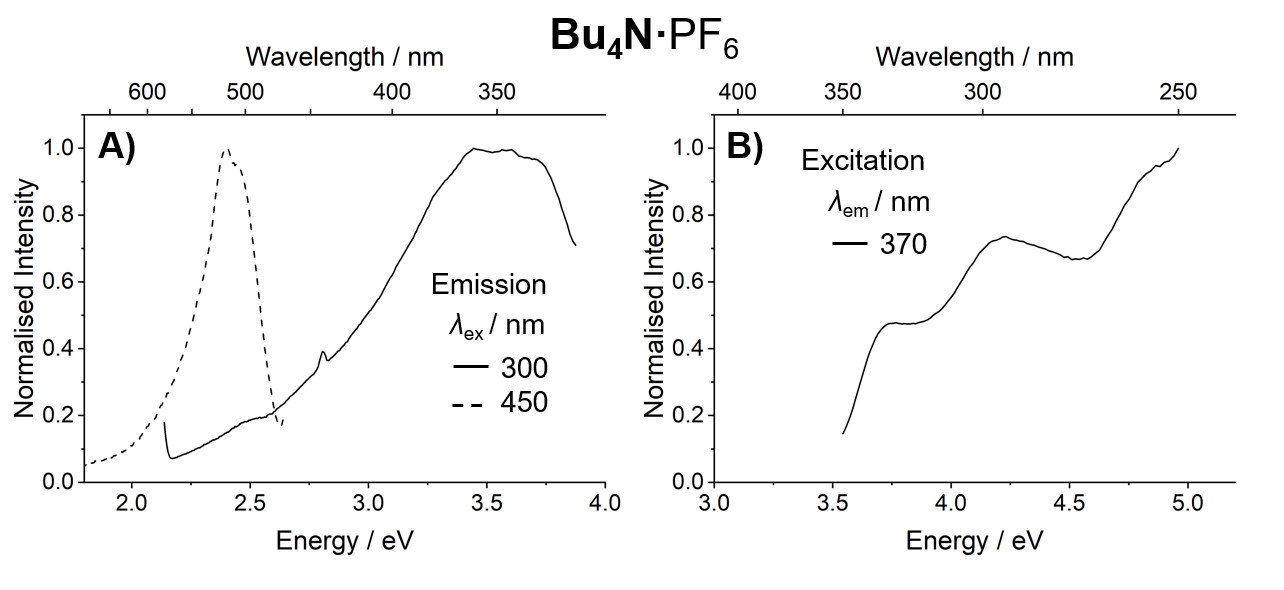
**

**Fig. S50.** Photophysical data for **Bu_4_N·**PF_6_ in MeCN (*c* = 5 mM). A) Emission spectra *λ*_em_ = 300 nm (solid) and 450 nm (dashed). B) Excitation data *λ*_em_ = 370 nm (solid).

**
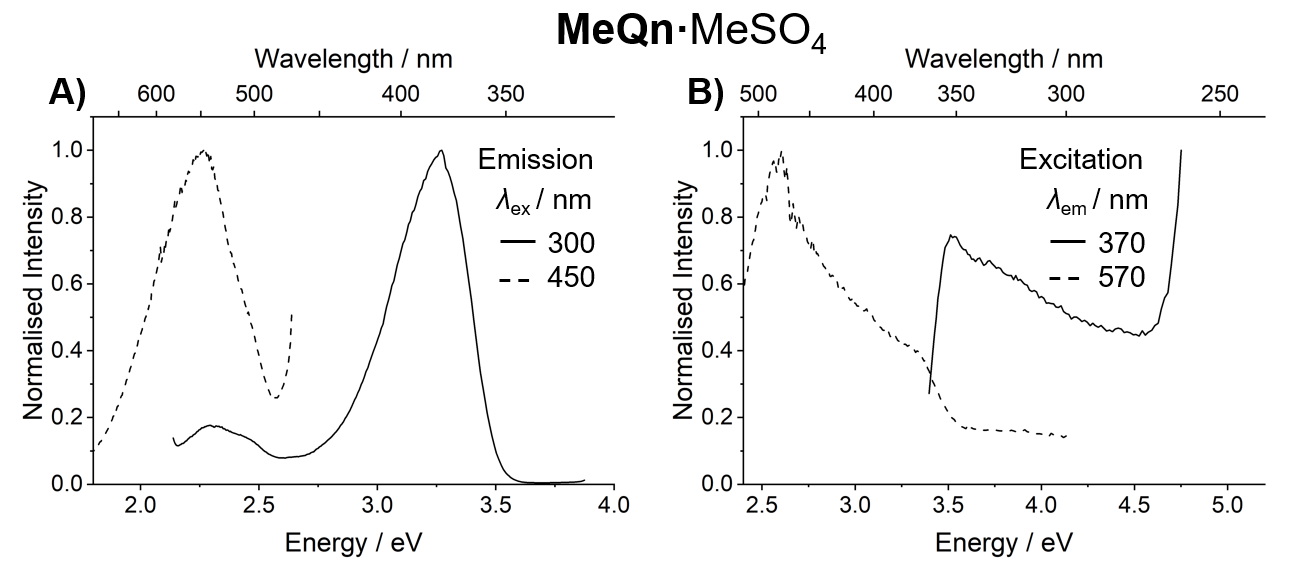
**

**Fig. S51.** Photophysical data for microcrystalline **MeQn·**MeSO_4_. A) Emission spectra *λ*_em_ = 300 nm (solid) and 450 nm (dashed). B) Excitation data *λ*_em_ = 370 nm (solid) and 570 nm (dashed).

**
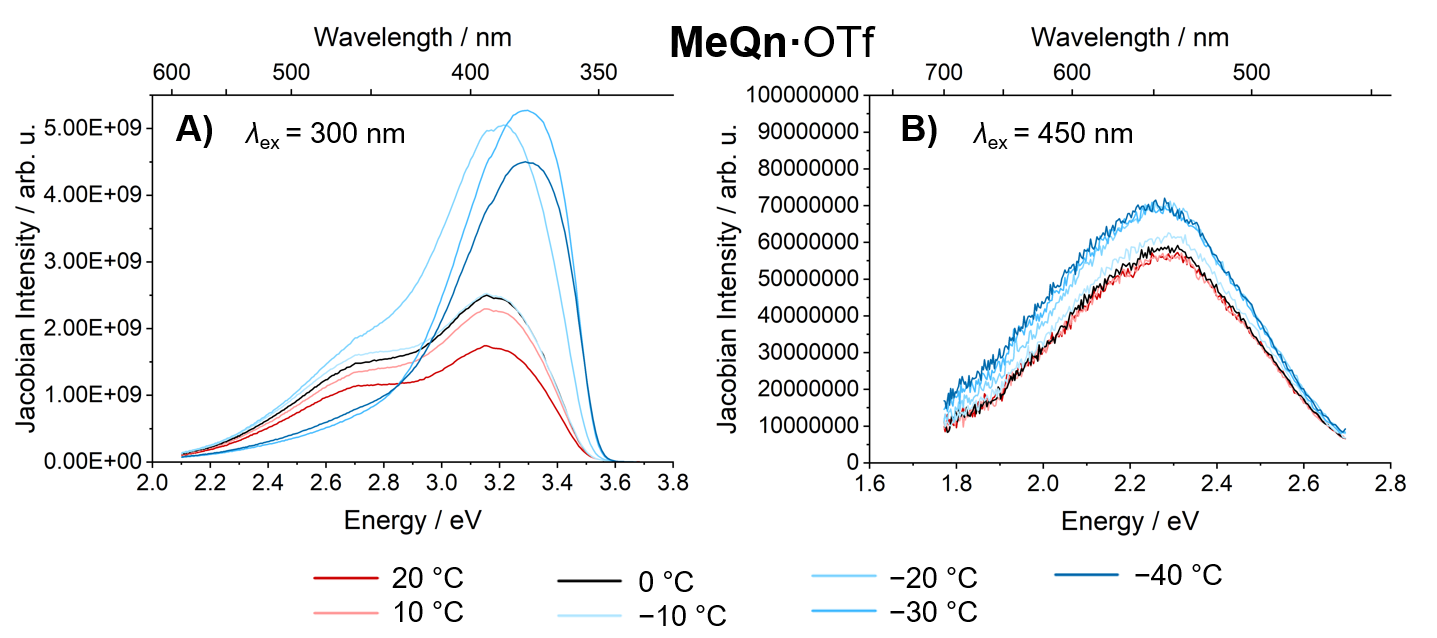
**

**
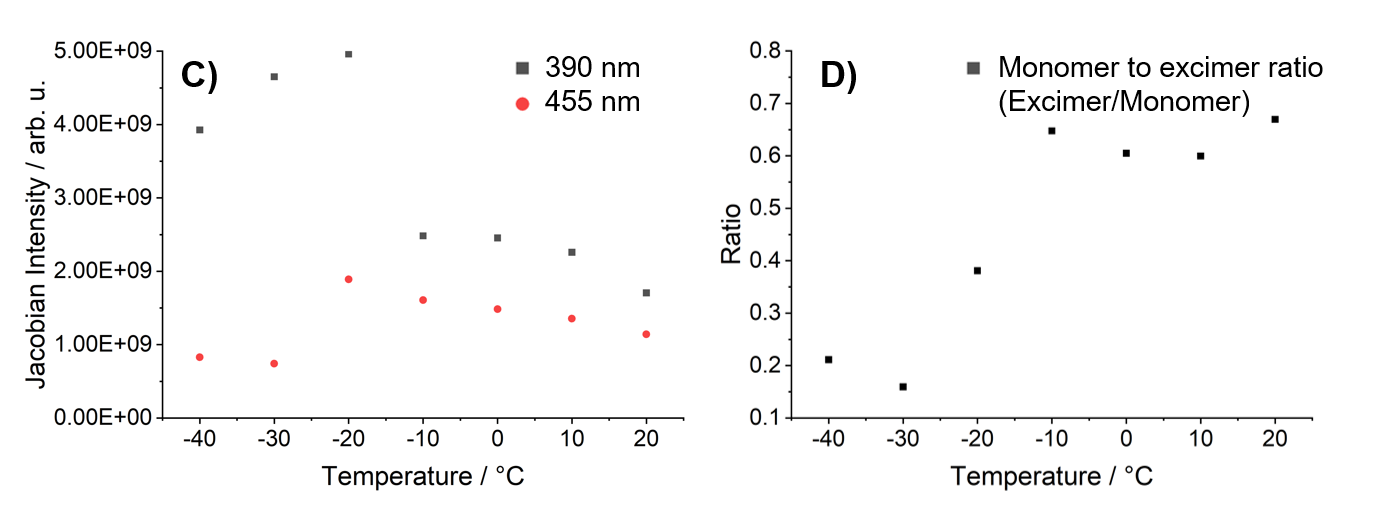
**

**Fig. S52.** A,B) Photophysical data for **MeQn·**OTf in MeCN (*c* = 5 mM) at a range of temperatures: −40 °C (navy blue), −30 °C (darker blue), −20 °C (blue), −10 °C (pale blue), 0 °C (black), 10 °C (pale red), 20 °C (red). A) Emission spectra *λ*_em_ = 300 nm. B) Emission spectra *λ*_em_ = 450 nm. C) Peak intensity of 390 nm (black) and 455 nm (red) peaks at range of temperatures. D) Ratio of monomer to excimer peak intensities (excimer/monomer) at range of temperatures.

**
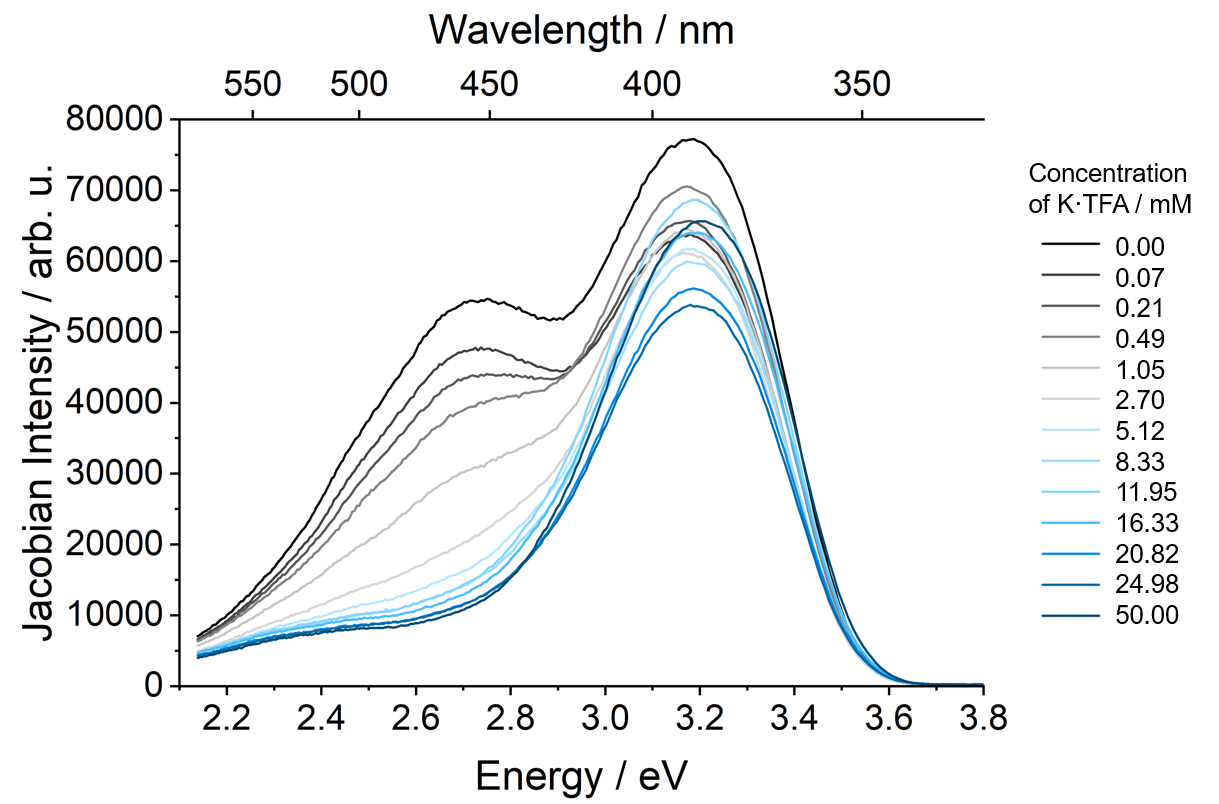
Fig. S53.** Emission data for **MeQn·**PF_6_ in MeCN (*c* = 5 mM, *λ*_ex_ = 300 nm) and upon addition of K·TFA salt additions. Concentrations of KTFA are 0 mM (black), 0.07 mM (darkest grey), 0.21 mM (dark grey), 0.49 mM (grey), 1.05 mM (light grey), 2.70 mM (lightest grey), 5.12 mM (palest blue), 8.33 mM (pale blue), 11.95 mM (blue), 16.33 mM (dark blue), 20.82 mM (darker blue), 24.98 mM (navy blue) and 50 mM (darkest blue).
